# The global burden of type 2 diabetes attributable to high body mass index in 204 countries and territories, 1990–2019: An analysis of the Global Burden of Disease Study

**DOI:** 10.3389/fpubh.2022.966093

**Published:** 2022-09-09

**Authors:** Xuexue Zhang, Xujie Wang, Miaoran Wang, Biaoyan Hu, Wei Tang, Yufei Wu, Jiyu Gu, Tian Ni, Qiuyan Li

**Affiliations:** ^1^Xiyuan Hospital, China Academy of Chinese Medical Sciences, Beijing, China; ^2^Graduate School of China Academy of Chinese Medical Sciences, Beijing, China; ^3^Graduate School of Peking University, Beijing, China

**Keywords:** type 2 diabetes, high body mass index, global burden, mortality, disability adjusted life year

## Abstract

**Background:**

High body mass index (BMI) plays a critical role in the initiation and development of type 2 diabetes (T2D). Up to now, far too little attention has been paid to the global burden of T2D attributable to high BMI. This study aims to report the deaths and disability-adjusted life years (DALYs) of T2D related to high BMI in 204 countries and territories from 1990 to 2019.

**Methods:**

Data on T2D burden attributable to high BMI were retrieved from the Global Burden of Diseases, Injuries, and Risk Factors Study (GBD) 2019. The global cases, age-standardized rates of mortality (ASMR), and disability-adjusted life years (ASDR) attributable to high BMI were estimated by age, sex, geographical location, and socio-demographic index (SDI). The estimated annual percentage change (EAPC) was calculated to quantify the trends of ASMR and ASDR during the period 1990–2019.

**Results:**

Globally, there were 619,494.8 deaths and 34,422,224.8 DALYs of T2D attributed to high BMI in 2019, more than triple in 1990. Moreover, the pace of increase in ASMR and ASDR accelerated during 1990–2019, with EAPC of 1.36 (95% CI: 1.27 to 1.45) and 2.13 (95% CI: 2.10 to 2.17) separately, especially in men, South Asia, and low-middle SDI regions. Oceania was the high-risk area of standardized T2D deaths and DALYs attributable to high BMI in 2019, among which Fiji was the country with the heaviest burden. In terms of SDI, middle SDI regions had the biggest T2D-related ASMR and ASDR in 2019.

**Conclusion:**

The global deaths and DALYs of T2D attributable to high BMI substantially increased from 1990 to 2019. High BMI as a major public health problem needs to be tackled properly and timely in patients with T2D.

## Introduction

Type 2 diabetes (T2D) is a chronic health condition that poses a major global health threat. In 2019, there were an estimated 463 million patients with diabetes globally (1). More seriously, it is expected that there will be more than 590 million patients diagnosed with this condition by 2035 (2). Along with the increase in T2D prevalence, the burden on the health systems increased accordingly. Literature reported that the amount of direct healthcare expenditures attributable to T2D was estimated to be US $760 billion in 2019, 3.28 times more than in 2007 (3).

Overweight and obesity have become a serious public problem. In 2015, obesity affected 603.7 million adults worldwide (4). Also, the global high BMI-related deaths and disability-adjusted life years (DALYs) have substantially increased from 1990 to 2017 (5). Likewise, the burden of disease associated with high BMI was estimated at 216,000 deaths and US$113.9 billion annually in the United States alone (6, 7). Epidemiologic studies have identified high BMI as a major driving factor for T2D (8). Excess adiposity can easily lead to insulin resistance, playing a critical role in the initiation and development of T2D (9). According to the 2019 GBD Study (10), factors such as high BMI, smoking, environmental particles, processed meat, high sugar diet, and low activity were factors that increased the risk of T2D, with high BMI accounting for 51.9% of T2D-related DALYs worldwide and ranking first among all risk factors.

T2D imposes a considerable burden on human beings, and the evidence presented thus far supports the idea that high BMI had the highest proportions of T2D burden (10). Unfortunately, fewer studies make attempt to provide information on the global burden of T2D attributable to high BMI for detail so far (11). On the contrary, the T2D burden caused by high BMI needs to be updated regularly using the most recently available data. DALY is a comprehensive index to measure the loss of life due to premature death and disability and is a key indicator to evaluate the burden of disease (12). Therefore, this study, following the methodology framework and analytical strategies of the GBD collaborative groups, has two primary aims: (1) to compare the global burden of T2D due to high BMI in 21 GBD, 5 SDI regions, and 204 countries and territories from 1990 to 2019; and (2) to evaluate the high BMI-related T2D burden combined gender and age globally between 1990 and 2019.

## Materials and methods

### Data collection

The data source for this investigation was obtained from the Global Health Data Exchange GBD Results Tool (http://ghdx.healthdata.org/gbd-results-tool), which was a collection of information relating to the incidence, prevalence, mortality, and years lived with disability for 369 diseases and injuries, and comparative risks for 87 risk factors in 204 countries and territories from 1990 to 2019. Detailed methodologies for estimating the burden of diseases of GBD 2019 had been reported in previous studies (13, 14). This study was conducted in accordance with the Guidelines for Accurate and Transparent Health Estimates Reporting (GATHER) (Supplementary Table S1) (15). The B.8.1.2 Code was chosen from the GBD Compare for the cause to allow reproducibility of this study. Due to the 2019 GBD, data are publicly available; thus, this study is exempted by the institutional ethics committee (16).

This study included T2D death cases, total DALYs, crude mortality rates, crude DALYs rates, age-standardized rates of T2D mortality (ASMR), and disability-adjusted life years (ASDR) per 100 000 populations attributable to high BMI, and SDI in 204 countries and territories from 1990 to 2019. The DALYs represent the sum of the number of years of life lost due to premature death and years lived with disability, while one DALY can be considered as the loss of 1 year in all healthy life spans (12). SDI is an index proposed in GBD 2015 that presents a geographical location's development status, which is calculated based on lag distributed income per capita, mean education level for people age 15 and older, and total fertility rate under 25 years (17). The 204 countries and territories were categorized into five groups according to SDI quintile: high SDI (>0.805129), high-middle SDI (0.689504–0.805129), middle SDI (0.607679–0.689504), low-middle SDI (0.454743–0.607679), and low SDI (≤0.454743) (18).

### Definitions of Type 2 diabetes and high BMI

Type 2 diabetes was defined as fasting plasma glucose (FPG) ≥ 126 mg/dL (7 mmol/L) or reporting to be on drug or insulin treatment, with the International Classification of Diseases (ICD) version 10 codes: E11-E11.1, E11.3-E11.9 (12). Meanwhile, a BMI >25 kg/ m^2^ in adults (age 20+) and using thresholds based on the International Obesity Task Force standards for patients aged 1–19 years were considered to be high BMI (13, 19).

### Evaluation of Type 2 diabetes burden due to high BMI

Data on high BMI exposure were extracted from systematic literature reviews and reports, covering 2,022 different studies from 190 countries (13). The T2D burden attributable to high BMI was estimated by population attributable fractions (PAFs), which were calculated by a comparative risk assessment framework. The relative risk and the exposure levels in high BMI for T2D were estimated using spatiotemporal Gaussian process regression and many other methods (20, 21).

### Statistical analysis

Estimated annual percentage change (EAPC), which is widely used to measure the age-standardized rate trend over a specific time (22), was performed to evaluate the trend in ASMR and ASDR attributable to high BMI from 1990 to 2019. The calculation methods, which used a linear regression model, were as follows (23):


$$\begin{array}{l} \text{ln}(\text{ASDRorASMR})\;=\text{ α}+\text{βx}+\;\text{ε}\\ \text{ EAPC }=\;100\times (\text{exp}(\text{β})-1) \end{array}$$

In the above equation, x is the calendar year, ε is the error term, and β describes the positive or negative age-standardized rate trend. EAPC and its 95% confidential interval (CI) can be obtained from the above model. When both the value of EAPC and its 95% CI > 0, it is considered to be an increasing trend; when the value of EAPC and its 95% CI all < 0, it is considered to be a declining trend. Otherwise, the burden of T2D due to high BMI is regarded as stable.

In addition, the number of T2D deaths or DALYs, ASMR, or ASDR, and percent change with 95% uncertainty intervals (UIs) were estimated by sex, age, location, and SDI to comprehensively evaluate the burden of T2D attributable to high BMI. The percentage change shown in the Supplementary file between 1990 and 2019 was available on the IHME website (https://vizhub.healthdata.org/gbd-results/). GBD database estimates the number of quantifications more than 1,000 times, of which 95% UIs were defined by the 25th and 975th values of the ordered 1,000 estimates, by DisMod-MR 2.1, a Bayesian meta-regression tool (5). Furthermore, Spearman's rank test was performed to evaluate the relationship between high BMI-related T2D burden (ASMR and ASDR) and SDI in 2019. In addition, the decomposition methodology of Das Gupta (24) was used to decompose global T2D death and DALYs by population age structure, population growth, and epidemiologic changes. All statistical analyses and chart visualization were performed with R soft 3.6.3.

## Results

### Global T2D burden attributable to high BMI from 1990 to 2019

Globally, there were 619,494.8 death cases of T2D attributable to high BMI in 2019, with an ASMR of 7.6 per 100,000 populations, which represents a 52.0% increase since 1990 (Table 1). Moreover, the number of DALYs increased 266.5% in the past 30 years, and the ASDR was 411.1 per 100,000 populations in 2019 (Table 2). From 1990 to 2019, the EAPC in ASMR and ASDR was 1.36 and 2.13 separately, which presents an increased burden of T2D attributable to high BMI.

**Table 1:** Deaths and ASMR of T2D attributable to high BMI in 1990 and 2019 and the temporal trends from 1990 to 2019.

**Deaths**	**1990**		**2019**		**1990–2019**
**Location**	**Deaths cases** **No. (95% UI)**	**ASMR per 100,000** **no. (95% UI)**	**Deaths cases** **no. (95% UI)**	**ASMR per 100,000** **no. (95% UI)**	**EAPC in ASMR no.** **(95% CI)**
Global	191,002.4 (118,333.2–276,040.9)	5.0 (3.0–7.3)	619,494.8 (436,264.6–815,199.8)	7.6 (5.3–10.0)	1.36 (1.27–1.45)
Andean Latin America	1,708.5 (1,143.3–2,320.1)	8.3 (5.4–11.4)	7,204.4 (4,921.5–9,777.7)	12.9 (8.7–17.6)	1.52 (1.37–1.67)
Australasia	1101.4 (723.5–1501.1)	4.7 (3.1–6.5)	2221.1 (1,456.2–3064.3)	4.3 (2.9–5.8)	−0.83 (−1.16–−0.51)
Caribbean	4,163.0 (2,793.0–5,630.3)	16.1 (10.7–21.9)	8,874.4 (6,102.5–12,170)	17.1 (11.7–23.4)	0.03 (−0.06–0.13)
Central Asia	2,200.8 (1,583.3–2,823.8)	4.6 (3.3–5.9)	10,270.1 (7,603.8–12,902.1)	13.6 (10.0–17.2)	3.37 (3.04–3.70)
Central Europe	9,564.8 (6,968–12,048.6)	6.4 (4.6–8.2)	14,663.3 (10,499.6–19,443.5)	6.7 (4.8–8.8)	0.27 (0.14–0.40)
Central Latin America	17,305.4 (11,923.3–22,674.8)	20.6 (13.9–27.3)	56,436 (39,787.1–74,173.8)	23.9 (16.7–31.7)	0.23 (0.09–0.37)
Central Sub–Saharan Africa	2,443.8 (1,275.3–3,848.9)	10.5 (5.3–17)	6,211.2 (3,493.3–9,231.6)	11.5 (6.3–17.3)	−0.14 (−0.46–0.18)
East Asia	12,198.8 (3,818.1–24,261.8)	1.4 (0.4–2.8)	51,443.8 (24,880–82,546.2)	2.5 (1.2–4.1)	2.00 (1.69–2.31)
Eastern Europe	6,429.8 (4,806–7,994.4)	2.3 (1.7–2.8)	12,829.1 (9,409.7–16,438.9)	3.7 (2.7–4.7)	1.01 (0.43–1.60)
Eastern Sub–Saharan Africa	4,606.6 (1,737.7–8,693.7)	6 (2.2–11.7)	16,628.6 (10,103.7–23,976.8)	10.4 (6.1–15.4)	2.03 (1.91–2.16)
High–income Asia Pacific	3,346.8 (1,270.4–5,810.2)	1.7 (0.6–2.9)	4,950 (2,244.5–8,195)	1.1 (0.5–1.8)	−1.86 (−2.15–−1.56)
High–income North America	23,311.6 (15,677.2–30,838)	6.7 (4.6–8.8)	45,226 (32,680.2–57,387.4)	7.2 (5.3–9.0)	−0.42 (−0.92–0.07)
North Africa and Middle East	18,927.5 (13,534.4–24,363.8)	11.5 (8–15.3)	58,204.6 (43,925.5–73,544.8)	14.2 (10.5–18.3)	0.89 (0.77–1.02)
Oceania	976.9 (571.3–1,463.5)	30.2 (16.9–46.5)	3,689.3 (2,369–5,292.2)	48.3 (30–70.6)	1.29 (0.88–1.70)
South Asia	14,295.5 (5,731.5–26,228.6)	2.6 (1–5)	114,922.3 (73564.7–160,889.4)	8.4 (5.3–11.9)	4.22 (3.89–4.56)
Southeast Asia	13,289 (5193.9–23,935.9)	4.8 (1.8–8.9)	84,063.8 (55,195.9–114,708.1)	13.1 (8.4–18.2)	3.78 (3.68–3.88)
Southern Latin America	3,332.5 (1,977.2–4,749.1)	7.2 (4.3–10.4)	7,050.6 (4,655.2–9,556.7)	8.4 (5.6–11.3)	0.02 (−0.29–0.33)
Southern Sub-Saharan Africa	5,609.2 (4,139.5–7,152)	20.9 (15.2–27.2)	20,671 (16,125.8–25,300.6)	39.1 (29.9–48.4)	2.67 (2.18–3.17)
Tropical Latin America	11,059.7 (7,131.2–15,330.2)	12.2 (7.6–17.2)	35,030.1 (25,879–44,973.9)	14.6 (10.7–19.0)	0.65 (0.53–0.77)
Western Europe	29,680.2 (18,734.5–41,999.1)	5.0 (3.2–7.1)	35,266.1 (21,328.6–51,446.3)	3.5 (2.2–5.0)	−1.54 (−1.67–−1.42)
Western Sub-Saharan Africa	5,450.4 (2695.7–8,867.4)	6.3 (3.0–10.6)	23,639.1 (16,016.1–32,387.3)	13.1 (8.6–18.4)	2.58 (2.36–2.81)

No., number; ASMR, age–standardized mortality rate; UI, uncertainty interval; EAPC, estimated annual percentage change; CI, confidential interval.

**Table 2:** DALYs and ASDR of T2D attributable to high BMI in 1990 and 2019 and the temporal trends from 1990 to 2019.

**DALY**	**1990**		**2019**		**1990–2019**
**Location**	**DALYs** **no. (95% UI)**	**ASDR per 100,000** **no. (95% UI)**	**DALYs** **no. (95% UI)**	**ASDR per 100,000** **no. (95% UI)**	**EAPC in ASDR** **no. (95% CI)**
Global	9,390,889.4 (5,778,117.4–13,912,581.4)	224.8 (137.9–332.0)	34,422,224.8 (24,109,732.6–46,307,954.1)	411.1 (287.5–552.2)	2.13 (2.1–2.17)
Andean Latin America	73,747.0 (50,549.7–99,171.1)	332.6 (224.7–450.8)	323,281.7 (235,466.6–423,752.4)	562.0 (407.0–741.4)	1.73 (1.61–1.84)
Australasia	45,394.3 (30,524.2–62,039.7)	197.3 (133.8–267.9)	122,814.3 (84,087.3–169,621.7)	273.7 (188.7–375.9)	0.77 (0.60–0.95)
Caribbean	181,308.4 (123,595.5–245,403.7)	672.1 (455.9–910.1)	448,333.9 (315,288.3–603,289.7)	865.3 (609.0–1,164.8)	0.78 (0.72–0.85)
Central Asia	133,619.1 (93,615.8–181,092.7)	265.8 (185.2–357.2)	553,783.2 (406,685.7–725,347.7)	658.8 (480.1–863.1)	3.04 (2.86–3.23)
Central Europe	540,562.9 (379,348.4–726,001.2)	362.5 (254.2–485.3)	986,895.3 (693,844.4–1,330,663.3)	504.3 (352.6–681.5)	1.23 (1.13–1.34)
Central Latin America	798,146.7 (555,830.3–1,057,032.9)	861.5 (591.3–1149.7)	2,726,715.2 (1963572.8–3597040.7)	1,110.7 (797.2–1,468.9)	0.71 (0.61–0.81)
Central Sub–Saharan Africa	101,859.3 (55,248.3–156,131.5)	379.8 (202.9–582.5)	317,313 (191,729.3–462,253.2)	485.7 (286.3–706.5)	0.47 (0.15–0.79)
East Asia	843,329.8 (245,394.7–1,721,528.0)	84.8 (24.7–173.6)	3,924,324.4 (2,027,219.0–6,197,834.2)	184.3 (95.4–290.4)	2.89 (2.67–3.11)
Eastern Europe	489,333.6 (346,743.8–656,387.8)	175.5 (123.6–236.1)	855,318.7 (607,212.1–1,141,965.9)	260.9 (186.9–347.6)	1.16 (1.02–1.30)
Eastern Sub–Saharan Africa	181,997 (72,134.9–336,753.2)	206.8 (80.2–386.2)	741,217.3 (477,816.4–1,040,490.1)	383.4 (240.3–546.1)	2.34 (2.22–2.46)
High–income Asia Pacific	218,221.5 (84,458.8–387,108.0)	104.9 (40.6–186.0)	441,575.5 (202,189.7–749,226.3)	133.4 (63.2–221.4)	0.38 (0.23–0.54)
High–income North America	1,197,644.6 (823,952.7–1,601,733.3)	367 (255.6–487.6)	3,024,600.8 (2,136,179–4,008,522.8)	533.2 (383–701.8)	1.56 (1.39–1.72)
North Africa and Middle East	843,133.1 (606,695.4–1,107,012.1)	444 (315.6–590)	3,409,078.6 (2,519,585–4,482,696.1)	710.1 (524–939.1)	1.89 (1.76–2.02)
Oceania	40,649.9 (24,841.2–59,452.8)	1,094.2 (655.2–1,623.3)	161,567.1 (108,279.0–22,5083.7)	1,801.3 (1,190.7–2,543.1)	1.42 (1.05–1.80)
South Asia	807,711.8 (330,850.2–1,473,733.4)	120.5 (48.5–223.5)	6,610,911.4 (4,259,423.5–9,189,695.6)	428.8 (277.0–596.9)	4.46 (4.29–4.64)
Southeast Asia	599,805.9 (249579.2–1,054,975.9)	195.2 (79.3–350.0)	3,822,749.8 (2,599,264.9–5,130,101.7)	555.2 (373.3–751.2)	3.77 (3.70–3.84)
Southern Latin America	127,638.3 (76,939.6–183,346.3)	271.4 (163.2–390.0)	348,694.9 (235,834.1–486,112.8)	430.9 (292.7–595.9)	1.24 (1.03–1.45)
Southern Sub–Saharan Africa	207,852.6 (160,904.4–258151.4)	702 (534.9–879.3)	730,196 (587,441.6–891,954.3)	1,235.3 (982.1–1,515.2)	2.35 (1.96–2.75)
Tropical Latin America	515443.1 (336,991.7–725,310.7)	508.1 (330.6–721.2)	1,613,388.3 (1,202,693.6–2,090,285.7)	648.1 (483.8–841.6)	0.93 (0.86–1.01)
Western Europe	1,233,420.7 (790491.1–1,742,299.3)	225.2 (145.1–317.9)	2,266,197.2 (1,467,140.4–3,317,694.9)	301.0 (195.8–432.6)	0.84 (0.77–0.90)
Western Sub–Saharan Africa	210,069.8 (109,427.0–330,400.8)	215.6 (110.9–345.8)	993,268.1 (692,015.3–1,325,998.9)	462.8 (316.7–627.9)	2.66 (2.46–2.86)

DALYs, disability–adjusted life–years; No., number; ASDR, age–standardized DALY rate; UI, uncertainty interval; EAPC, estimated annual percentage change; CI, confidential interval.

**Figure 1:**
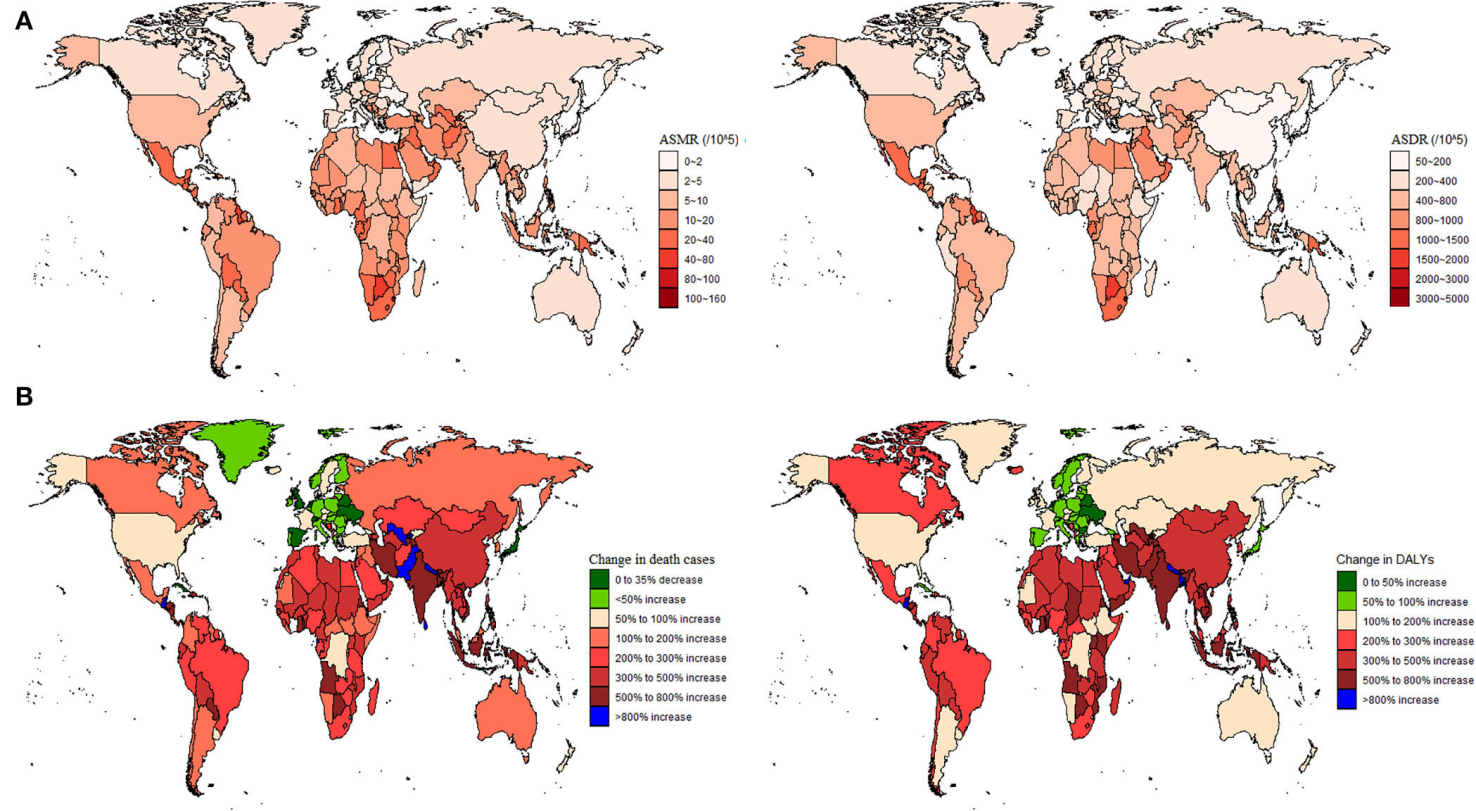
Global type 2 diabetes burden attributable to high BMI in 204 countries and territories. **(A)** Age-standardized mortality rate (ASMR) and age-standardized disability-adjusted life year rate (ASDR) in 2019; and **(B)** The number of deaths and DALYs changed between 1990 and 2019.

**Figure 2:**
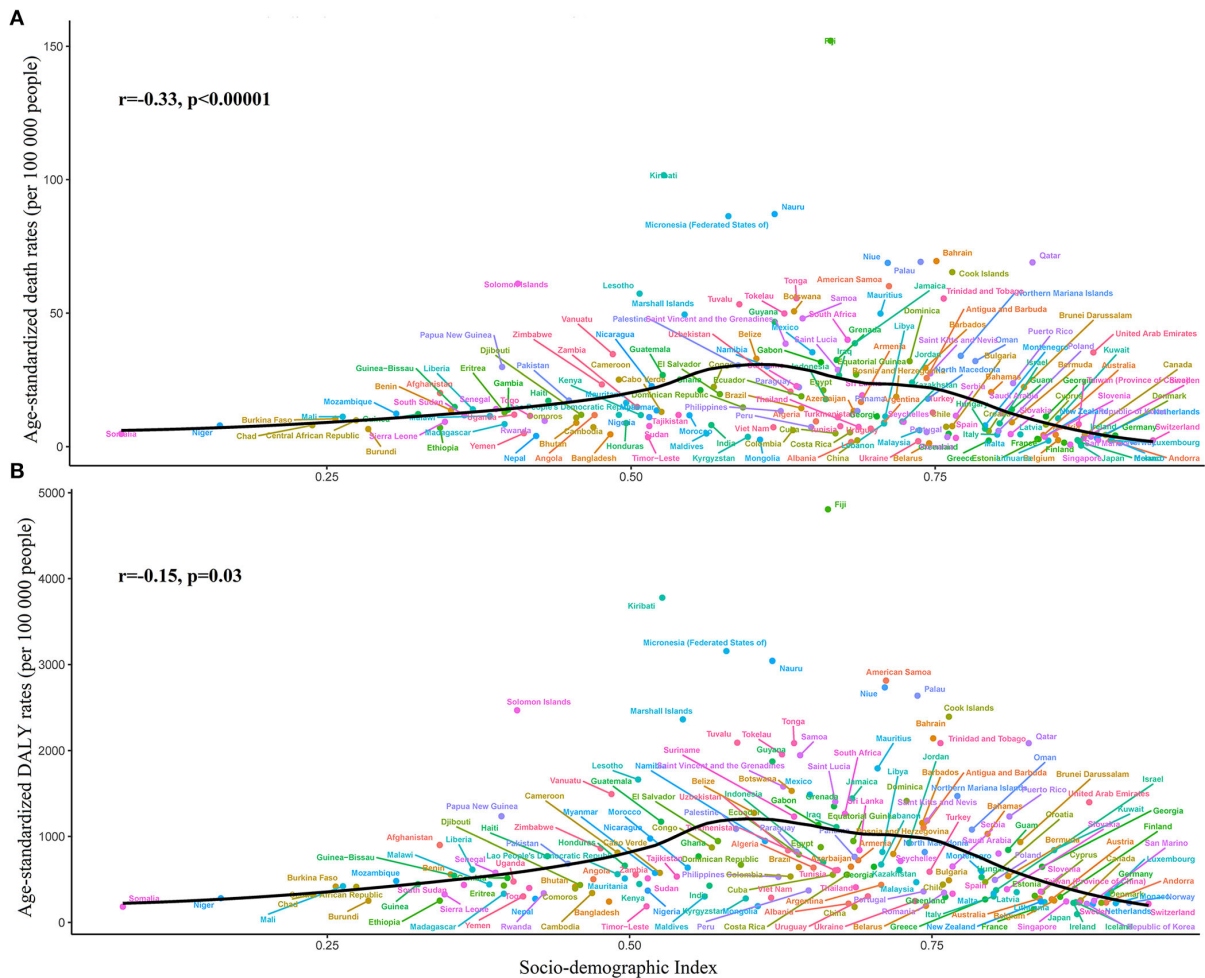
Global type 2 diabetes burden attributable to high body mass index across 204 countries and territories by the socio-demographic index for both sexes combined in 2019. **(A)** Age-standardized mortality rate (ASMR); and **(B)** Age-standardized disability-adjusted life year rate (ASDR).

From the perspective of 204 countries and territories, the highest T2D ASMR, and ASDR attributable to high BMI in 2019 occurred in Fiji (152.1 and 4,806.7 per 100,000 populations), followed by Kiribati (101.7 and 3,777.9 per 100,000 populations), whereas the lowest ASMR and ASDR were found in Japan (0.4 and 97.2 per 100,000 populations) (Figures 1A, 2; Supplementary Tables S2,S3). Between 1990 and 2019, Guatemala experienced the largest percentage increase in the ASMR of high BMI-related T2D burden (540.0%) among all countries, whereas Singapore was the country with the biggest percentage decline (−80.0%) (Supplementary Table S2). Nevertheless, the country with the largest percentage increase in the ASDR was Nepal (413.4%), followed by Guatemala (401.5%), while the country with the biggest percentage decline was Cyprus (−13.6%) (Supplementary Table S3). The number of T2D deaths due to high BMI decreased only in the United Kingdom (−13.3%), Japan (−25.4%), and Belarus (−30.6%) during the period 1990–2019. For all 204 countries and territories, the number of DALYs had substantially increased between 1990 and 2019, with a maximum rate of increase in Guatemala (1,296.3%) (Figure 1B). In addition, a significant negative correlation between the ASMR (*r* =-0.33, *p* < 0.00001) and ASDR (*r* = −0.15, *p* = 0.03) attributable to high BMI and SDI in 2019 was observed (Figure 2).

### T2D burden attributable to high BMI by 21 GBD regions

**Figure 3:**
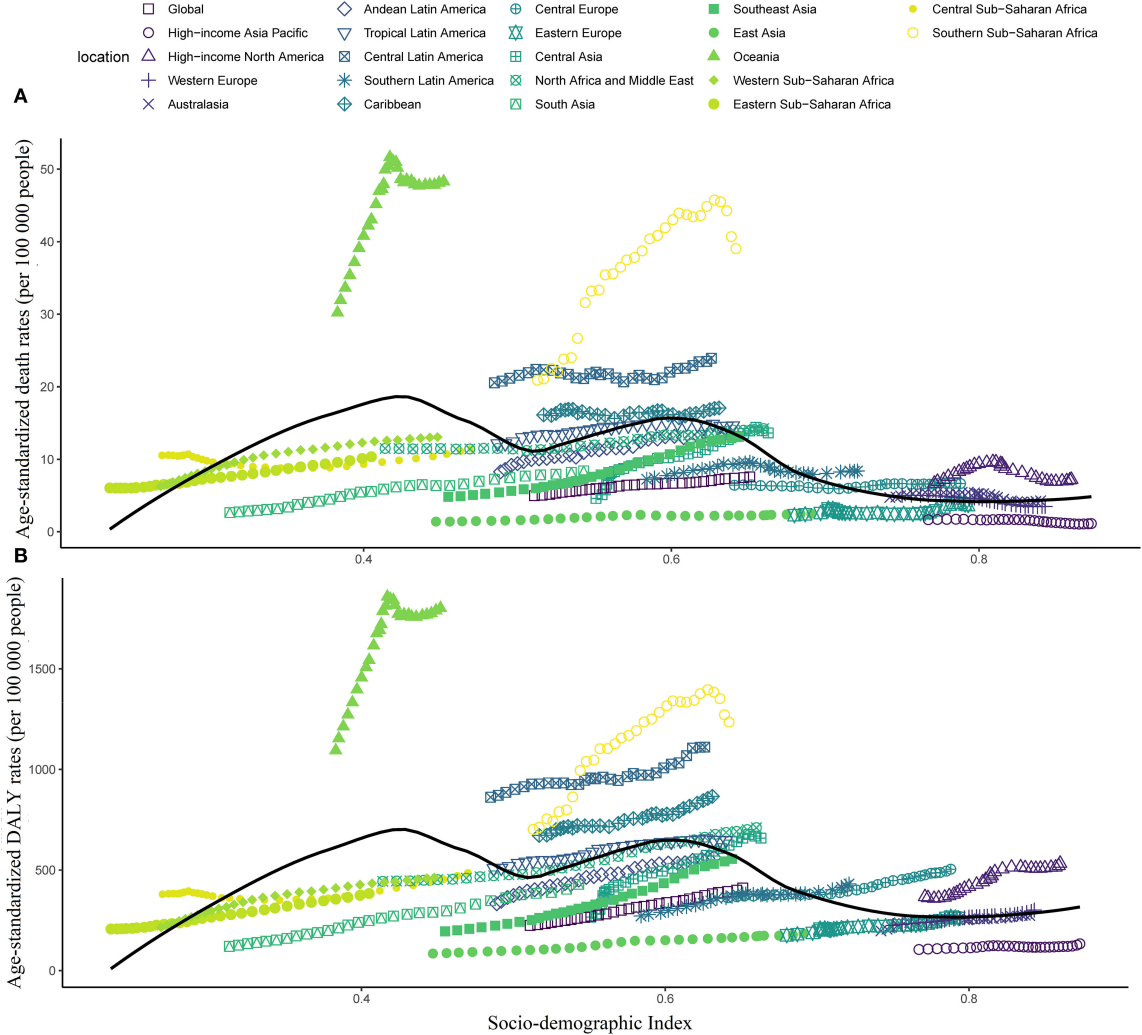
Age-standardized mortality rate (ASMR) and age-standardized disability-adjusted life year rate (ASDR) attributable to high body mass index across 21 GBD regions by the socio-demographic index for both sexes combined, 1990–2019. **(A)** ASMR; and **(B)** ASDR.

Across the 21 GBD regions, the highest ASMR and ASDR of high BMI-related were observed in Oceania (48.3 and 1,801.3 per 100,000 populations), followed by Southern Sub-Saharan Africa (39.1 and 1,235.3 per 100,000 populations) and Central Latin America (23.9 and 1,110.7 per 100,000 populations) in 2019 (Figure 3), whereas the region with the lowest ASMR and ASDR was high-income Asia Pacific (1.1 and 133.4 per 100,000 populations), followed by East Asia (2.5 and 184.3 per 100,000 populations), and Western Europe (3.5 and 260.9 per 100,000 populations) (Tables 1, 2). From 1990 to 2019, the EAPCs of ASMR in Australasia, high-income Asia Pacific, and Western Europe were −0.83, −1.86, and −1.54, respectively, which means the ASMR of T2D attributed to high BMI in the above three regions declined in the past 30 years. However, ASDR had substantially increased in all 21 GBD regions.

Interestingly, no decrease in high BMI-related T2D burden was observed with increasing SDI (Figure 3). Furthermore, the ASMR and ASDR were slightly increased from 1990 to 2019 in most GBD regions, regardless of the SDI values.

### T2D burden attributable to high BMI by SDI regions

**Figure 4:**
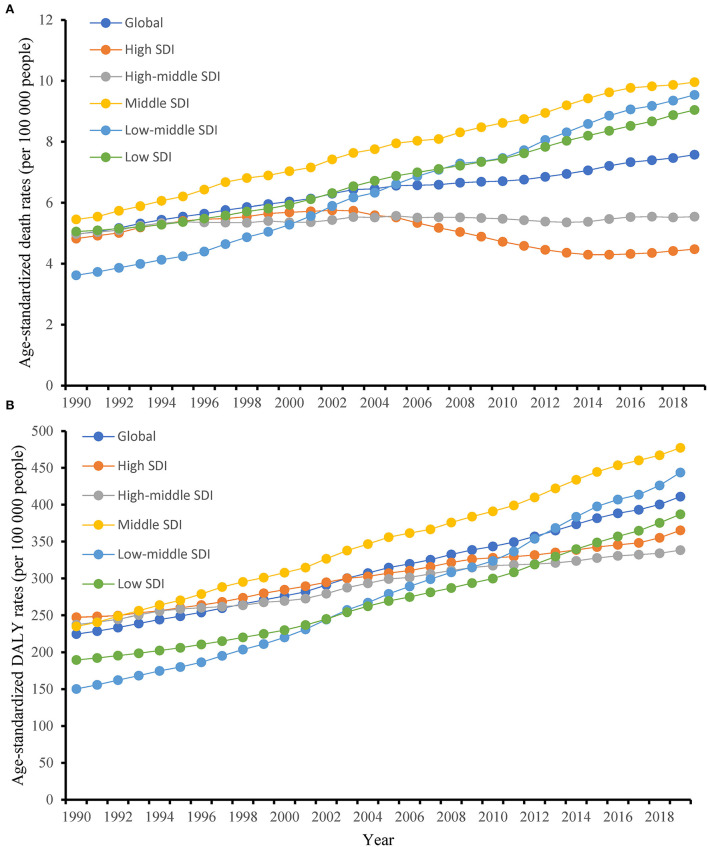
Changes in the age-standardized mortality rate (ASMR) and age-standardized disability-adjusted life year rate (ASDR) of type 2 diabetes attributable to high body mass index globally and in different socio-demographic index regions from 1990 to 2019. **(A)** ASMR; and **(B)** ASDR.

In 2019, ASMR was the highest in the middle SDI regions and the lowest in the high SDI regions, compared with the five SDI levels (Figure 4A). From 1990 to 2019, the ASMR in high SDI regions showed a slight decrease with no statistical significance (−7.2%), whereas low-middle SDI regions had the biggest increase in ASMR (1,632.4%). All SDI regions showed an obvious increase in absolute deaths of T2D attributed to high BMI between 1990 and 2019. Moreover, the ASMR in the middle SDI regions was the highest lasting 30 years.

ASDR was the highest in the middle SDI regions and the lowest in the high-middle SDI regions in 2019 (Figure 4B). All SDI regions showed a substantial increase in ASDR from 1990 to 2019, with low-middle SDI regions having the biggest increase (1,954.40%). Also, the absolute DALYs increased within all SDI regions between 1990 and 2019.

### T2D burden attributable to high BMI by ages and genders

**Figure 5:**
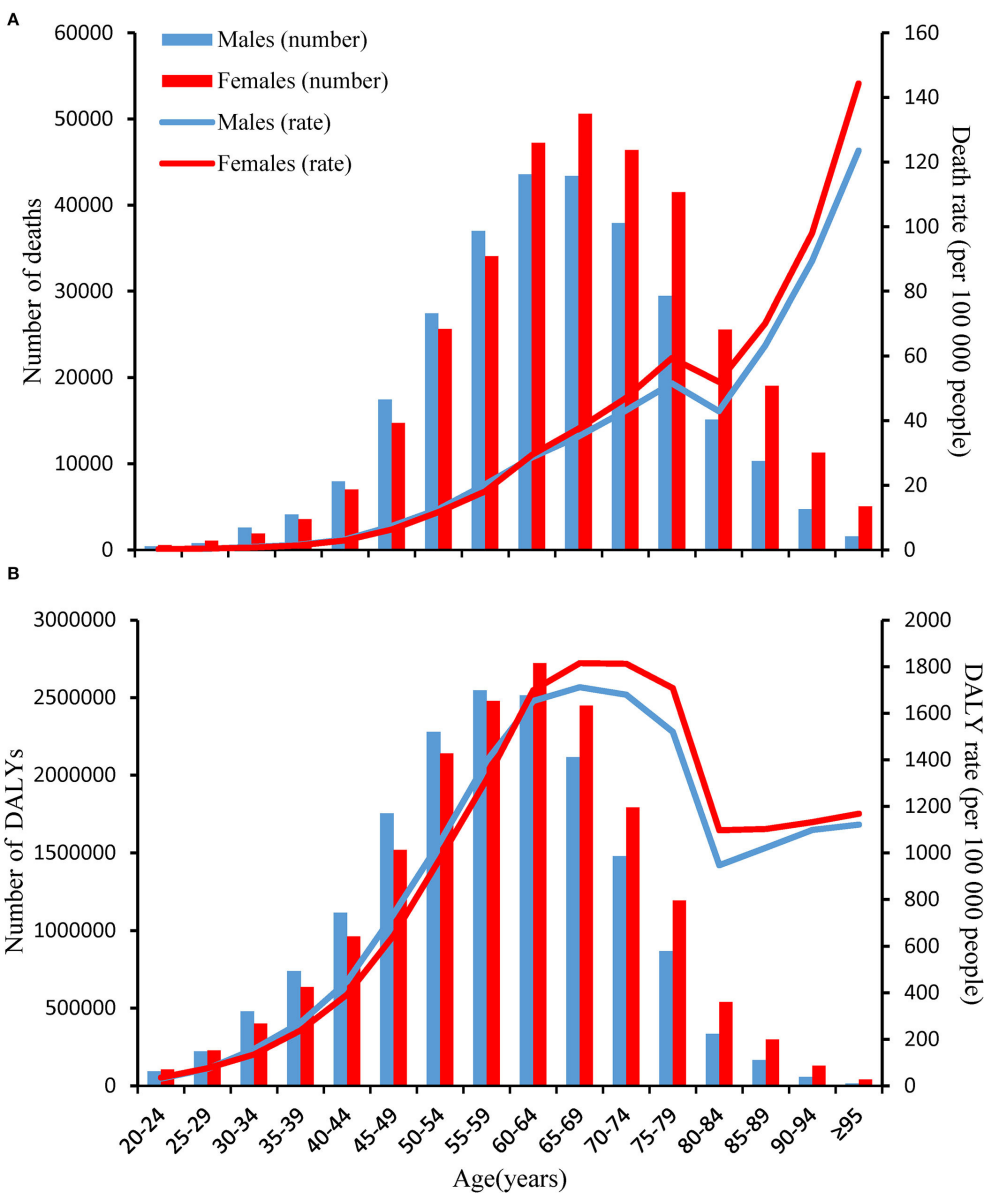
Age-specific numbers and rates of deaths and disability-adjusted life years (DALYs) of type 2 diabetes attributable to high body mass index by age and sex, in 2019. **(A)** Deaths; and **(B)** DALYs.

In 2019, age-specific rates of high BMI-related deaths and DALYs increased with increasing age globally, with the exception of death in 75–84 years and DALYs in aged 65–84 years. The number of T2D deaths peaked in the 65–69 years age group in women and the 60–64 years age group in men. Besides, the number of DALYs peaked in aged 60–64 years in women and aged 55–59 years in men (Figure 5). Furthermore, both the number and age-standardized rate in death and DALYs were lower in women than in men in age groups <60 years, whereas higher in women than in men in age groups≥60 years. The attributable proportions of T2D deaths and DALYs due to high BMI all peaked in the 40–45 years group globally (84.5 and 85.4%, separately) and then decreased with the increased age group. Among them, Eastern Sub-Saharan Africa had the biggest attributable proportions of T2D death (91.5%) and DALYs (91.6%) in aged 40–45 years (Supplementary Figure S1).

In addition, the ASMR of women was higher than men in 1990 (5.6 vs. 4.2 per 100,000 populations) and 2019 (7.7 vs. 7.4 per 100,000 populations) globally (Supplementary Table S4). The ASDR of women was more than men in 1990 (245.0 vs. 201.0 per 100,000 populations), whereas the ASDR of males was higher than women in 2019 (413.4 vs. 407.6 per 100,000 populations) (Supplementary Table S5). The global burden of T2D due to high BMI was substantially increased in both men and women between 1990 and 2019, while more pronounced in men.

## Discussion

This study combined 204 countries and territories and assessed the global patterns and trends of high BMI-related T2D burden spanning 30 years. Across the world, there were 619,494.8 deaths and 34,422,224.8 DALYs of T2D attributed to high BMI in 2019, more than triple in 1990, whereas the ratio of the population was 1.46 in 2019 compared with 1990 the whole world, highlighting a progressively greater burden of high BMI-related T2D. The marked increase in the absolute deaths and DALYs may be partially explained by the aging and growth of the population (Supplementary Table S6). Moreover, substantial increases were also observed in the ASMR and ASDR of 52.0% and 82.9% between 1990 and 2019, respectively. From an overall aspect, women are more susceptible than men (25), but the global T2D burden increased more obviously in men. In addition, both the number and age-standardized rate in deaths and DALYs were lower in women than in men in age groups <60 years, whereas higher in women than in men in age groups ≥60 years. As mentioned in the literature review, the global burden of T2D attributable to high BMI has been simply described (10), while this study took BMI as the primary object, which is more comprehensive. Overall, more importantly, a successful alarm is sounded for us that the high BMI in T2D needs to be properly and timely addressed.

From 1999–2000 to 2017–2018, the prevalence of obesity increased from 30.5 to 42.4% in the United States according to the Centers for Disease Control and Prevention (CDC) (26). Not alone, the prevalence of T2D changed from 3,545.6 to 5,282.9 per 100,000 populations globally. As their incidence increased, obesity and diabetes were recognized as an epidemic by the World Health Organization (WHO) (27). It is acknowledged that overweight and obesity have been proven to be risk factors for T2D, and they can reduce insulin sensitivity to peripheral tissues and cause β-cell dysfunction through numerous pathogenetic mechanisms, playing a considerable role in the initiation and development of T2D (28, 29). A previous study reported that there were more than one million deaths due to T2D in 2017, half of which were related to high BMI (11). A large and growing body of literature has investigated the relationship between T2D and obesity. Compared with people with normal weight, male subjects who were obese had a seven-fold higher risk while female subjects has a 12-fold higher risk of developing T2D (30). The other way round, patients with T2D were mostly obese, for example, 50.9 to 98.6% in Europe and 56.1 to 69.2% in Asia (27). Prior to the work of Abbasi, the prevalence of T2D increased from 6.4 persons to 33.2 per 100 000 populations from 1994–1998 to 1994–1998 (31). The above may be the cause of the high BMI-related T2D diabetes burden increase.

The evidence from this study suggested that the high BMI-related T2D burden was increased among women older than 60 years old compared with men. An alternative interpretation of the origins of this phenomenon may be associated with higher life expectancy in women (32). Another possible explanation for this might be the increased prevalence of sarcopenic obesity, which is characterized by the loss of muscle mass and strength, especially in the elderly (33). A moderate-intensity exercise and progressive elastic band resistance exercise are effective approaches to reducing fat mass in older women, which can be considered for target populations (34, 35).

Overweight and obesity are due to the fact that the body produces more nutrients than it needs. Generally speaking, lifestyle and diet are the most important contributors to obesity, in particular lower physical activity, high calorie/carbohydrate soft drinks, fast food consumption, and fewer fruits and vegetables (36). In our study, the lowest ASMR and ASDR of high BMI-related T2D were found in Japan in 2019. The reason for this phenomenon may be partially explained by its good eating habits, such as low intake of red meat, high intake of fish and plant foods, and non-sugar-sweetened beverages, which is worth learning by many other countries (37). However, Fiji had the highest ASMR and ASDR of high BMI-related T2D in 2019. Prior studies were found in the literature on the question of this phenomenon. An important finding was that sugar and processed food imports were the main reason increasing in average BMI in Fiji (38). Other studies considered that Fiji's socio-cultural, economic, political, and physical environment led to the lack of knowledge and understanding of healthy eating among local people, and the inability to buy high-quality healthy food, often overeating processed food, so the prevalence of obesity is very high, resulting in a high BMI situation (39). Considerably, more work at both the individual and policymaker levels will need to be done to reduce the impact of high BMI on T2D in the near future.

At the GBD region level, the ASMR of T2D attributable to high BMI decreased only in Australasia, high-income Asia Pacific, and Western Europe, whereas the ASDR increased in all 21 GBD regions over the past 30 years. As mentioned in the result section, the top three regions with the highest burdens were Oceania, Southern Sub-Saharan Africa, and Central Latin America. According to a survey, Oceania is one of the main regions for red and processed meat import, which can contribute to a substantial rise in the burden of type 2 diabetes and obesity (40). Moreover, at least 72% of adults have less fruit and vegetable intake daily than the recommended quantity, and 31–75% have little physical activity in Southern Africa (41). All the above factors can give birth to a negative effect on the high BMI-related T2D burden. High-income Asia Pacific countries mainly include Japan, Singapore, Brunei Darussalam, and the Republic of Korea in the GBD Study, which experienced the lowest burdens in 2019. Among them, Singapore shared key obesity prevention and management strategies including health-promoting public policies, shaping healthier habits, mobilizing a social movement, and clinical management of high BMI (42). The T2D burden in South Asia was only higher than in high-income Asia Pacific countries. What is surprising is that the biggest increase in high BMI-related T2D burden between 1990 and 2019 was also found in South Asia, which mostly belongs to the low-middle SDI regions. Although the prevalence of obesity is not high in these locations (43), a lower social development level and lower medical level may accelerate the high BMI-related T2D burden.

Differences in age structure may lead to the heterogeneity of the T2D burden attributable to high BMI (44). Therefore, ASMR and ASDR were used to estimate the high BMI-related T2D burden to eliminate the influence of age structure differences. Besides, the all-age number of deaths or DALYs did not take into account the impact of age structure on outcomes. When given inconsistent results, ASMR and ASDR were the most appropriate metrics to use.

Although covered 204 countries and territories, several limitations need discussion briefly. First, the GBD estimation of the T2D burden attributable to high BMI may deviate from the actual data to some degree due to the use of a large number of data sources of different quality. Second, the comorbidities of T2D could not be taken into account when estimating the burden of diabetes. It can affect the T2D burden. Third, there was a lack of data for some countries; thus, the burden of T2D attributable to high BMI in those countries was estimated using the GBD modeling process. Fourth, the uncertainty interval for age-standardized deaths rate was relatively wide in low-SDI regions, which was due to the lack of adequate population-based data, and such results required careful interpretation.

## Conclusion

In summary, high BMI is an important contributor to the T2D burden. From 1990 to 2019, the standardized and absolute T2D burden attributable to high BMI substantially increased globally, especially in men, in South Asia, and in low-middle SDI regions. Our findings will better guide the government to take tailored actions for the future management of T2D and high BMI in different regions.

## Data availability statement

The data used for these analyses are all publicly available at online GBD repository (http://ghdx.healthdata.org/gbd-results-tool).

## Author contributions

XZ and XW: investigation, software, data curation, formal analysis, writing the original draft, reviewing, and editing. MW, WT, YW, JG, and TN: investigation, formal analysis, writing the original draft, reviewing, and editing. BH and QL: conceptualization, software, and writing, reviewing, and editing. All authors contributed to the article and approved the submitted version.

## Conflict of interest

The authors declare that the research was conducted in the absence of any commercial or financial relationships that could be construed as a potential conflict of interest.

## Publisher's note

All claims expressed in this article are solely those of the authors and do not necessarily represent those of their affiliated organizations, or those of the publisher, the editors and the reviewers. Any product that may be evaluated in this article, or claim that may be made by its manufacturer, is not guaranteed or endorsed by the publisher.
