# Supplementary material for: The global burden of type 2 diabetes attributable to high body mass index in 204 countries and territories, 1990–2019: An analysis of the Global Burden of Disease Study
Source: Front Public Health. 2022 Sep 9;10:966093. doi: 10.3389/fpubh.2022.966093 (PMC9500174; doi:10.3389/fpubh.2022.966093)

Table S1. GATHER checklist of information that should be included in reports of global health estimates

| **#** | **Checklist item** | **Section/paragraph/** **interpretation** |
| --- | --- | --- |
| **Objectives and funding** | | |
| 1 | Define the indicators, populations, and time periods for which estimates were made. | Methods / “Data Sources” and “Definitions” section |
| 2 | List the funding sources for the work. | No funding |
| **Data Inputs** | | |
| *For all data inputs from multiple sources that are synthesized as part of the study:* | | |
| 3 | Describe how the data were identified and how the data were accessed. | As mentioned in the Methods / “Definitions” section, the details have been published previously. |
| 4 | Specify the inclusion and exclusion criteria. Identify all ad-hoc exclusions. | As mentioned in the Methods / “Definitions” section, the details have been published previously. |
| 5 | Provide information on all included data sources and their main characteristics. For each data source used, report reference information or contact name/institution, population represented, data collection method, year(s) of data collection, sex and age range, diagnostic criteria or measurement method, and sample size, as relevant. | Available via online data source tools (http://ghdx.healthdata.org/gbd-2019/data-input-sources). |
| 6 | Identify and describe any categories of input data that have potentially important biases (e.g., based on characteristics listed in item 5). | As mentioned in the Methods / “Definitions” section, the details have been published previously. |
| *For data inputs that contribute to the analysis but were not synthesized as part of the study:* | | |
| 7 | Describe and give sources for any other data inputs. | Available via online data source tools (http://ghdx.healthdata.org/gbd-2019/data-input-sources). |
| *For all data inputs:* | | |
| 8 | Provide all data inputs in a file format from which data can be efficiently extracted (e.g., a spreadsheet as opposed to a PDF), including all relevant meta-data listed in item 5. For any data inputs that cannot be shared due to ethical or legal reasons, such as third-party ownership, provide a contact name or the name of the institution that retains the right to the data. | Available via online data source tools (http://ghdx.healthdata.org/gbd-2019/data-input-sources); |
| **Data analysis** | | |
| 9 | Provide a conceptual overview of the data analysis method. A diagram may be helpful. | Flow diagrams of the overall methodological processes were available online  (http://ghdx.healthdata.org/gbd-2019/code) |
| 10 | Provide a detailed description of all steps of the analysis, including mathematical formulae. This description should cover, as relevant, data cleaning, data pre-processing, data adjustments and weighting of data sources, and mathematical or statistical model(s). | As mentioned in the Methods / “Statistical Analysis” section, the details have been published previously. |
| 11 | Describe how candidate models were evaluated and how the final model(s) were selected. | As mentioned in the Methods / “Statistical Analysis” section, the details have been published previously. |
| 12 | Provide the results of an evaluation of model performance, if done, as well as the results of any relevant sensitivity analysis. | As mentioned in the Methods / “Statistical Analysis” section, the details have been published previously. |
| 13 | Describe methods for calculating uncertainty of the estimates. State which sources of uncertainty were, and were not, accounted for in the uncertainty analysis. | Methods / “Statistical Analysis” section |
| 14 | State how analytic or statistical source code used to generate estimates can be accessed. | Methods / “Statistical Analysis” section |
| **Results and Discussion** | | |
| 15 | Provide published estimates in a file format from which data can be efficiently extracted. | Results, and online data tools (data visualization tools, and data query tools,  http://ghdx.healthdata.org/gbd-2019) |
| 16 | Report a quantitative measure of the uncertainty of the estimates (e.g. uncertainty intervals). | Results, and online data tools (data visualization tools, and data query tools,  http://ghdx.healthdata.org/gbd-2019) |
| 17 | Interpret results in light of existing evidence. If updating a previous set of estimates, describe the reasons for changes in estimates. | Discussion |
| 18 | Discuss limitations of the estimates. Include a discussion of any modelling assumptions or data limitations that affect interpretation of the estimates. | Discussion |

Table S2. T2D deaths attributable to high BMI in 204 countries and territories between 1990 and 2019.

| 204 countries and territories | Death case | | Percentage change in number of  Deaths,1990-2019 | ASMR per 100 000 people | | Percentage change in  ASMR,1990-2019 |
| --- | --- | --- | --- | --- | --- | --- |
|  | 1990 | 2019 |  | 1990 | 2019 |  |
| Afghanistan | 704.0 ( 322.5 , 1249.1 ) | 2475.1 ( 1287.6 , 4015.4 ) | 251.6 ( 131.2 , 462.9 ) | 9.4 ( 4.3 , 16.7 ) | 20.1 ( 10.4 , 32.8 ) | 113.5 ( 44.0 , 243.3 ) |
| Albania | 34.1 ( 21.8 , 46.8 ) | 75.2 ( 45.5 , 114.3 ) | 120.3 ( 55.3 , 207.8 ) | 1.7 ( 1.1 , 2.4 ) | 1.7 ( 1.0 , 2.6 ) | -1.8 ( -29.4 , 38.1 ) |
| Algeria | 729.9 ( 435.9 , 1166.6 ) | 2890.0 ( 1962.7 , 4071.1 ) | 295.9 ( 181.8 , 471.7 ) | 6.4 ( 3.7 , 10.6 ) | 9.5 ( 6.3 , 13.6 ) | 47.7 ( 7.1 , 110.7 ) |
| American Samoa | 10.9 ( 8.2 , 13.6 ) | 29.0 ( 21.6 , 36.7 ) | 165.1 ( 113.2 , 224.2 ) | 47.8 ( 34.4 , 62.0 ) | 60.1 ( 43.6 , 77.4 ) | 25.8 ( 1.9 , 53.8 ) |
| Andorra | 1.4 ( 0.8 , 2.3 ) | 3.5 ( 2.1 , 5.3 ) | 148.7 ( 66.7 , 266.7 ) | 2.8 ( 1.6 , 4.6 ) | 2.4 ( 1.4 , 3.5 ) | -16.7 ( -43.2 , 19.9 ) |
| Angola | 188.0 ( 41.7 , 424.6 ) | 1375.1 ( 731.8 , 2120.8 ) | 631.6 ( 280.2 , 2228.1 ) | 4.6 ( 1.0 , 10.6 ) | 11.8 ( 6.2 , 18.9 ) | 159.2 ( 37.8 , 706.9 ) |
| Antigua and Barbuda | 10.0 ( 5.6 , 14.9 ) | 25.7 ( 17.4 , 35.1 ) | 157.6 ( 101.6 , 265.3 ) | 19.4 ( 11.3 , 28.5 ) | 25.7 ( 17.0 , 35.8 ) | 32.9 ( 5.5 , 81.4 ) |
| Argentina | 2420.6 ( 1387.6 , 3500.0 ) | 4878.4 ( 3212.6 , 6587.2 ) | 101.5 ( 70.1 , 168.9 ) | 7.5 ( 4.3 , 10.9 ) | 9.0 ( 6.0 , 12.1 ) | 19.9 ( 0.9 , 61.0 ) |
| Armenia | 229.6 ( 161.3 , 301.8 ) | 702.5 ( 501.7 , 913.6 ) | 206.0 ( 146.0 , 285.3 ) | 8.3 ( 5.8 , 11.2 ) | 16.8 ( 11.9 , 21.9 ) | 102.0 ( 61.0 , 158.0 ) |
| Australia | 925.4 ( 603.0 , 1265.8 ) | 1917.1 ( 1255.3 , 2672.0 ) | 107.2 ( 74.0 , 149.6 ) | 4.8 ( 3.1 , 6.6 ) | 4.3 ( 2.9 , 5.9 ) | -8.9 ( -20.9 , 8.5 ) |
| Austria | 559.3 ( 343.8 , 802.2 ) | 875.2 ( 506.9 , 1293.6 ) | 56.5 ( 31.7 , 86.6 ) | 4.6 ( 2.8 , 6.6 ) | 4.5 ( 2.7 , 6.4 ) | -2.1 ( -16.0 , 15.6 ) |
| Azerbaijan | 284.6 ( 196.3 , 372.5 ) | 1268.2 ( 891.2 , 1663.9 ) | 345.6 ( 236.5 , 479.6 ) | 5.5 ( 3.7 , 7.2 ) | 13.4 ( 9.2 , 18.1 ) | 145.6 ( 84.9 , 219.2 ) |
| Bahamas | 37.2 ( 26.5 , 48.6 ) | 79.2 ( 54.5 , 106.6 ) | 112.7 ( 69.7 , 168.2 ) | 24.3 ( 17.0 , 32.1 ) | 20.4 ( 13.7 , 27.8 ) | -16.0 ( -32.4 , 6.2 ) |
| Bahrain | 59.1 ( 42.1 , 77.5 ) | 474.3 ( 334.0 , 630.3 ) | 702.5 ( 491.0 , 963.5 ) | 39.6 ( 26.6 , 53.6 ) | 69.5 ( 47.0 , 95.4 ) | 75.4 ( 30.0 , 124.8 ) |
| Bangladesh | 699.0 ( 121.4 , 1773.8 ) | 5797.4 ( 2821.6 , 9707.6 ) | 729.4 ( 363.5 , 2649.7 ) | 1.5 ( 0.3 , 3.9 ) | 4.6 ( 2.1 , 8.0 ) | 204.2 ( 72.8 , 897.5 ) |
| Barbados | 90.4 ( 56.4 , 125.4 ) | 145.3 ( 96.4 , 200.4 ) | 60.6 ( 30.6 , 103.8 ) | 31.0 ( 19.7 , 42.3 ) | 29.5 ( 19.7 , 40.7 ) | -4.6 ( -22.4 , 19.8 ) |
| Belarus | 289.6 ( 205.7 , 375.1 ) | 201.0 ( 135.9 , 278.5 ) | -30.6 ( -46.7 , -8.0 ) | 2.2 ( 1.6 , 2.9 ) | 1.3 ( 0.9 , 1.8 ) | -42.8 ( -55.7 , -24.1 ) |
| Belgium | 601.7 ( 367.2 , 871.0 ) | 616.5 ( 369.6 , 908.1 ) | 2.5 ( -14.6 , 26.2 ) | 3.9 ( 2.4 , 5.6 ) | 2.5 ( 1.6 , 3.5 ) | -36.5 ( -45.0 , -23.4 ) |
| Belize | 16.0 ( 9.3 , 23.2 ) | 90.9 ( 66.8 , 116.6 ) | 468.7 ( 343.3 , 701.0 ) | 17.3 ( 10.0 , 25.2 ) | 32.9 ( 23.6 , 42.9 ) | 89.9 ( 49.4 , 164.6 ) |
| Benin | 119.8 ( 58.0 , 194.7 ) | 662.5 ( 427.1 , 967.2 ) | 452.8 ( 275.1 , 872.4 ) | 6.0 ( 2.9 , 10.0 ) | 13.7 ( 8.6 , 20.3 ) | 127.4 ( 59.1 , 292.8 ) |
| Bermuda | 9.8 ( 6.7 , 12.8 ) | 12.1 ( 8.2 , 17.1 ) | 24.0 ( 0.1 , 53.9 ) | 16.0 ( 10.9 , 21.3 ) | 9.2 ( 6.3 , 12.7 ) | -42.6 ( -52.7 , -28.3 ) |
| Bhutan | 7.3 ( 2.3 , 14.6 ) | 48.7 ( 26.3 , 77.5 ) | 568.8 ( 326.0 , 1275.0 ) | 2.9 ( 0.9 , 6.0 ) | 9.0 ( 4.6 , 14.4 ) | 205.0 ( 94.1 , 542.1 ) |
| Bolivia (Plurinational State of) | 509.8 ( 307.2 , 756.3 ) | 2065.1 ( 1358.0 , 2889.6 ) | 305.1 ( 194.5 , 482.6 ) | 15.4 ( 9.1 , 23.1 ) | 23.5 ( 15.4 , 33.3 ) | 52.5 ( 11.1 , 119.4 ) |
| Bosnia and Herzegovina | 238.0 ( 157.4 , 318.3 ) | 1129.3 ( 747.9 , 1610.3 ) | 374.5 ( 229.7 , 540.2 ) | 6.2 ( 4.0 , 8.4 ) | 18.7 ( 12.4 , 26.7 ) | 200.9 ( 108.3 , 306.7 ) |
| Botswana | 103.7 ( 54.6 , 169.1 ) | 647.3 ( 419.1 , 895.0 ) | 524.1 ( 299.2 , 1013.8 ) | 18.2 ( 9.6 , 29.8 ) | 50.7 ( 32.8 , 70.5 ) | 178.0 ( 79.4 , 398.5 ) |
| Brazil | 10862.5 ( 6997.5 , 15055.0 ) | 33811.0 ( 24964.7 , 43247.8 ) | 211.3 ( 169.2 , 285.7 ) | 12.2 ( 7.7 , 17.3 ) | 14.5 ( 10.5 , 18.6 ) | 18.1 ( 1.8 , 49.2 ) |
| Brunei Darussalam | 18.0 ( 5.7 , 34.2 ) | 63.1 ( 36.7 , 92.7 ) | 251.1 ( 138.3 , 600.4 ) | 19.6 ( 6.2 , 38.5 ) | 22.4 ( 12.4 , 33.7 ) | 13.9 ( -20.7 , 122.4 ) |
| Bulgaria | 1121.6 ( 802.2 , 1412.4 ) | 1138.0 ( 759.0 , 1593.2 ) | 1.5 ( -20.4 , 28.2 ) | 8.7 ( 6.2 , 10.9 ) | 7.7 ( 5.2 , 10.7 ) | -11.1 ( -29.7 , 11.8 ) |
| Burkina Faso | 196.3 ( 58.4 , 396.4 ) | 956.7 ( 539.3 , 1430.2 ) | 387.4 ( 217.1 , 953.1 ) | 4.5 ( 1.3 , 9.4 ) | 10.5 ( 5.8 , 16.2 ) | 130.6 ( 52.8 , 395.0 ) |
| Burundi | 150.5 ( 49.5 , 307.7 ) | 308.4 ( 133.0 , 541.6 ) | 104.8 ( 43.7 , 241.7 ) | 6.3 ( 2.0 , 12.9 ) | 6.6 ( 2.7 , 11.9 ) | 5.4 ( -24.4 , 71.8 ) |
| Cabo Verde | 4.7 ( 2.2 , 7.8 ) | 53.9 ( 36.5 , 74.6 ) | 1045.0 ( 643.2 , 1986.1 ) | 2.1 ( 1.0 , 3.5 ) | 13.1 ( 8.9 , 18.0 ) | 520.5 ( 309.1 , 1013.2 ) |
| Cambodia | 158.9 ( 39.6 , 365.0 ) | 894.2 ( 447.0 , 1489.3 ) | 462.7 ( 248.7 , 1274.9 ) | 3.3 ( 0.8 , 7.6 ) | 7.3 ( 3.6 , 12.4 ) | 123.4 ( 40.8 , 445.2 ) |
| Cameroon | 594.5 ( 364.9 , 868.0 ) | 2831.2 ( 1865.0 , 4069.1 ) | 376.2 ( 235.1 , 600.1 ) | 13.8 ( 8.2 , 20.5 ) | 25.1 ( 16.3 , 36.4 ) | 82.2 ( 30.1 , 164.7 ) |
| Canada | 1580.8 ( 1017.8 , 2180.0 ) | 3230.1 ( 2159.8 , 4430.2 ) | 104.3 ( 76.0 , 143.3 ) | 4.9 ( 3.1 , 6.7 ) | 4.5 ( 3.1 , 6.0 ) | -7.9 ( -20.1 , 10.3 ) |
| Central African Republic | 87.1 ( 28.7 , 176.8 ) | 237.1 ( 97.1 , 421.9 ) | 172.1 ( 89.1 , 395.1 ) | 6.8 ( 2.2 , 14.1 ) | 9.9 ( 4.0 , 18.1 ) | 45.4 ( 2.9 , 160.0 ) |
| Chad | 78.5 ( 24.1 , 158.7 ) | 455.8 ( 233.3 , 729.4 ) | 480.5 ( 282.2 , 1123.4 ) | 2.8 ( 0.8 , 5.7 ) | 8.0 ( 4.1 , 13.0 ) | 186.9 ( 92.7 , 492.3 ) |
| Chile | 671.6 ( 429.4 , 909.6 ) | 1799.3 ( 1178.4 , 2445.1 ) | 167.9 ( 130.2 , 216.9 ) | 6.9 ( 4.3 , 9.3 ) | 7.5 ( 4.9 , 10.2 ) | 9.0 ( -5.7 , 29.1 ) |
| China | 10510.4 ( 2937.8 , 21514.0 ) | 47529.6 ( 22514.2 , 76631.9 ) | 352.2 ( 213.6 , 798.1 ) | 1.2 ( 0.3 , 2.6 ) | 2.4 ( 1.1 , 3.9 ) | 91.2 ( 32.8 , 283.6 ) |
| Colombia | 1322.2 ( 846.4 , 1817.3 ) | 3275.3 ( 2109.8 , 4803.7 ) | 147.7 ( 83.2 , 238.8 ) | 7.5 ( 4.7 , 10.6 ) | 6.2 ( 4.0 , 9.0 ) | -18.1 ( -39.2 , 13.0 ) |
| Comoros | 17.5 ( 6.6 , 31.3 ) | 52.4 ( 29.9 , 79.4 ) | 198.7 ( 101.0 , 507.3 ) | 7.9 ( 3.0 , 14.2 ) | 10.9 ( 6.0 , 16.5 ) | 37.1 ( -6.0 , 168.8 ) |
| Congo | 181.9 ( 91.7 , 294.3 ) | 588.9 ( 390.0 , 831.6 ) | 223.8 ( 119.7 , 450.5 ) | 16.2 ( 7.9 , 26.8 ) | 22.4 ( 14.5 , 31.9 ) | 37.9 ( -4.3 , 130.3 ) |
| Cook Islands | 8.3 ( 5.9 , 11.0 ) | 16.1 ( 11.4 , 21.1 ) | 92.8 ( 49.8 , 147.0 ) | 65.6 ( 45.3 , 89.0 ) | 65.4 ( 46.0 , 86.3 ) | -0.4 ( -21.9 , 28.7 ) |
| Costa Rica | 119.0 ( 79.6 , 160.3 ) | 275.8 ( 176.7 , 404.1 ) | 131.7 ( 73.5 , 214.3 ) | 6.8 ( 4.5 , 9.3 ) | 5.4 ( 3.4 , 7.9 ) | -21.3 ( -41.3 , 7.0 ) |
| Croatia | 363.6 ( 249.4 , 477.9 ) | 545.1 ( 353.6 , 781.1 ) | 49.9 ( 14.6 , 93.3 ) | 5.8 ( 3.9 , 7.7 ) | 5.9 ( 3.9 , 8.3 ) | 1.3 ( -21.6 , 29.4 ) |
| Cuba | 964.7 ( 674.4 , 1257.2 ) | 954.6 ( 635.1 , 1317.7 ) | -1.0 ( -21.9 , 23.7 ) | 9.5 ( 6.6 , 12.3 ) | 5.0 ( 3.4 , 6.9 ) | -47.1 ( -58.2 , -33.2 ) |
| Cyprus | 119.3 ( 64.3 , 185.6 ) | 154.1 ( 87.9 , 233.4 ) | 29.2 ( 0.4 , 78.9 ) | 15.6 ( 7.9 , 25.4 ) | 8.3 ( 4.7 , 12.9 ) | -46.9 ( -58.9 , -24.0 ) |
| Czechia | 855.3 ( 583.7 , 1119.3 ) | 1696.7 ( 1127.7 , 2364.6 ) | 98.4 ( 55.5 , 145.0 ) | 6.1 ( 4.2 , 8.1 ) | 7.6 ( 5.1 , 10.6 ) | 24.0 ( -2.8 , 55.2 ) |
| C么te d'Ivoire | 355.0 ( 198.3 , 549.4 ) | 1471.0 ( 916.6 , 2120.6 ) | 314.3 ( 185.8 , 527.2 ) | 8.7 ( 4.6 , 13.8 ) | 13.9 ( 8.3 , 20.4 ) | 60.9 ( 15.5 , 140.6 ) |
| Democratic People's Republic of Korea | 290.1 ( 60.3 , 657.3 ) | 559.4 ( 130.0 , 1246.1 ) | 92.8 ( 41.2 , 174.0 ) | 1.8 ( 0.4 , 4.0 ) | 1.7 ( 0.4 , 3.8 ) | -2.8 ( -28.6 , 37.4 ) |
| Democratic Republic of the Congo | 1881.0 ( 1040.7 , 2882.3 ) | 3566.9 ( 1851.8 , 5518.7 ) | 89.6 ( 36.0 , 166.0 ) | 11.8 ( 6.3 , 18.5 ) | 9.7 ( 4.9 , 15.5 ) | -17.2 ( -39.3 , 13.6 ) |
| Denmark | 279.0 ( 172.3 , 397.3 ) | 518.7 ( 326.3 , 738.2 ) | 85.9 ( 58.0 , 124.2 ) | 3.5 ( 2.2 , 5.0 ) | 4.4 ( 2.8 , 6.1 ) | 24.5 ( 7.0 , 49.0 ) |
| Djibouti | 5.2 ( 1.3 , 11.8 ) | 71.5 ( 37.8 , 117.7 ) | 1273.4 ( 671.5 , 3647.9 ) | 3.6 ( 0.9 , 8.4 ) | 12.0 ( 6.1 , 19.6 ) | 228.2 ( 89.3 , 786.2 ) |
| Dominica | 20.5 ( 13.4 , 28.6 ) | 28.7 ( 19.1 , 40.1 ) | 40.2 ( 10.3 , 85.9 ) | 28.9 ( 19.2 , 39.8 ) | 32.0 ( 21.3 , 44.4 ) | 10.6 ( -12.9 , 44.8 ) |
| Dominican Republic | 251.6 ( 130.5 , 385.1 ) | 1394.6 ( 888.0 , 2064.3 ) | 454.4 ( 281.7 , 784.0 ) | 6.5 ( 3.3 , 10.2 ) | 14.7 ( 9.2 , 22.0 ) | 125.5 ( 54.0 , 263.8 ) |
| Ecuador | 586.1 ( 421.5 , 742.9 ) | 2794.1 ( 1905.5 , 3834.4 ) | 376.7 ( 258.6 , 525.0 ) | 10.9 ( 7.7 , 14.1 ) | 18.9 ( 12.8 , 26.2 ) | 72.8 ( 30.4 , 128.3 ) |
| Egypt | 3422.7 ( 2449.1 , 4391.9 ) | 13420.2 ( 9265.3 , 18507.6 ) | 292.1 ( 186.6 , 444.2 ) | 11.8 ( 8.3 , 15.6 ) | 21.1 ( 14.4 , 29.3 ) | 79.0 ( 31.7 , 147.7 ) |
| El Salvador | 215.7 ( 135.7 , 300.3 ) | 1158.4 ( 736.0 , 1696.8 ) | 437.1 ( 274.5 , 659.1 ) | 7.2 ( 4.5 , 10.1 ) | 19.7 ( 12.6 , 28.7 ) | 173.3 ( 89.1 , 287.2 ) |
| Equatorial Guinea | 11.7 ( 3.1 , 25.5 ) | 119.9 ( 73.0 , 180.0 ) | 924.0 ( 380.8 , 3386.6 ) | 5.6 ( 1.4 , 12.5 ) | 27.0 ( 16.4 , 40.6 ) | 383.5 ( 133.7 , 1511.3 ) |
| Eritrea | 48.3 ( 16.3 , 99.7 ) | 325.8 ( 171.8 , 519.6 ) | 575.2 ( 314.2 , 1337.7 ) | 4.7 ( 1.6 , 9.9 ) | 12.9 ( 6.6 , 20.8 ) | 171.8 ( 65.3 , 480.1 ) |
| Estonia | 36.3 ( 25.8 , 48.0 ) | 72.8 ( 49.9 , 100.1 ) | 100.4 ( 51.0 , 159.2 ) | 1.8 ( 1.3 , 2.3 ) | 2.8 ( 2.0 , 3.8 ) | 57.9 ( 18.6 , 105.8 ) |
| Eswatini | 122.7 ( 82.7 , 166.2 ) | 456.6 ( 294.8 , 635.1 ) | 272.0 ( 166.1 , 437.3 ) | 45.2 ( 29.8 , 61.6 ) | 84.7 ( 53.3 , 118.4 ) | 87.3 ( 34.9 , 168.3 ) |
| Ethiopia | 1217.5 ( 300.5 , 2808.7 ) | 2866.2 ( 1408.8 , 4701.5 ) | 135.4 ( 39.4 , 477.0 ) | 5.7 ( 1.4 , 13.6 ) | 7.1 ( 3.3 , 12.1 ) | 24.5 ( -25.5 , 210.6 ) |
| Fiji | 305.6 ( 202.3 , 423.8 ) | 1144.4 ( 816.8 , 1533.0 ) | 274.5 ( 161.9 , 439.7 ) | 80.8 ( 50.7 , 117.3 ) | 152.1 ( 105.6 , 208.8 ) | 88.3 ( 33.7 , 170.5 ) |
| Finland | 176.5 ( 110.3 , 252.1 ) | 182.3 ( 115.1 , 259.9 ) | 3.3 ( -13.4 , 26.4 ) | 2.5 ( 1.6 , 3.5 ) | 1.5 ( 1.0 , 2.1 ) | -39.8 ( -48.8 , -26.7 ) |
| France | 2235.9 ( 1325.5 , 3310.7 ) | 4462.3 ( 2686.6 , 6627.4 ) | 99.6 ( 68.3 , 147.2 ) | 2.6 ( 1.6 , 3.8 ) | 2.9 ( 1.9 , 4.1 ) | 9.4 ( -5.3 , 31.6 ) |
| Gabon | 94.2 ( 45.8 , 159.3 ) | 323.2 ( 213.3 , 454.6 ) | 243.3 ( 118.9 , 511.2 ) | 16.4 ( 7.8 , 28.0 ) | 31.6 ( 20.4 , 44.7 ) | 92.9 ( 25.9 , 243.0 ) |
| Gambia | 18.4 ( 8.2 , 31.3 ) | 129.4 ( 81.1 , 189.1 ) | 602.9 ( 352.0 , 1206.2 ) | 5.3 ( 2.3 , 9.3 ) | 13.9 ( 8.5 , 20.5 ) | 162.0 ( 69.8 , 377.8 ) |
| Georgia | 340.9 ( 249.1 , 434.1 ) | 677.4 ( 471.6 , 893.2 ) | 98.7 ( 62.2 , 139.8 ) | 5.4 ( 3.9 , 6.9 ) | 11.3 ( 7.9 , 14.9 ) | 110.2 ( 71.0 , 153.3 ) |
| Germany | 8283.1 ( 5291.7 , 11646.6 ) | 8703.2 ( 5138.8 , 12691.2 ) | 5.1 ( -12.8 , 22.2 ) | 6.4 ( 4.1 , 8.8 ) | 4.2 ( 2.7 , 5.9 ) | -34.1 ( -43.3 , -24.9 ) |
| Ghana | 384.4 ( 174.1 , 658.3 ) | 3285.8 ( 2324.0 , 4373.1 ) | 754.8 ( 447.2 , 1583.8 ) | 5.9 ( 2.5 , 10.4 ) | 21.2 ( 15.0 , 28.8 ) | 259.3 ( 130.6 , 612.9 ) |
| Greece | 444.4 ( 274.2 , 631.8 ) | 555.5 ( 344.3 , 791.8 ) | 25.0 ( 8.5 , 45.5 ) | 2.9 ( 1.8 , 4.1 ) | 2.3 ( 1.5 , 3.1 ) | -21.1 ( -30.4 , -8.2 ) |
| Greenland | 2.5 ( 1.7 , 3.4 ) | 3.3 ( 2.2 , 4.5 ) | 29.7 ( -5.4 , 71.9 ) | 7.5 ( 5.0 , 10.2 ) | 4.8 ( 3.1 , 6.7 ) | -36.2 ( -52.8 , -15.5 ) |
| Grenada | 15.4 ( 8.6 , 23.2 ) | 36.0 ( 25.7 , 46.8 ) | 134.5 ( 88.1 , 239.7 ) | 22.4 ( 13.1 , 33.2 ) | 32.5 ( 22.7 , 43.0 ) | 44.7 ( 18.6 , 101.3 ) |
| Guam | 13.8 ( 9.1 , 18.4 ) | 26.9 ( 18.7 , 36.0 ) | 95.8 ( 55.1 , 167.4 ) | 18.9 ( 11.9 , 26.6 ) | 14.0 ( 9.6 , 18.8 ) | -26.2 ( -42.5 , 1.8 ) |
| Guatemala | 160.9 ( 71.7 , 263.6 ) | 2984.9 ( 1861.8 , 4394.3 ) | 1755.4 ( 1139.5 , 3070.2 ) | 4.2 ( 1.8 , 7.1 ) | 26.8 ( 16.6 , 39.7 ) | 539.6 ( 324.1 , 1016.7 ) |
| Guinea | 202.3 ( 95.8 , 343.8 ) | 671.0 ( 393.4 , 1010.4 ) | 231.7 ( 129.9 , 417.4 ) | 6.1 ( 2.8 , 10.5 ) | 12.2 ( 7.0 , 18.7 ) | 100.5 ( 40.4 , 210.8 ) |
| Guinea-Bissau | 31.7 ( 11.6 , 59.6 ) | 110.6 ( 63.2 , 173.0 ) | 249.2 ( 135.1 , 556.6 ) | 7.6 ( 2.7 , 14.6 ) | 14.9 ( 8.2 , 23.4 ) | 96.7 ( 35.5 , 258.2 ) |
| Guyana | 121.7 ( 74.9 , 171.9 ) | 299.9 ( 198.3 , 425.1 ) | 146.4 ( 80.0 , 256.0 ) | 30.9 ( 18.6 , 44.6 ) | 46.7 ( 30.4 , 66.7 ) | 51.1 ( 11.5 , 115.1 ) |
| Haiti | 588.3 ( 239.0 , 1015.9 ) | 1246.9 ( 605.3 , 2138.7 ) | 111.9 ( 42.9 , 257.2 ) | 16.9 ( 6.7 , 29.9 ) | 17.2 ( 8.2 , 29.8 ) | 1.6 ( -30.5 , 68.0 ) |
| Honduras | 88.5 ( 44.5 , 137.7 ) | 541.5 ( 348.2 , 790.8 ) | 512.0 ( 345.3 , 854.9 ) | 4.1 ( 2.1 , 6.4 ) | 8.9 ( 5.7 , 13.0 ) | 118.5 ( 60.9 , 239.6 ) |
| Hungary | 999.3 ( 744.0 , 1238.0 ) | 1339.9 ( 945.3 , 1816.5 ) | 34.1 ( 8.6 , 63.6 ) | 6.7 ( 5.0 , 8.3 ) | 6.8 ( 4.8 , 9.1 ) | 0.8 ( -17.5 , 23.3 ) |
| Iceland | 5.7 ( 3.4 , 8.2 ) | 10.1 ( 6.2 , 14.6 ) | 77.7 ( 49.5 , 109.4 ) | 1.9 ( 1.2 , 2.8 ) | 1.7 ( 1.1 , 2.4 ) | -11.9 ( -24.7 , 3.4 ) |
| India | 11460.5 ( 4876.8 , 20574.1 ) | 89811.7 ( 56965.7 , 126950.4 ) | 683.7 ( 434.0 , 1248.0 ) | 2.6 ( 1.1 , 4.9 ) | 8.1 ( 5.1 , 11.6 ) | 207.2 ( 108.2 , 447.1 ) |
| Indonesia | 5356.3 ( 1912.2 , 10005.4 ) | 43848.2 ( 29287.0 , 60662.8 ) | 718.6 ( 432.1 , 1616.2 ) | 4.7 ( 1.6 , 9.0 ) | 17.8 ( 11.5 , 25.1 ) | 277.4 ( 146.0 , 701.8 ) |
| Iran (Islamic Republic of) | 1112.5 ( 746.7 , 1514.0 ) | 7700.2 ( 5791.5 , 9655.6 ) | 592.2 ( 430.6 , 784.2 ) | 4.7 ( 3.0 , 6.6 ) | 11.4 ( 8.3 , 14.4 ) | 142.9 ( 84.2 , 217.3 ) |
| Iraq | 2204.6 ( 1471.8 , 2949.8 ) | 5789.3 ( 3964.9 , 7749.4 ) | 162.6 ( 93.0 , 257.9 ) | 28.5 ( 18.4 , 38.6 ) | 26.7 ( 18.3 , 35.7 ) | -6.2 ( -29.9 , 25.4 ) |
| Ireland | 157.1 ( 97.6 , 221.9 ) | 190.1 ( 115.7 , 277.7 ) | 21.0 ( -0.4 , 45.8 ) | 3.8 ( 2.4 , 5.4 ) | 2.5 ( 1.5 , 3.6 ) | -35.3 ( -45.2 , -23.0 ) |
| Israel | 329.5 ( 210.4 , 458.5 ) | 1064.3 ( 642.5 , 1533.7 ) | 223.0 ( 169.1 , 281.9 ) | 6.9 ( 4.4 , 9.7 ) | 8.8 ( 5.5 , 12.4 ) | 26.9 ( 10.1 , 45.9 ) |
| Italy | 6296.6 ( 3905.2 , 8973.2 ) | 7406.2 ( 4313.6 , 11111.0 ) | 17.6 ( -2.2 , 40.9 ) | 6.9 ( 4.2 , 9.8 ) | 4.6 ( 2.8 , 6.6 ) | -33.8 ( -41.2 , -23.6 ) |
| Jamaica | 458.3 ( 302.9 , 621.5 ) | 1169.0 ( 799.4 , 1633.3 ) | 155.1 ( 98.2 , 237.3 ) | 26.0 ( 17.5 , 35.1 ) | 38.7 ( 26.5 , 53.7 ) | 48.6 ( 15.9 , 95.0 ) |
| Japan | 2026.0 ( 763.0 , 3556.9 ) | 1510.4 ( 581.8 , 2761.6 ) | -25.4 ( -38.6 , -7.1 ) | 1.2 ( 0.5 , 2.1 ) | 0.4 ( 0.2 , 0.7 ) | -64.6 ( -67.8 , -57.9 ) |
| Jordan | 396.3 ( 276.2 , 518.2 ) | 1336.0 ( 985.1 , 1696.0 ) | 237.2 ( 165.2 , 347.9 ) | 33.4 ( 22.6 , 44.6 ) | 24.1 ( 17.0 , 31.9 ) | -27.9 ( -43.6 , -5.1 ) |
| Kazakhstan | 505.6 ( 377.6 , 630.7 ) | 1562.6 ( 1147.3 , 1977.1 ) | 209.0 ( 159.8 , 267.5 ) | 4.0 ( 3.0 , 5.0 ) | 9.4 ( 6.8 , 12.0 ) | 135.3 ( 99.2 , 179.8 ) |
| Kenya | 447.4 ( 213.3 , 737.2 ) | 2620.9 ( 1755.9 , 3667.8 ) | 485.7 ( 324.1 , 853.9 ) | 5.5 ( 2.5 , 9.3 ) | 11.9 ( 7.5 , 17.0 ) | 117.0 ( 59.6 , 247.6 ) |
| Kiribati | 31.1 ( 20.0 , 43.1 ) | 82.0 ( 51.4 , 114.1 ) | 163.7 ( 86.0 , 262.7 ) | 73.5 ( 46.4 , 103.8 ) | 101.7 ( 62.2 , 144.7 ) | 38.3 ( -1.1 , 88.9 ) |
| Kuwait | 80.1 ( 60.4 , 99.9 ) | 222.7 ( 160.0 , 294.8 ) | 178.2 ( 130.6 , 234.1 ) | 15.1 ( 10.7 , 20.1 ) | 10.6 ( 7.2 , 14.4 ) | -29.8 ( -41.8 , -14.3 ) |
| Kyrgyzstan | 93.9 ( 65.9 , 123.4 ) | 176.1 ( 127.5 , 231.4 ) | 87.6 ( 55.5 , 128.4 ) | 3.1 ( 2.1 , 4.0 ) | 3.7 ( 2.6 , 4.9 ) | 20.1 ( 0.5 , 45.5 ) |
| Lao People's Democratic Republic | 109.8 ( 31.9 , 238.6 ) | 552.6 ( 301.8 , 854.3 ) | 403.1 ( 176.1 , 1200.2 ) | 4.9 ( 1.4 , 10.8 ) | 11.9 ( 6.5 , 18.4 ) | 141.9 ( 35.6 , 520.0 ) |
| Latvia | 93.6 ( 67.4 , 120.6 ) | 181.0 ( 122.9 , 242.6 ) | 93.4 ( 53.5 , 141.7 ) | 2.6 ( 1.9 , 3.4 ) | 4.6 ( 3.2 , 6.1 ) | 75.0 ( 39.6 , 118.5 ) |
| Lebanon | 198.1 ( 132.1 , 272.2 ) | 448.9 ( 284.0 , 640.8 ) | 126.6 ( 58.3 , 207.6 ) | 9.1 ( 5.9 , 12.8 ) | 8.7 ( 5.5 , 12.4 ) | -4.6 ( -32.7 , 31.0 ) |
| Lesotho | 150.9 ( 85.2 , 234.9 ) | 702.1 ( 443.6 , 1022.5 ) | 365.4 ( 206.3 , 648.3 ) | 15.6 ( 8.6 , 24.5 ) | 57.3 ( 36.4 , 82.8 ) | 268.2 ( 147.2 , 488.3 ) |
| Liberia | 99.1 ( 56.7 , 149.8 ) | 291.2 ( 186.6 , 416.7 ) | 193.9 ( 96.5 , 368.2 ) | 8.9 ( 4.9 , 13.6 ) | 14.0 ( 8.8 , 20.4 ) | 57.5 ( 10.0 , 142.8 ) |
| Libya | 131.4 ( 86.4 , 182.9 ) | 544.4 ( 365.9 , 761.4 ) | 314.4 ( 184.7 , 505.5 ) | 7.3 ( 4.7 , 10.4 ) | 11.3 ( 7.4 , 15.9 ) | 54.5 ( 6.8 , 125.2 ) |
| Lithuania | 84.1 ( 57.8 , 111.2 ) | 125.6 ( 86.1 , 169.5 ) | 49.2 ( 17.9 , 85.7 ) | 1.9 ( 1.3 , 2.5 ) | 2.3 ( 1.6 , 3.0 ) | 20.7 ( -3.8 , 50.4 ) |
| Luxembourg | 21.2 ( 13.6 , 29.8 ) | 25.3 ( 15.8 , 37.7 ) | 19.5 ( -2.2 , 45.8 ) | 3.9 ( 2.5 , 5.5 ) | 2.4 ( 1.5 , 3.5 ) | -38.9 ( -48.6 , -26.1 ) |
| Madagascar | 253.5 ( 110.0 , 447.9 ) | 944.7 ( 511.5 , 1502.9 ) | 272.7 ( 143.6 , 542.9 ) | 4.8 ( 2.0 , 8.7 ) | 8.5 ( 4.4 , 13.7 ) | 74.6 ( 16.8 , 201.8 ) |
| Malawi | 221.9 ( 65.3 , 446.2 ) | 806.6 ( 439.3 , 1223.1 ) | 263.5 ( 133.2 , 700.3 ) | 5.7 ( 1.6 , 11.9 ) | 11.5 ( 6.1 , 17.8 ) | 102.1 ( 28.7 , 361.7 ) |
| Malaysia | 744.9 ( 419.0 , 1091.5 ) | 1657.6 ( 1098.9 , 2443.3 ) | 122.5 ( 55.8 , 241.3 ) | 7.9 ( 4.3 , 11.8 ) | 6.1 ( 4.0 , 9.2 ) | -21.7 ( -45.0 , 23.7 ) |
| Maldives | 3.9 ( 1.1 , 8.4 ) | 15.1 ( 8.8 , 22.3 ) | 283.8 ( 125.4 , 836.4 ) | 4.0 ( 1.0 , 8.9 ) | 4.9 ( 2.7 , 7.6 ) | 22.5 ( -26.8 , 201.7 ) |
| Mali | 201.8 ( 72.2 , 371.3 ) | 956.6 ( 554.0 , 1435.4 ) | 373.9 ( 214.8 , 800.6 ) | 4.9 ( 1.7 , 9.2 ) | 11.2 ( 6.3 , 17.2 ) | 129.9 ( 55.4 , 337.5 ) |
| Malta | 31.4 ( 18.8 , 45.9 ) | 44.0 ( 27.0 , 64.4 ) | 39.9 ( 15.1 , 76.3 ) | 7.5 ( 4.4 , 11.0 ) | 4.5 ( 2.8 , 6.6 ) | -39.3 ( -49.3 , -23.1 ) |
| Marshall Islands | 4.6 ( 2.2 , 7.4 ) | 19.3 ( 11.4 , 29.1 ) | 318.8 ( 193.2 , 565.8 ) | 27.1 ( 12.4 , 44.2 ) | 49.5 ( 27.7 , 76.4 ) | 82.8 ( 30.4 , 184.1 ) |
| Mauritania | 121.1 ( 74.8 , 178.2 ) | 319.6 ( 212.6 , 451.3 ) | 163.9 ( 87.8 , 282.9 ) | 12.3 ( 7.5 , 18.2 ) | 16.5 ( 10.8 , 23.4 ) | 33.9 ( -4.3 , 92.4 ) |
| Mauritius | 130.5 ( 83.5 , 178.1 ) | 887.3 ( 589.9 , 1234.1 ) | 579.7 ( 420.4 , 800.8 ) | 16.9 ( 10.6 , 23.3 ) | 49.8 ( 32.7 , 70.6 ) | 194.8 ( 124.5 , 289.5 ) |
| Mexico | 13785.1 ( 9677.0 , 17841.3 ) | 41241.3 ( 28908.8 , 54591.6 ) | 199.2 ( 157.9 , 254.4 ) | 32.0 ( 22.0 , 42.1 ) | 35.3 ( 24.4 , 47.3 ) | 10.6 ( -4.7 , 31.2 ) |
| Micronesia (Federated States of) | 22.5 ( 14.2 , 32.2 ) | 68.3 ( 42.6 , 101.5 ) | 204.2 ( 92.7 , 334.2 ) | 44.9 ( 27.7 , 65.6 ) | 86.4 ( 52.7 , 127.3 ) | 92.4 ( 24.4 , 170.6 ) |
| Monaco | 1.0 ( 0.6 , 1.5 ) | 1.5 ( 0.9 , 2.2 ) | 51.2 ( 12.3 , 101.5 ) | 1.3 ( 0.8 , 2.0 ) | 1.4 ( 0.9 , 2.1 ) | 7.3 ( -20.1 , 44.1 ) |
| Mongolia | 19.3 ( 11.3 , 29.5 ) | 68.1 ( 44.2 , 98.3 ) | 253.3 ( 158.3 , 421.4 ) | 1.8 ( 1.0 , 2.7 ) | 2.6 ( 1.7 , 3.8 ) | 45.3 ( 7.0 , 106.8 ) |
| Montenegro | 39.2 ( 27.5 , 50.5 ) | 78.9 ( 55.5 , 105.9 ) | 101.1 ( 61.4 , 158.8 ) | 6.5 ( 4.5 , 8.4 ) | 8.0 ( 5.6 , 10.7 ) | 22.7 ( -0.7 , 57.4 ) |
| Morocco | 693.1 ( 418.3 , 1061.0 ) | 3561.3 ( 2325.4 , 4969.5 ) | 413.8 ( 280.4 , 616.7 ) | 5.2 ( 3.1 , 8.1 ) | 11.8 ( 7.7 , 16.5 ) | 128.7 ( 71.4 , 216.5 ) |
| Mozambique | 239.0 ( 61.1 , 530.8 ) | 1421.7 ( 731.6 , 2194.2 ) | 494.9 ( 238.9 , 1500.2 ) | 3.9 ( 1.0 , 8.9 ) | 12.4 ( 6.3 , 19.8 ) | 217.0 ( 82.5 , 751.7 ) |
| Myanmar | 1409.3 ( 325.4 , 3367.7 ) | 6542.7 ( 3667.8 , 9823.2 ) | 364.3 ( 157.7 , 1247.5 ) | 5.7 ( 1.3 , 13.6 ) | 13.6 ( 7.4 , 20.9 ) | 140.7 ( 35.6 , 591.8 ) |
| Namibia | 150.1 ( 92.5 , 216.8 ) | 398.4 ( 265.3 , 563.5 ) | 165.4 ( 92.6 , 291.2 ) | 20.9 ( 12.8 , 30.1 ) | 30.0 ( 19.8 , 42.0 ) | 43.6 ( 5.4 , 108.2 ) |
| Nauru | 2.5 ( 1.7 , 3.5 ) | 4.2 ( 2.8 , 6.6 ) | 70.4 ( 30.5 , 122.0 ) | 58.6 ( 38.0 , 85.1 ) | 87.1 ( 54.5 , 132.8 ) | 48.6 ( 14.9 , 89.3 ) |
| Nepal | 80.3 ( 21.6 , 183.9 ) | 825.6 ( 405.3 , 1360.6 ) | 928.3 ( 499.5 , 2427.0 ) | 0.9 ( 0.2 , 2.1 ) | 4.0 ( 1.9 , 6.8 ) | 354.1 ( 167.1 , 1007.4 ) |
| Netherlands | 1197.1 ( 715.9 , 1737.2 ) | 1148.0 ( 667.7 , 1709.4 ) | -4.1 ( -17.1 , 13.3 ) | 6.0 ( 3.6 , 8.7 ) | 3.2 ( 1.9 , 4.7 ) | -46.5 ( -53.0 , -37.2 ) |
| New Zealand | 176.1 ( 115.2 , 238.0 ) | 304.0 ( 202.2 , 413.1 ) | 72.7 ( 42.4 , 106.8 ) | 4.6 ( 3.0 , 6.2 ) | 3.8 ( 2.6 , 5.1 ) | -16.0 ( -29.5 , 0.2 ) |
| Nicaragua | 156.0 ( 90.0 , 226.7 ) | 972.0 ( 639.1 , 1328.5 ) | 523.0 ( 371.0 , 787.7 ) | 10.0 ( 5.7 , 14.9 ) | 22.7 ( 14.8 , 31.3 ) | 126.3 ( 72.3 , 225.2 ) |
| Niger | 121.7 ( 48.9 , 217.8 ) | 614.8 ( 328.3 , 978.1 ) | 405.0 ( 261.0 , 716.3 ) | 4.3 ( 1.7 , 7.8 ) | 7.9 ( 4.0 , 12.9 ) | 84.5 ( 35.6 , 196.2 ) |
| Nigeria | 2552.5 ( 1113.1 , 4439.2 ) | 9055.2 ( 5487.7 , 13307.2 ) | 254.8 ( 139.1 , 497.6 ) | 5.9 ( 2.5 , 10.4 ) | 11.1 ( 6.6 , 16.5 ) | 88.0 ( 30.6 , 211.0 ) |
| Niue | 0.9 ( 0.6 , 1.4 ) | 1.5 ( 1.0 , 2.1 ) | 59.8 ( 19.6 , 114.7 ) | 43.8 ( 28.4 , 62.8 ) | 68.9 ( 44.3 , 95.8 ) | 57.2 ( 17.3 , 108.8 ) |
| North Macedonia | 179.4 ( 123.6 , 233.9 ) | 568.6 ( 390.2 , 788.3 ) | 217.0 ( 142.1 , 311.0 ) | 9.9 ( 6.7 , 13.1 ) | 18.0 ( 12.1 , 25.3 ) | 81.7 ( 38.3 , 136.4 ) |
| Northern Mariana Islands | 5.3 ( 4.0 , 6.9 ) | 18.3 ( 13.2 , 23.0 ) | 241.9 ( 166.6 , 327.5 ) | 30.4 ( 21.4 , 40.2 ) | 34.0 ( 23.9 , 44.4 ) | 11.9 ( -9.3 , 35.1 ) |
| Norway | 152.5 ( 89.4 , 225.5 ) | 206.7 ( 121.0 , 309.2 ) | 35.6 ( 15.3 , 64.1 ) | 2.2 ( 1.3 , 3.2 ) | 2.0 ( 1.2 , 2.9 ) | -9.7 ( -21.6 , 7.8 ) |
| Oman | 97.9 ( 57.3 , 146.4 ) | 426.5 ( 315.2 , 537.0 ) | 335.7 ( 206.4 , 589.7 ) | 15.1 ( 8.4 , 23.4 ) | 32.0 ( 22.5 , 42.5 ) | 111.8 ( 46.4 , 240.7 ) |
| Pakistan | 2048.5 ( 573.6 , 4187.6 ) | 18438.8 ( 10916.9 , 26871.8 ) | 800.1 ( 427.6 , 2050.4 ) | 3.7 ( 1.0 , 7.6 ) | 17.3 ( 10.0 , 25.7 ) | 370.4 ( 182.0 , 1004.0 ) |
| Palau | 4.2 ( 2.8 , 6.0 ) | 15.4 ( 10.6 , 21.0 ) | 264.0 ( 155.6 , 411.5 ) | 42.6 ( 28.0 , 61.1 ) | 69.2 ( 45.9 , 94.2 ) | 62.7 ( 15.1 , 124.5 ) |
| Palestine | 164.7 ( 96.6 , 254.7 ) | 645.6 ( 447.9 , 868.2 ) | 292.0 ( 190.9 , 453.3 ) | 19.4 ( 11.1 , 30.4 ) | 30.4 ( 20.1 , 42.5 ) | 56.9 ( 17.1 , 121.2 ) |
| Panama | 70.6 ( 30.2 , 116.7 ) | 556.0 ( 357.8 , 809.0 ) | 687.2 ( 424.8 , 1370.2 ) | 4.8 ( 2.0 , 8.0 ) | 13.4 ( 8.6 , 19.4 ) | 178.8 ( 85.1 , 426.2 ) |
| Papua New Guinea | 393.0 ( 159.7 , 687.1 ) | 1666.7 ( 851.2 , 2715.7 ) | 324.1 ( 203.7 , 555.7 ) | 18.7 ( 7.1 , 33.7 ) | 29.8 ( 14.1 , 50.3 ) | 59.2 ( 17.0 , 138.1 ) |
| Paraguay | 197.1 ( 129.4 , 275.8 ) | 1219.1 ( 785.1 , 1771.8 ) | 518.5 ( 345.5 , 751.2 ) | 9.0 ( 5.8 , 12.8 ) | 22.3 ( 14.2 , 32.4 ) | 147.1 ( 78.5 , 239.5 ) |
| Peru | 612.6 ( 353.3 , 885.9 ) | 2345.2 ( 1437.3 , 3570.3 ) | 282.8 ( 165.6 , 468.8 ) | 5.1 ( 2.9 , 7.5 ) | 7.4 ( 4.5 , 11.2 ) | 42.9 ( -0.8 , 113.3 ) |
| Philippines | 2095.9 ( 1031.9 , 3283.4 ) | 10713.0 ( 6516.9 , 15444.8 ) | 411.1 ( 270.9 , 662.9 ) | 6.6 ( 3.1 , 11.1 ) | 13.3 ( 7.9 , 19.7 ) | 101.6 ( 44.2 , 208.7 ) |
| Poland | 3023.8 ( 2200.4 , 3850.3 ) | 4191.1 ( 2932.7 , 5634.2 ) | 38.6 ( 13.2 , 65.9 ) | 6.8 ( 5.0 , 8.7 ) | 5.9 ( 4.2 , 7.9 ) | -13.6 ( -28.3 , 3.5 ) |
| Portugal | 992.4 ( 580.0 , 1445.9 ) | 1471.2 ( 845.4 , 2230.6 ) | 48.2 ( 17.5 , 89.4 ) | 6.9 ( 4.0 , 10.2 ) | 5.5 ( 3.3 , 8.0 ) | -20.8 ( -33.2 , -0.8 ) |
| Puerto Rico | 800.4 ( 543.8 , 1051.7 ) | 1728.5 ( 1145.8 , 2453.7 ) | 116.0 ( 66.9 , 177.1 ) | 22.3 ( 15.0 , 29.4 ) | 23.8 ( 16.1 , 33.5 ) | 6.8 ( -17.8 , 39.1 ) |
| Qatar | 39.9 ( 28.2 , 52.5 ) | 253.3 ( 175.8 , 345.9 ) | 535.6 ( 352.8 , 780.2 ) | 56.9 ( 36.6 , 78.3 ) | 69.0 ( 43.7 , 98.9 ) | 21.2 ( -10.0 , 61.3 ) |
| Republic of Korea | 1217.7 ( 464.9 , 2116.4 ) | 3316.1 ( 1582.0 , 5306.0 ) | 172.3 ( 105.4 , 313.6 ) | 4.0 ( 1.5 , 7.0 ) | 3.8 ( 1.8 , 6.1 ) | -4.4 ( -27.5 , 47.4 ) |
| Republic of Moldova | 164.5 ( 119.1 , 208.8 ) | 169.5 ( 123.6 , 216.7 ) | 3.1 ( -13.9 , 24.3 ) | 3.6 ( 2.6 , 4.6 ) | 2.9 ( 2.1 , 3.7 ) | -18.8 ( -32.0 , -1.7 ) |
| Romania | 1078.3 ( 831.1 , 1329.7 ) | 1359.5 ( 996.8 , 1754.0 ) | 26.1 ( 2.1 , 53.3 ) | 3.8 ( 2.9 , 4.6 ) | 3.6 ( 2.7 , 4.7 ) | -2.9 ( -21.1 , 19.2 ) |
| Russian Federation | 4180.2 ( 3102.7 , 5221.4 ) | 10573.0 ( 7554.0 , 13715.9 ) | 152.9 ( 114.0 , 198.3 ) | 2.3 ( 1.7 , 2.9 ) | 4.4 ( 3.2 , 5.7 ) | 92.7 ( 63.2 , 127.4 ) |
| Rwanda | 235.5 ( 74.4 , 464.2 ) | 559.2 ( 277.0 , 920.6 ) | 137.5 ( 54.2 , 342.0 ) | 7.9 ( 2.4 , 15.7 ) | 9.7 ( 4.7 , 16.3 ) | 23.0 ( -18.1 , 129.6 ) |
| Saint Kitts and Nevis | 11.3 ( 7.5 , 15.4 ) | 17.9 ( 12.4 , 23.9 ) | 57.8 ( 25.8 , 103.7 ) | 30.9 ( 20.9 , 42.0 ) | 27.4 ( 18.7 , 37.6 ) | -11.1 ( -27.1 , 10.7 ) |
| Saint Lucia | 27.5 ( 17.4 , 38.4 ) | 61.1 ( 41.8 , 84.8 ) | 122.5 ( 75.1 , 189.2 ) | 31.6 ( 19.8 , 44.9 ) | 28.7 ( 19.4 , 40.1 ) | -9.2 ( -28.3 , 17.4 ) |
| Saint Vincent and the Grenadines | 20.0 ( 11.2 , 29.7 ) | 51.5 ( 35.4 , 69.4 ) | 158.0 ( 101.2 , 263.7 ) | 28.2 ( 15.9 , 42.0 ) | 38.5 ( 26.3 , 52.3 ) | 36.6 ( 7.7 , 90.9 ) |
| Samoa | 34.5 ( 22.9 , 48.5 ) | 71.3 ( 47.4 , 97.4 ) | 106.8 ( 49.3 , 182.8 ) | 38.5 ( 24.7 , 55.1 ) | 48.0 ( 31.0 , 66.6 ) | 24.6 ( -9.7 , 69.0 ) |
| San Marino | 0.9 ( 0.6 , 1.4 ) | 1.8 ( 0.9 , 3.0 ) | 93.8 ( 20.7 , 186.6 ) | 2.8 ( 1.7 , 4.2 ) | 2.5 ( 1.3 , 4.2 ) | -10.3 ( -42.9 , 34.1 ) |
| Sao Tome and Principe | 1.9 ( 0.9 , 3.1 ) | 6.5 ( 4.5 , 9.0 ) | 243.3 ( 133.4 , 465.3 ) | 3.0 ( 1.4 , 4.9 ) | 6.5 ( 4.3 , 9.0 ) | 118.2 ( 52.3 , 254.1 ) |
| Saudi Arabia | 679.6 ( 434.3 , 987.6 ) | 2132.6 ( 1557.9 , 2783.1 ) | 213.8 ( 112.1 , 371.4 ) | 11.7 ( 7.3 , 17.3 ) | 12.5 ( 9.0 , 16.5 ) | 7.1 ( -24.9 , 59.3 ) |
| Senegal | 229.3 ( 119.0 , 360.7 ) | 1017.8 ( 636.6 , 1492.8 ) | 343.9 ( 208.6 , 584.3 ) | 7.2 ( 3.6 , 11.5 ) | 14.1 ( 8.7 , 21.1 ) | 95.8 ( 39.2 , 203.4 ) |
| Serbia | 1100.1 ( 752.1 , 1443.6 ) | 1922.1 ( 1269.0 , 2671.3 ) | 74.7 ( 32.4 , 129.1 ) | 10.0 ( 6.8 , 13.3 ) | 11.6 ( 7.6 , 16.2 ) | 16.0 ( -13.2 , 53.6 ) |
| Seychelles | 2.5 ( 1.5 , 3.7 ) | 10.5 ( 7.2 , 13.9 ) | 323.4 ( 232.7 , 472.0 ) | 4.4 ( 2.6 , 6.5 ) | 9.3 ( 6.2 , 12.7 ) | 111.7 ( 67.5 , 180.0 ) |
| Sierra Leone | 71.5 ( 22.8 , 138.9 ) | 335.7 ( 185.5 , 529.1 ) | 369.4 ( 196.5 , 906.7 ) | 3.8 ( 1.2 , 7.4 ) | 9.3 ( 5.0 , 14.9 ) | 145.1 ( 59.2 , 407.4 ) |
| Singapore | 85.2 ( 32.0 , 149.5 ) | 60.5 ( 37.3 , 86.3 ) | -29.0 ( -47.8 , 27.2 ) | 3.9 ( 1.4 , 6.8 ) | 0.8 ( 0.5 , 1.1 ) | -79.8 ( -85.2 , -63.0 ) |
| Slovakia | 409.3 ( 293.6 , 530.6 ) | 443.6 ( 298.2 , 613.8 ) | 8.4 ( -19.5 , 41.5 ) | 6.8 ( 4.9 , 8.8 ) | 4.7 ( 3.2 , 6.6 ) | -30.4 ( -48.1 , -9.1 ) |
| Slovenia | 122.7 ( 80.9 , 173.6 ) | 175.4 ( 109.3 , 253.5 ) | 43.0 ( -2.5 , 98.4 ) | 5.1 ( 3.4 , 7.3 ) | 3.8 ( 2.4 , 5.4 ) | -27.0 ( -49.8 , 1.8 ) |
| Solomon Islands | 47.5 ( 23.7 , 82.7 ) | 238.5 ( 151.1 , 340.5 ) | 401.9 ( 218.5 , 704.5 ) | 28.2 ( 13.4 , 50.8 ) | 61.1 ( 36.5 , 90.0 ) | 116.3 ( 40.1 , 245.3 ) |
| Somalia | 122.8 ( 29.8 , 295.7 ) | 332.0 ( 84.5 , 738.2 ) | 170.3 ( 94.4 , 328.3 ) | 4.5 ( 1.0 , 11.1 ) | 4.8 ( 1.2 , 11.3 ) | 6.8 ( -21.2 , 66.4 ) |
| South Africa | 4611.7 ( 3436.1 , 5870.1 ) | 16878.9 ( 13078.4 , 20643.8 ) | 266.0 ( 222.5 , 316.1 ) | 22.5 ( 16.6 , 29.3 ) | 40.0 ( 30.2 , 49.6 ) | 77.6 ( 56.2 , 102.3 ) |
| South Sudan | 194.2 ( 87.3 , 333.7 ) | 434.4 ( 251.2 , 682.0 ) | 123.7 ( 52.8 , 277.9 ) | 8.2 ( 3.6 , 14.1 ) | 11.8 ( 6.8 , 18.5 ) | 43.5 ( 0.3 , 132.8 ) |
| Spain | 3811.6 ( 2403.2 , 5325.6 ) | 3811.4 ( 2089.6 , 5784.5 ) | 0.0 ( -21.3 , 19.9 ) | 6.9 ( 4.3 , 9.7 ) | 3.3 ( 1.9 , 4.8 ) | -52.2 ( -58.8 , -45.0 ) |
| Sri Lanka | 523.9 ( 262.5 , 833.4 ) | 4723.0 ( 2652.8 , 7239.5 ) | 801.5 ( 485.9 , 1350.3 ) | 4.9 ( 2.4 , 8.0 ) | 19.3 ( 10.4 , 29.6 ) | 292.2 ( 152.1 , 551.7 ) |
| Sudan | 327.6 ( 171.4 , 561.5 ) | 1375.8 ( 834.6 , 2082.9 ) | 320.0 ( 184.6 , 560.0 ) | 3.5 ( 1.8 , 6.0 ) | 7.7 ( 4.6 , 11.9 ) | 121.4 ( 52.7 , 247.3 ) |
| Suriname | 38.2 ( 24.8 , 52.9 ) | 137.7 ( 93.7 , 185.8 ) | 260.3 ( 187.1 , 375.8 ) | 14.5 ( 9.3 , 20.3 ) | 22.7 ( 15.3 , 30.8 ) | 56.1 ( 23.8 , 105.6 ) |
| Sweden | 458.2 ( 267.6 , 678.5 ) | 730.5 ( 426.8 , 1079.3 ) | 59.4 ( 36.8 , 92.3 ) | 3.0 ( 1.8 , 4.4 ) | 3.2 ( 1.9 , 4.6 ) | 5.2 ( -7.5 , 24.4 ) |
| Switzerland | 521.4 ( 308.7 , 764.1 ) | 470.0 ( 272.6 , 719.2 ) | -9.9 ( -24.9 , 8.2 ) | 4.8 ( 2.9 , 6.9 ) | 2.4 ( 1.4 , 3.5 ) | -50.8 ( -57.7 , -42.3 ) |
| Syrian Arab Republic | 437.6 ( 281.5 , 624.0 ) | 938.3 ( 610.3 , 1341.2 ) | 114.4 ( 54.0 , 217.1 ) | 8.5 ( 5.3 , 12.4 ) | 8.2 ( 5.3 , 12.0 ) | -2.7 ( -29.7 , 42.7 ) |
| Taiwan (Province of China) | 1398.3 ( 714.3 , 2120.4 ) | 3354.8 ( 1804.4 , 5288.8 ) | 139.9 ( 81.1 , 229.7 ) | 8.9 ( 4.5 , 13.6 ) | 8.4 ( 4.5 , 13.3 ) | -5.6 ( -27.6 , 29.7 ) |
| Tajikistan | 106.5 ( 53.3 , 161.8 ) | 649.4 ( 382.6 , 967.8 ) | 509.5 ( 348.5 , 812.8 ) | 3.7 ( 1.8 , 5.6 ) | 11.9 ( 6.9 , 17.7 ) | 222.6 ( 137.4 , 366.8 ) |
| Thailand | 1825.5 ( 710.7 , 3146.9 ) | 7617.7 ( 4586.1 , 11497.1 ) | 317.3 ( 160.7 , 700.7 ) | 4.8 ( 1.8 , 8.5 ) | 7.4 ( 4.4 , 11.2 ) | 53.6 ( -5.0 , 203.6 ) |
| Timor-Leste | 6.7 ( 1.7 , 15.2 ) | 29.6 ( 10.7 , 58.4 ) | 340.6 ( 185.8 , 754.0 ) | 2.1 ( 0.5 , 4.8 ) | 3.5 ( 1.3 , 7.0 ) | 68.5 ( 8.7 , 229.6 ) |
| Togo | 65.5 ( 29.3 , 111.0 ) | 413.5 ( 252.8 , 624.6 ) | 531.1 ( 329.8 , 979.3 ) | 5.3 ( 2.3 , 9.2 ) | 11.6 ( 6.8 , 17.8 ) | 118.4 ( 51.4 , 267.8 ) |
| Tokelau | 0.4 ( 0.2 , 0.7 ) | 0.7 ( 0.4 , 0.9 ) | 58.9 ( 9.7 , 141.0 ) | 32.2 ( 17.9 , 49.8 ) | 49.9 ( 31.5 , 70.3 ) | 55.0 ( 7.6 , 129.2 ) |
| Tonga | 24.5 ( 16.8 , 33.4 ) | 43.9 ( 29.3 , 60.5 ) | 78.8 ( 35.5 , 136.1 ) | 42.0 ( 28.1 , 58.2 ) | 55.6 ( 36.9 , 76.8 ) | 32.5 ( 1.6 , 74.9 ) |
| Trinidad and Tobago | 525.8 ( 383.0 , 663.1 ) | 1046.8 ( 700.1 , 1475.8 ) | 99.1 ( 49.0 , 164.4 ) | 63.1 ( 45.6 , 80.3 ) | 55.5 ( 36.8 , 78.4 ) | -12.1 ( -34.0 , 16.5 ) |
| Tunisia | 212.5 ( 137.6 , 318.7 ) | 936.2 ( 580.0 , 1395.6 ) | 340.5 ( 198.4 , 534.6 ) | 4.4 ( 2.7 , 6.8 ) | 7.7 ( 4.7 , 11.8 ) | 73.9 ( 19.8 , 152.8 ) |
| Turkey | 6983.2 ( 4819.0 , 9221.4 ) | 10875.2 ( 7374.9 , 14982.4 ) | 55.7 ( 15.6 , 109.3 ) | 20.4 ( 13.9 , 27.5 ) | 12.8 ( 8.6 , 17.8 ) | -37.4 ( -53.3 , -15.8 ) |
| Turkmenistan | 106.3 ( 76.4 , 137.5 ) | 469.0 ( 330.2 , 627.2 ) | 341.1 ( 234.1 , 489.4 ) | 5.3 ( 3.7 , 6.8 ) | 11.1 ( 7.8 , 14.8 ) | 110.0 ( 60.3 , 179.5 ) |
| Tuvalu | 2.3 ( 1.2 , 3.6 ) | 5.6 ( 3.2 , 8.6 ) | 142.7 ( 69.3 , 284.9 ) | 32.1 ( 15.8 , 50.5 ) | 53.3 ( 30.2 , 82.2 ) | 66.2 ( 16.2 , 164.3 ) |
| Uganda | 303.1 ( 85.6 , 642.3 ) | 1665.4 ( 923.9 , 2574.1 ) | 449.5 ( 242.1 , 1146.5 ) | 4.7 ( 1.3 , 10.2 ) | 12.0 ( 6.4 , 19.1 ) | 155.6 ( 59.8 , 480.4 ) |
| Ukraine | 1581.6 ( 1217.5 , 1930.9 ) | 1506.1 ( 1100.1 , 1927.9 ) | -4.8 ( -20.7 , 14.8 ) | 2.2 ( 1.7 , 2.7 ) | 2.0 ( 1.5 , 2.6 ) | -6.8 ( -22.5 , 12.4 ) |
| United Arab Emirates | 116.5 ( 83.5 , 152.9 ) | 1025.9 ( 694.5 , 1429.9 ) | 780.8 ( 451.8 , 1275.9 ) | 38.2 ( 25.4 , 52.3 ) | 35.3 ( 24.0 , 48.9 ) | -7.7 ( -39.1 , 35.3 ) |
| United Kingdom | 2978.3 ( 1825.5 , 4220.6 ) | 2583.0 ( 1562.1 , 3710.3 ) | -13.3 ( -23.3 , -0.6 ) | 3.3 ( 2.0 , 4.6 ) | 2.0 ( 1.2 , 2.7 ) | -40.1 ( -45.5 , -32.5 ) |
| United Republic of Tanzania | 888.4 ( 436.3 , 1427.5 ) | 3196.9 ( 2042.3 , 4568.8 ) | 259.8 ( 156.7 , 477.6 ) | 8.2 ( 3.9 , 13.5 ) | 13.4 ( 8.4 , 19.2 ) | 62.4 ( 17.5 , 160.8 ) |
| United States of America | 21727.7 ( 14664.5 , 28682.5 ) | 41991.8 ( 30390.9 , 53081.1 ) | 93.3 ( 76.3 , 118.3 ) | 6.9 ( 4.7 , 9.0 ) | 7.5 ( 5.6 , 9.4 ) | 9.4 ( 0.5 , 22.6 ) |
| United States Virgin Islands | 17.3 ( 11.8 , 22.9 ) | 47.3 ( 34.3 , 60.9 ) | 173.3 ( 119.6 , 243.7 ) | 21.2 ( 13.8 , 28.8 ) | 25.6 ( 18.5 , 33.4 ) | 21.1 ( -1.9 , 54.6 ) |
| Uruguay | 240.2 ( 142.6 , 343.0 ) | 372.6 ( 241.8 , 518.2 ) | 55.1 ( 31.6 , 96.5 ) | 6.1 ( 3.6 , 8.7 ) | 6.8 ( 4.5 , 9.2 ) | 11.0 ( -5.2 , 41.2 ) |
| Uzbekistan | 514.1 ( 354.3 , 679.1 ) | 4696.7 ( 3275.4 , 6133.0 ) | 813.6 ( 626.5 , 1062.4 ) | 4.4 ( 3.0 , 5.8 ) | 20.6 ( 14.3 , 27.2 ) | 373.4 ( 283.5 , 494.8 ) |
| Vanuatu | 10.8 ( 5.4 , 18.1 ) | 62.9 ( 38.5 , 97.9 ) | 482.4 ( 293.7 , 838.1 ) | 15.6 ( 7.6 , 26.8 ) | 34.6 ( 20.6 , 55.0 ) | 121.6 ( 50.0 , 258.3 ) |
| Venezuela (Bolivarian Republic of) | 1387.4 ( 909.5 , 1874.3 ) | 5430.7 ( 3432.8 , 7998.4 ) | 291.4 ( 190.4 , 424.2 ) | 14.4 ( 9.2 , 19.7 ) | 18.7 ( 11.7 , 27.7 ) | 29.9 ( -3.7 , 74.5 ) |
| Viet Nam | 903.0 ( 212.6 , 2201.6 ) | 6462.1 ( 3121.7 , 10845.7 ) | 615.6 ( 317.7 , 1791.4 ) | 2.3 ( 0.5 , 5.6 ) | 7.3 ( 3.5 , 12.5 ) | 218.2 ( 87.8 , 742.3 ) |
| Yemen | 123.6 ( 49.7 , 232.1 ) | 673.3 ( 357.1 , 1093.0 ) | 444.7 ( 258.0 , 853.2 ) | 2.5 ( 1.0 , 4.7 ) | 5.0 ( 2.7 , 8.2 ) | 103.5 ( 35.3 , 259.6 ) |
| Zambia | 258.3 ( 111.7 , 436.4 ) | 1009.2 ( 622.9 , 1479.6 ) | 290.7 ( 163.5 , 610.2 ) | 8.7 ( 3.6 , 15.0 ) | 14.9 ( 9.0 , 22.2 ) | 71.6 ( 17.4 , 212.0 ) |
| Zimbabwe | 470.0 ( 302.1 , 661.0 ) | 1587.6 ( 1028.1 , 2220.7 ) | 237.8 ( 147.5 , 369.6 ) | 11.8 ( 7.5 , 16.9 ) | 23.3 ( 15.0 , 33.1 ) | 97.6 ( 46.0 , 176.0 ) |

ASMR, age-standardized mortality rate; Red represents the maximum value and green represents the minimum value

Table S3. T2D DALYs attributable to high BMI in 204 countries and territories between 1990 and 2019.

| 204 countries and territories | DALYs | | Percentage change in number of  DALYs,1990-2019 | ASDR per 100 000 people | | Percentage change in  ASDR,1990-2019 |
| --- | --- | --- | --- | --- | --- | --- |
|  | 1990 | 2019 |  | 1990 | 2019 |  |
| Afghanistan | 30948.1 ( 15928.0 , 50703.5 ) | 137641.1 ( 87167.0 , 199895.8 ) | 344.7 ( 230.3 , 542.7 ) | 396.3 ( 203.9 , 649.9 ) | 900.8 ( 574.2 , 1280.4 ) | 127.3 ( 70.2 , 228.1 ) |
| Albania | 2920.5 ( 1762.9 , 4368.0 ) | 8928.8 ( 5675.3 , 12894.7 ) | 205.7 ( 161.9 , 279.5 ) | 130.7 ( 79.2 , 193.7 ) | 219.2 ( 140.7 , 315.8 ) | 67.7 ( 43.0 , 107.4 ) |
| Algeria | 45045.8 ( 30012.0 , 65908.3 ) | 234201.6 ( 162681.2 , 321406.8 ) | 419.9 ( 325.0 , 540.1 ) | 332.9 ( 218.9 , 487.7 ) | 639.0 ( 439.4 , 872.7 ) | 91.9 ( 55.9 , 137.3 ) |
| American Samoa | 533.8 ( 418.1 , 661.4 ) | 1474.1 ( 1156.7 , 1842.2 ) | 176.1 ( 141.0 , 216.7 ) | 1953.9 ( 1507.0 , 2448.2 ) | 2814.3 ( 2193.6 , 3528.2 ) | 44.0 ( 25.0 , 64.8 ) |
| Andorra | 87.5 ( 55.0 , 129.8 ) | 313.6 ( 198.1 , 458.8 ) | 258.4 ( 200.6 , 320.2 ) | 150.7 ( 93.8 , 224.9 ) | 232.0 ( 145.7 , 338.5 ) | 53.9 ( 27.6 , 82.0 ) |
| Angola | 8400.6 ( 1946.3 , 18653.0 ) | 71467.5 ( 41479.6 , 103911.1 ) | 750.7 ( 363.9 , 2500.7 ) | 172.9 ( 39.5 , 388.1 ) | 503.1 ( 287.8 , 740.5 ) | 191.0 ( 62.1 , 788.3 ) |
| Antigua and Barbuda | 370.1 ( 224.3 , 534.3 ) | 1184.5 ( 840.7 , 1585.4 ) | 220.0 ( 163.0 , 330.5 ) | 751.0 ( 461.5 , 1068.8 ) | 1103.9 ( 783.2 , 1478.4 ) | 47.0 ( 23.3 , 92.6 ) |
| Argentina | 89406.6 ( 51826.0 , 128982.9 ) | 228384.5 ( 154648.9 , 316145.6 ) | 155.4 ( 116.0 , 233.5 ) | 272.2 ( 157.6 , 396.1 ) | 438.1 ( 297.5 , 604.6 ) | 60.9 ( 35.1 , 111.1 ) |
| Armenia | 10533.6 ( 7296.8 , 14204.9 ) | 30166.9 ( 22366.9 , 39298.0 ) | 186.4 ( 143.9 , 246.2 ) | 355.1 ( 243.8 , 483.9 ) | 724.8 ( 538.2 , 946.0 ) | 104.1 ( 71.7 , 148.7 ) |
| Australia | 38255.3 ( 25477.6 , 52359.7 ) | 105847.6 ( 72589.6 , 146200.6 ) | 176.7 ( 137.9 , 227.8 ) | 198.7 ( 134.2 , 270.9 ) | 279.5 ( 192.1 , 383.4 ) | 40.6 ( 19.6 , 67.9 ) |
| Austria | 20011.8 ( 12595.7 , 28603.2 ) | 42630.6 ( 27490.5 , 61848.8 ) | 113.0 ( 83.7 , 152.7 ) | 178.5 ( 113.1 , 253.5 ) | 269.4 ( 175.0 , 392.3 ) | 51.0 ( 29.3 , 79.0 ) |
| Azerbaijan | 15792.7 ( 10862.9 , 21255.8 ) | 70938.2 ( 51042.9 , 93964.2 ) | 349.2 ( 276.7 , 448.4 ) | 284.1 ( 194.9 , 384.0 ) | 650.1 ( 463.2 , 868.6 ) | 128.8 ( 94.1 , 178.5 ) |
| Bahamas | 1588.9 ( 1173.1 , 2042.2 ) | 4364.4 ( 3131.9 , 5757.5 ) | 174.7 ( 134.9 , 221.1 ) | 935.8 ( 682.4 , 1213.6 ) | 1030.1 ( 740.5 , 1360.3 ) | 10.1 ( -5.5 , 29.2 ) |
| Bahrain | 2541.0 ( 1877.1 , 3277.2 ) | 24240.9 ( 17836.5 , 31785.1 ) | 854.0 ( 684.0 , 1037.8 ) | 1258.8 ( 894.5 , 1649.8 ) | 2141.7 ( 1536.8 , 2838.7 ) | 70.1 ( 38.9 , 103.9 ) |
| Bangladesh | 34751.5 ( 6416.7 , 83936.7 ) | 347702.5 ( 187875.5 , 534889.7 ) | 900.5 ( 465.6 , 3309.8 ) | 62.0 ( 11.2 , 152.7 ) | 243.1 ( 129.7 , 374.2 ) | 292.3 ( 121.6 , 1244.9 ) |
| Barbados | 2753.1 ( 1812.9 , 3722.0 ) | 5395.2 ( 3878.9 , 7153.0 ) | 96.0 ( 67.5 , 138.3 ) | 1029.8 ( 692.6 , 1373.6 ) | 1157.3 ( 836.3 , 1531.7 ) | 12.4 ( -3.6 , 33.9 ) |
| Belarus | 22764.7 ( 15083.0 , 31564.7 ) | 28570.7 ( 18729.1 , 40474.2 ) | 25.5 ( 8.1 , 46.9 ) | 177.4 ( 117.6 , 246.0 ) | 193.9 ( 127.1 , 271.2 ) | 9.3 ( -5.8 , 27.6 ) |
| Belgium | 26818.9 ( 16799.6 , 38654.8 ) | 48796.7 ( 31255.5 , 73955.1 ) | 81.9 ( 55.6 , 112.9 ) | 185.0 ( 116.3 , 266.5 ) | 255.4 ( 165.4 , 379.9 ) | 38.0 ( 17.8 , 62.2 ) |
| Belize | 607.4 ( 373.0 , 865.8 ) | 3944.4 ( 3005.9 , 5005.6 ) | 549.4 ( 427.6 , 769.5 ) | 626.6 ( 384.6 , 896.5 ) | 1273.6 ( 952.0 , 1628.8 ) | 103.3 ( 66.4 , 170.6 ) |
| Benin | 5013.2 ( 2629.4 , 7780.2 ) | 31513.7 ( 21632.7 , 42604.4 ) | 528.6 ( 355.7 , 933.2 ) | 232.6 ( 119.0 , 365.4 ) | 558.9 ( 377.5 , 763.0 ) | 140.3 ( 75.9 , 285.8 ) |
| Bermuda | 374.9 ( 265.9 , 495.0 ) | 630.8 ( 434.0 , 875.1 ) | 68.2 ( 45.5 , 93.1 ) | 584.0 ( 413.7 , 772.3 ) | 541.6 ( 377.9 , 741.8 ) | -7.3 ( -20.9 , 7.8 ) |
| Bhutan | 405.3 ( 138.6 , 757.1 ) | 2574.7 ( 1593.7 , 3760.9 ) | 535.2 ( 336.2 , 1184.4 ) | 134.3 ( 45.5 , 257.2 ) | 417.6 ( 252.4 , 614.4 ) | 211.0 ( 110.8 , 534.2 ) |
| Bolivia (Plurinational State of) | 19390.2 ( 12552.6 , 27466.9 ) | 78239.9 ( 55742.9 , 104093.3 ) | 303.5 ( 213.5 , 438.3 ) | 547.8 ( 348.8 , 785.6 ) | 835.1 ( 593.5 , 1117.2 ) | 52.5 ( 18.7 , 104.4 ) |
| Bosnia and Herzegovina | 13362.4 ( 8585.4 , 18488.0 ) | 46645.2 ( 32577.9 , 63686.4 ) | 249.1 ( 189.2 , 327.5 ) | 306.4 ( 195.7 , 430.3 ) | 798.0 ( 557.3 , 1084.3 ) | 160.5 ( 115.6 , 222.7 ) |
| Botswana | 3534.4 ( 1970.0 , 5464.6 ) | 22832.1 ( 16076.0 , 30408.1 ) | 546.0 ( 338.4 , 950.2 ) | 566.6 ( 313.2 , 877.2 ) | 1529.9 ( 1061.9 , 2025.3 ) | 170.0 ( 84.4 , 342.7 ) |
| Brazil | 506736.4 ( 330795.7 , 714226.4 ) | 1565658.6 ( 1167193.3 , 2029629.8 ) | 209.0 ( 168.5 , 283.5 ) | 511.5 ( 332.5 , 726.3 ) | 644.0 ( 481.4 , 836.2 ) | 25.9 ( 8.8 , 56.9 ) |
| Brunei Darussalam | 756.4 ( 246.1 , 1425.5 ) | 3480.7 ( 2036.1 , 5012.3 ) | 360.1 ( 225.3 , 807.2 ) | 652.1 ( 211.4 , 1233.1 ) | 935.5 ( 533.2 , 1362.9 ) | 43.5 ( 3.1 , 184.1 ) |
| Bulgaria | 52371.9 ( 37748.7 , 68556.8 ) | 63313.4 ( 44182.5 , 87590.7 ) | 20.9 ( 5.3 , 37.0 ) | 413.8 ( 298.6 , 543.1 ) | 491.3 ( 347.0 , 677.0 ) | 18.7 ( 3.6 , 33.4 ) |
| Burkina Faso | 7816.4 ( 2437.2 , 15073.0 ) | 44806.1 ( 27118.6 , 65328.4 ) | 473.2 ( 283.0 , 1151.8 ) | 161.5 ( 49.6 , 315.3 ) | 418.1 ( 249.8 , 617.4 ) | 158.8 ( 75.8 , 461.4 ) |
| Burundi | 5824.4 ( 2017.8 , 11326.0 ) | 14180.2 ( 6458.0 , 24277.3 ) | 143.5 ( 76.4 , 288.9 ) | 219.6 ( 75.7 , 428.0 ) | 253.0 ( 112.7 , 438.7 ) | 15.2 ( -14.7 , 80.2 ) |
| Cabo Verde | 289.4 ( 146.2 , 463.1 ) | 2569.8 ( 1830.0 , 3449.8 ) | 788.0 ( 556.5 , 1372.2 ) | 134.3 ( 68.5 , 212.8 ) | 577.9 ( 407.7 , 785.2 ) | 330.3 ( 223.3 , 593.6 ) |
| Cambodia | 6544.9 ( 1696.1 , 14589.5 ) | 46270.0 ( 23660.8 , 74506.9 ) | 607.0 ( 341.5 , 1584.7 ) | 120.8 ( 31.4 , 268.0 ) | 342.6 ( 175.2 , 556.5 ) | 183.6 ( 79.6 , 567.9 ) |
| Cameroon | 21472.3 ( 13896.9 , 30230.1 ) | 109803.6 ( 76183.3 , 150094.3 ) | 411.4 ( 282.8 , 603.0 ) | 433.4 ( 277.4 , 624.6 ) | 813.0 ( 559.9 , 1117.6 ) | 87.6 ( 41.9 , 155.6 ) |
| Canada | 53017.8 ( 35002.5 , 73415.0 ) | 162509.4 ( 111802.1 , 225829.0 ) | 206.5 ( 158.2 , 268.6 ) | 164.8 ( 109.8 , 227.1 ) | 256.6 ( 179.4 , 351.7 ) | 55.7 ( 30.5 , 89.1 ) |
| Central African Republic | 3704.3 ( 1259.9 , 7392.7 ) | 11766.0 ( 5208.5 , 20279.4 ) | 217.6 ( 134.9 , 425.0 ) | 256.6 ( 86.0 , 512.5 ) | 415.0 ( 182.6 , 722.4 ) | 61.7 ( 20.9 , 170.4 ) |
| Chad | 3208.6 ( 1033.9 , 6160.0 ) | 21272.1 ( 11454.5 , 32380.6 ) | 563.0 ( 357.9 , 1218.6 ) | 107.3 ( 34.3 , 207.4 ) | 324.7 ( 172.1 , 502.3 ) | 202.6 ( 111.4 , 495.4 ) |
| Chile | 31486.3 ( 20031.9 , 43835.8 ) | 105490.9 ( 69348.4 , 149270.5 ) | 235.0 ( 191.8 , 294.5 ) | 303.7 ( 191.7 , 425.5 ) | 441.6 ( 291.1 , 625.2 ) | 45.4 ( 26.5 , 72.0 ) |
| China | 771801.6 ( 209707.6 , 1609362.2 ) | 3737576.0 ( 1913966.1 , 5903925.9 ) | 384.3 ( 243.9 , 886.4 ) | 80.2 ( 21.5 , 167.4 ) | 181.5 ( 93.7 , 286.3 ) | 126.3 ( 60.2 , 364.5 ) |
| Colombia | 82992.2 ( 50783.7 , 119617.6 ) | 282351.0 ( 188935.7 , 401401.6 ) | 240.2 ( 180.3 , 335.1 ) | 420.4 ( 255.8 , 611.7 ) | 535.3 ( 357.8 , 758.7 ) | 27.3 ( 4.9 , 63.2 ) |
| Comoros | 641.5 ( 267.9 , 1098.1 ) | 2101.6 ( 1312.4 , 3002.4 ) | 227.6 ( 130.0 , 516.5 ) | 264.9 ( 109.3 , 454.3 ) | 395.6 ( 242.5 , 568.9 ) | 49.3 ( 5.6 , 175.9 ) |
| Congo | 7228.6 ( 3784.2 , 11087.6 ) | 27937.2 ( 19923.5 , 36727.0 ) | 286.5 ( 171.5 , 529.4 ) | 583.3 ( 303.4 , 900.9 ) | 873.5 ( 614.6 , 1158.0 ) | 49.8 ( 8.1 , 141.4 ) |
| Cook Islands | 288.0 ( 213.5 , 370.7 ) | 576.6 ( 438.1 , 726.2 ) | 100.2 ( 61.9 , 147.0 ) | 2092.8 ( 1535.4 , 2714.7 ) | 2393.3 ( 1817.8 , 3013.2 ) | 14.4 ( -7.3 , 41.3 ) |
| Costa Rica | 7808.2 ( 5131.1 , 10998.0 ) | 29227.2 ( 18962.8 , 41867.0 ) | 274.3 ( 221.5 , 352.1 ) | 417.0 ( 268.4 , 590.0 ) | 557.7 ( 363.3 , 800.3 ) | 33.7 ( 14.9 , 63.5 ) |
| Croatia | 21622.9 ( 14411.8 , 29938.9 ) | 36799.8 ( 25090.7 , 51568.2 ) | 70.2 ( 50.5 , 94.7 ) | 334.0 ( 219.9 , 465.1 ) | 471.3 ( 321.6 , 663.1 ) | 41.1 ( 23.9 , 62.8 ) |
| Cuba | 55233.9 ( 38752.4 , 75471.2 ) | 97464.0 ( 63410.8 , 139815.0 ) | 76.5 ( 48.8 , 108.3 ) | 534.3 ( 374.0 , 730.5 ) | 556.4 ( 364.9 , 799.5 ) | 4.1 ( -12.3 , 24.0 ) |
| Cyprus | 3481.6 ( 1963.4 , 5268.7 ) | 6836.0 ( 4252.1 , 9946.6 ) | 96.3 ( 57.2 , 162.2 ) | 413.4 ( 232.8 , 626.1 ) | 357.1 ( 223.3 , 520.0 ) | -13.6 ( -31.6 , 16.9 ) |
| Czechia | 54799.8 ( 36313.6 , 76420.7 ) | 133816.3 ( 90026.6 , 187336.5 ) | 144.2 ( 116.8 , 177.9 ) | 407.3 ( 270.5 , 570.9 ) | 704.2 ( 473.9 , 979.8 ) | 72.9 ( 53.7 , 96.6 ) |
| C么te d'Ivoire | 15303.1 ( 8974.0 , 22217.4 ) | 70111.3 ( 47696.6 , 96687.5 ) | 358.2 ( 240.4 , 544.9 ) | 305.0 ( 172.1 , 456.5 ) | 537.5 ( 349.1 , 746.4 ) | 76.2 ( 32.6 , 146.6 ) |
| Democratic People's Republic of Korea | 15036.0 ( 3237.9 , 33423.7 ) | 33923.6 ( 7598.0 , 74091.1 ) | 125.6 ( 86.5 , 185.4 ) | 82.0 ( 17.7 , 182.9 ) | 102.6 ( 23.0 , 224.8 ) | 25.2 ( 3.4 , 56.9 ) |
| Democratic Republic of the Congo | 78426.5 ( 44433.9 , 117700.4 ) | 187247.8 ( 104721.1 , 279232.2 ) | 138.8 ( 85.6 , 211.5 ) | 422.9 ( 238.2 , 633.3 ) | 429.6 ( 236.4 , 643.9 ) | 1.6 ( -21.0 , 31.6 ) |
| Denmark | 10247.1 ( 6479.0 , 14340.0 ) | 22467.7 ( 14961.0 , 31040.8 ) | 119.3 ( 91.7 , 161.0 ) | 142.1 ( 90.6 , 197.8 ) | 229.1 ( 156.3 , 316.1 ) | 61.2 ( 39.6 , 93.4 ) |
| Djibouti | 235.4 ( 60.7 , 510.2 ) | 3274.7 ( 1909.8 , 4966.1 ) | 1291.0 ( 721.9 , 3573.4 ) | 127.1 ( 32.4 , 274.2 ) | 436.2 ( 246.9 , 667.3 ) | 243.1 ( 103.4 , 793.7 ) |
| Dominica | 669.4 ( 450.8 , 906.6 ) | 1227.3 ( 880.2 , 1623.6 ) | 83.3 ( 51.2 , 125.8 ) | 1024.4 ( 705.4 , 1380.4 ) | 1412.4 ( 1014.4 , 1865.4 ) | 37.9 ( 14.6 , 67.8 ) |
| Dominican Republic | 11680.6 ( 6263.1 , 17419.0 ) | 66978.4 ( 46145.5 , 91141.0 ) | 473.4 ( 323.0 , 785.4 ) | 273.2 ( 144.8 , 410.4 ) | 672.2 ( 460.1 , 913.3 ) | 146.0 ( 80.6 , 275.7 ) |
| Ecuador | 26436.0 ( 19809.0 , 33418.8 ) | 123880.8 ( 89635.8 , 160686.2 ) | 368.6 ( 298.2 , 462.5 ) | 456.8 ( 334.9 , 583.9 ) | 792.1 ( 570.8 , 1033.3 ) | 73.4 ( 46.6 , 108.8 ) |
| Egypt | 138840.2 ( 100921.3 , 178474.1 ) | 636508.2 ( 472045.4 , 829774.7 ) | 358.4 ( 270.4 , 480.3 ) | 425.5 ( 303.7 , 550.9 ) | 876.6 ( 642.4 , 1142.0 ) | 106.0 ( 66.9 , 161.6 ) |
| El Salvador | 11573.6 ( 7373.9 , 16432.6 ) | 55157.2 ( 38128.8 , 75182.7 ) | 376.6 ( 281.1 , 515.1 ) | 365.2 ( 231.3 , 518.9 ) | 947.1 ( 654.2 , 1294.2 ) | 159.3 ( 107.0 , 236.8 ) |
| Equatorial Guinea | 494.3 ( 131.3 , 1062.5 ) | 5405.5 ( 3794.6 , 7455.1 ) | 993.5 ( 455.6 , 3445.2 ) | 212.7 ( 55.7 , 458.0 ) | 947.0 ( 664.3 , 1312.5 ) | 345.2 ( 129.3 , 1322.0 ) |
| Eritrea | 1981.8 ( 664.3 , 4061.2 ) | 13381.1 ( 7119.0 , 20944.1 ) | 575.2 ( 342.2 , 1232.7 ) | 161.3 ( 56.1 , 324.4 ) | 430.0 ( 226.3 , 665.6 ) | 166.6 ( 72.1 , 429.6 ) |
| Estonia | 3646.5 ( 2470.4 , 5146.5 ) | 6693.3 ( 4537.7 , 9311.6 ) | 83.6 ( 63.9 , 108.7 ) | 183.9 ( 124.4 , 258.5 ) | 309.8 ( 213.1 , 426.4 ) | 68.4 ( 49.7 , 93.3 ) |
| Eswatini | 3893.9 ( 2741.7 , 5077.4 ) | 14463.3 ( 9897.3 , 19665.9 ) | 271.4 ( 177.8 , 411.9 ) | 1260.5 ( 878.3 , 1657.9 ) | 2349.3 ( 1605.4 , 3205.6 ) | 86.4 ( 40.6 , 156.6 ) |
| Ethiopia | 48369.8 ( 12655.9 , 104428.1 ) | 123531.1 ( 65686.5 , 190817.4 ) | 155.4 ( 55.0 , 503.1 ) | 196.9 ( 50.6 , 436.2 ) | 253.7 ( 132.3 , 399.5 ) | 28.9 ( -21.4 , 208.2 ) |
| Fiji | 11575.0 ( 8092.1 , 15634.9 ) | 40634.1 ( 30365.9 , 52014.6 ) | 251.1 ( 158.8 , 382.9 ) | 2649.3 ( 1794.5 , 3666.3 ) | 4806.7 ( 3540.3 , 6206.9 ) | 81.4 ( 33.3 , 149.5 ) |
| Finland | 13125.8 ( 8349.3 , 18946.6 ) | 27863.5 ( 17116.0 , 40660.4 ) | 112.3 ( 85.8 , 144.5 ) | 195.5 ( 125.3 , 279.8 ) | 299.1 ( 186.0 , 431.5 ) | 53.0 ( 33.1 , 78.9 ) |
| France | 78062.6 ( 47766.8 , 111277.2 ) | 177781.2 ( 118341.7 , 253958.5 ) | 127.7 ( 98.7 , 171.0 ) | 100.7 ( 62.9 , 141.0 ) | 154.1 ( 104.6 , 214.4 ) | 53.1 ( 33.0 , 83.5 ) |
| Gabon | 3605.0 ( 1888.5 , 5695.7 ) | 13488.9 ( 9695.6 , 18298.5 ) | 274.2 ( 154.0 , 516.4 ) | 589.8 ( 307.5 , 930.0 ) | 1134.3 ( 801.1 , 1532.8 ) | 92.3 ( 33.3 , 210.6 ) |
| Gambia | 780.4 ( 372.9 , 1253.5 ) | 5471.6 ( 3626.2 , 7571.6 ) | 601.1 ( 387.4 , 1140.7 ) | 193.3 ( 91.1 , 316.6 ) | 517.7 ( 337.0 , 724.4 ) | 167.9 ( 86.3 , 366.9 ) |
| Georgia | 19059.5 ( 13569.8 , 25168.3 ) | 35767.2 ( 25279.8 , 47759.1 ) | 87.7 ( 67.0 , 109.5 ) | 300.1 ( 214.2 , 395.6 ) | 648.7 ( 464.1 , 871.9 ) | 116.2 ( 92.3 , 141.5 ) |
| Germany | 349813.3 ( 226637.5 , 496863.7 ) | 568322.3 ( 370926.1 , 830592.0 ) | 62.5 ( 42.1 , 86.2 ) | 291.4 ( 189.9 , 412.8 ) | 365.4 ( 240.9 , 527.5 ) | 25.4 ( 9.2 , 45.1 ) |
| Ghana | 16854.7 ( 8244.9 , 27114.0 ) | 139305.5 ( 102680.0 , 182105.7 ) | 726.5 ( 455.5 , 1425.9 ) | 225.5 ( 107.3 , 369.6 ) | 768.7 ( 563.1 , 1010.1 ) | 240.8 ( 131.8 , 528.4 ) |
| Greece | 25076.7 ( 15178.6 , 36977.5 ) | 49742.6 ( 31126.1 , 74811.4 ) | 98.4 ( 77.2 , 125.5 ) | 169.8 ( 103.7 , 249.0 ) | 268.2 ( 168.5 , 392.8 ) | 57.9 ( 39.4 , 82.5 ) |
| Greenland | 98.3 ( 68.2 , 129.2 ) | 222.0 ( 150.9 , 304.7 ) | 125.9 ( 77.3 , 182.1 ) | 247.6 ( 169.9 , 329.0 ) | 297.3 ( 202.0 , 412.5 ) | 20.1 ( -4.5 , 48.4 ) |
| Grenada | 551.9 ( 335.6 , 788.6 ) | 1599.2 ( 1167.7 , 2081.1 ) | 189.8 ( 134.2 , 299.5 ) | 863.1 ( 533.4 , 1210.5 ) | 1351.2 ( 980.7 , 1759.4 ) | 56.6 ( 29.0 , 110.4 ) |
| Guam | 663.0 ( 473.9 , 874.3 ) | 1619.4 ( 1159.0 , 2146.7 ) | 144.2 ( 111.6 , 189.0 ) | 721.3 ( 495.0 , 958.0 ) | 844.3 ( 603.9 , 1119.1 ) | 17.1 ( -0.5 , 41.1 ) |
| Guatemala | 10096.0 ( 4474.3 , 16755.9 ) | 140972.8 ( 93355.9 , 197616.2 ) | 1296.3 ( 904.5 , 2278.7 ) | 233.7 ( 102.5 , 395.4 ) | 1171.9 ( 767.7 , 1660.2 ) | 401.5 ( 257.5 , 757.0 ) |
| Guinea | 7356.7 ( 3623.7 , 11849.0 ) | 27386.9 ( 16949.2 , 39292.1 ) | 272.3 ( 171.7 , 461.8 ) | 207.6 ( 101.3 , 337.8 ) | 447.0 ( 272.7 , 648.8 ) | 115.3 ( 58.5 , 221.9 ) |
| Guinea-Bissau | 1226.2 ( 470.1 , 2258.4 ) | 4774.8 ( 2833.0 , 7179.7 ) | 289.4 ( 172.7 , 607.6 ) | 263.9 ( 99.9 , 488.0 ) | 544.8 ( 313.3 , 833.3 ) | 106.4 ( 47.5 , 265.2 ) |
| Guyana | 5064.5 ( 3196.1 , 7153.3 ) | 13182.6 ( 9319.2 , 17753.8 ) | 160.3 ( 101.3 , 260.1 ) | 1163.8 ( 724.9 , 1663.2 ) | 1871.9 ( 1309.8 , 2546.7 ) | 60.8 ( 24.9 , 121.2 ) |
| Haiti | 24049.9 ( 10011.8 , 40386.0 ) | 64516.8 ( 33728.2 , 100903.8 ) | 168.3 ( 102.3 , 324.2 ) | 625.9 ( 258.3 , 1065.0 ) | 772.0 ( 398.3 , 1226.7 ) | 23.3 ( -6.7 , 94.4 ) |
| Honduras | 7015.9 ( 3396.7 , 11524.4 ) | 45001.9 ( 29021.7 , 65170.5 ) | 541.4 ( 402.4 , 882.2 ) | 298.5 ( 146.8 , 487.1 ) | 661.1 ( 425.1 , 965.5 ) | 121.5 ( 75.9 , 233.0 ) |
| Hungary | 55736.8 ( 40217.3 , 73952.3 ) | 90769.0 ( 63902.2 , 124641.3 ) | 62.9 ( 46.5 , 78.8 ) | 387.9 ( 278.6 , 513.6 ) | 527.9 ( 376.8 , 720.6 ) | 36.1 ( 22.2 , 49.5 ) |
| Iceland | 354.2 ( 224.4 , 526.6 ) | 1156.5 ( 733.4 , 1689.2 ) | 226.5 ( 192.6 , 265.6 ) | 130.3 ( 82.9 , 191.4 ) | 235.3 ( 148.9 , 346.0 ) | 80.6 ( 61.4 , 103.8 ) |
| India | 664180.7 ( 286164.4 , 1192460.9 ) | 5312045.5 ( 3423698.1 , 7429250.3 ) | 699.8 ( 474.4 , 1193.1 ) | 122.2 ( 51.8 , 221.4 ) | 427.6 ( 274.2 , 599.4 ) | 250.0 ( 148.9 , 474.0 ) |
| Indonesia | 237074.9 ( 88345.3 , 430603.7 ) | 1864818.9 ( 1291440.1 , 2477540.2 ) | 686.6 ( 424.8 , 1489.0 ) | 188.1 ( 67.9 , 349.3 ) | 700.4 ( 477.8 , 939.8 ) | 272.3 ( 147.6 , 666.2 ) |
| Iran (Islamic Republic of) | 74446.3 ( 49805.4 , 104142.2 ) | 473443.0 ( 344454.1 , 618503.6 ) | 536.0 ( 437.3 , 666.0 ) | 248.1 ( 165.7 , 347.3 ) | 603.4 ( 439.6 , 790.3 ) | 143.2 ( 104.8 , 197.2 ) |
| Iraq | 89792.5 ( 64543.8 , 117736.1 ) | 292072.7 ( 214002.4 , 383974.4 ) | 225.3 ( 161.2 , 303.1 ) | 1039.3 ( 734.5 , 1375.0 ) | 1108.7 ( 807.3 , 1453.0 ) | 6.7 ( -14.4 , 30.8 ) |
| Ireland | 5317.5 ( 3402.5 , 7456.6 ) | 15860.0 ( 10210.7 , 23714.3 ) | 198.3 ( 143.2 , 261.3 ) | 133.1 ( 85.5 , 185.6 ) | 227.4 ( 146.1 , 336.5 ) | 70.9 ( 39.5 , 105.9 ) |
| Israel | 12226.1 ( 7976.5 , 17002.7 ) | 41181.7 ( 27637.8 , 57649.9 ) | 236.8 ( 202.6 , 283.7 ) | 257.4 ( 167.5 , 357.4 ) | 375.9 ( 254.7 , 529.1 ) | 46.1 ( 31.8 , 66.0 ) |
| Italy | 225972.1 ( 141752.5 , 319140.4 ) | 364710.8 ( 229150.4 , 527521.5 ) | 61.4 ( 43.2 , 82.9 ) | 263.1 ( 165.1 , 370.9 ) | 303.5 ( 192.5 , 438.8 ) | 15.3 ( 1.7 , 32.4 ) |
| Jamaica | 15186.5 ( 10574.9 , 20009.4 ) | 42926.8 ( 31624.1 , 56154.2 ) | 182.7 ( 133.0 , 255.0 ) | 889.5 ( 622.0 , 1167.8 ) | 1442.8 ( 1062.3 , 1885.6 ) | 62.2 ( 34.0 , 100.8 ) |
| Japan | 150351.6 ( 57867.3 , 267774.8 ) | 228163.2 ( 92029.7 , 408543.4 ) | 51.8 ( 35.5 , 77.8 ) | 88.2 ( 33.8 , 156.9 ) | 97.2 ( 40.1 , 171.2 ) | 10.2 ( -1.8 , 30.3 ) |
| Jordan | 14520.9 ( 10706.2 , 18722.1 ) | 66816.9 ( 49067.3 , 87209.3 ) | 360.1 ( 279.4 , 469.7 ) | 999.3 ( 720.2 , 1293.5 ) | 924.6 ( 660.3 , 1195.5 ) | -7.5 ( -23.4 , 14.5 ) |
| Kazakhstan | 41243.6 ( 28457.3 , 57200.2 ) | 113281.1 ( 81021.3 , 149805.7 ) | 174.7 ( 145.8 , 210.8 ) | 305.1 ( 210.6 , 417.3 ) | 611.5 ( 434.8 , 810.3 ) | 100.5 ( 80.3 , 126.8 ) |
| Kenya | 18842.2 ( 9661.6 , 29717.1 ) | 119120.3 ( 85742.4 , 159193.2 ) | 532.2 ( 380.2 , 860.7 ) | 198.1 ( 98.5 , 318.0 ) | 448.1 ( 313.8 , 602.8 ) | 126.2 ( 71.3 , 243.2 ) |
| Kiribati | 1221.7 ( 852.1 , 1631.4 ) | 3375.9 ( 2307.8 , 4509.5 ) | 176.3 ( 105.9 , 259.6 ) | 2666.3 ( 1813.3 , 3615.1 ) | 3777.9 ( 2518.3 , 5136.1 ) | 41.7 ( 6.4 , 85.8 ) |
| Kuwait | 5627.9 ( 4145.6 , 7419.3 ) | 29193.0 ( 20618.7 , 39875.1 ) | 418.7 ( 354.7 , 489.2 ) | 699.3 ( 499.0 , 922.8 ) | 842.6 ( 590.5 , 1154.0 ) | 20.5 ( 4.5 , 37.8 ) |
| Kyrgyzstan | 5999.6 ( 4042.3 , 8316.3 ) | 14930.2 ( 10407.2 , 20425.6 ) | 148.9 ( 118.6 , 190.1 ) | 188.5 ( 126.1 , 262.7 ) | 279.3 ( 192.6 , 381.3 ) | 48.2 ( 31.2 , 70.6 ) |
| Lao People's Democratic Republic | 4742.9 ( 1412.9 , 10042.5 ) | 29594.1 ( 17121.8 , 42568.0 ) | 524.0 ( 255.0 , 1454.1 ) | 194.4 ( 57.4 , 409.8 ) | 562.6 ( 321.0 , 812.5 ) | 189.4 ( 68.2 , 604.5 ) |
| Latvia | 7005.3 ( 4763.2 , 9697.3 ) | 11551.4 ( 8099.9 , 15817.4 ) | 64.9 ( 47.0 , 86.9 ) | 203.8 ( 139.0 , 282.1 ) | 352.0 ( 251.2 , 477.8 ) | 72.7 ( 54.4 , 97.9 ) |
| Lebanon | 11002.9 ( 7504.7 , 15138.5 ) | 35261.8 ( 23320.3 , 48712.7 ) | 220.5 ( 168.4 , 280.2 ) | 450.4 ( 308.4 , 618.7 ) | 675.6 ( 446.0 , 933.8 ) | 50.0 ( 24.8 , 80.9 ) |
| Lesotho | 4942.0 ( 2978.0 , 7365.0 ) | 22683.0 ( 15157.5 , 31770.8 ) | 359.0 ( 219.3 , 595.5 ) | 475.1 ( 284.2 , 709.7 ) | 1662.6 ( 1115.8 , 2323.0 ) | 250.0 ( 146.0 , 424.3 ) |
| Liberia | 4150.2 ( 2564.3 , 6014.7 ) | 15288.6 ( 10879.3 , 20707.2 ) | 268.4 ( 180.0 , 433.8 ) | 351.0 ( 214.9 , 509.4 ) | 615.3 ( 426.5 , 850.5 ) | 75.3 ( 35.3 , 148.2 ) |
| Libya | 8815.0 ( 6146.8 , 12179.5 ) | 47495.9 ( 33524.5 , 64615.5 ) | 438.8 ( 361.9 , 540.9 ) | 425.7 ( 294.7 , 592.4 ) | 820.5 ( 576.0 , 1125.8 ) | 92.7 ( 64.7 , 129.6 ) |
| Lithuania | 7374.4 ( 4830.8 , 10706.8 ) | 11203.8 ( 7378.9 , 15671.0 ) | 51.9 ( 36.4 , 72.2 ) | 167.8 ( 110.0 , 241.2 ) | 236.4 ( 157.1 , 328.9 ) | 40.9 ( 25.9 , 60.0 ) |
| Luxembourg | 787.3 ( 528.1 , 1080.3 ) | 2972.9 ( 1890.6 , 4350.7 ) | 277.6 ( 210.2 , 350.7 ) | 149.4 ( 100.5 , 205.2 ) | 319.4 ( 202.8 , 466.9 ) | 113.8 ( 76.1 , 154.2 ) |
| Madagascar | 10496.7 ( 4624.8 , 18046.0 ) | 45638.0 ( 26116.2 , 68555.2 ) | 334.8 ( 207.4 , 586.8 ) | 176.6 ( 78.4 , 306.4 ) | 334.2 ( 187.6 , 501.7 ) | 89.3 ( 35.3 , 202.0 ) |
| Malawi | 9420.8 ( 3134.0 , 18030.4 ) | 37238.5 ( 21667.4 , 54218.8 ) | 295.3 ( 169.0 , 685.1 ) | 205.3 ( 65.3 , 404.9 ) | 443.1 ( 252.5 , 655.1 ) | 115.8 ( 44.1 , 346.3 ) |
| Malaysia | 37980.6 ( 22266.9 , 54432.9 ) | 138351.7 ( 94205.8 , 191409.8 ) | 264.3 ( 179.0 , 409.6 ) | 354.0 ( 200.4 , 517.2 ) | 468.9 ( 317.9 , 649.0 ) | 32.5 ( 0.0 , 89.2 ) |
| Maldives | 175.2 ( 50.1 , 355.6 ) | 1185.9 ( 742.9 , 1701.8 ) | 577.0 ( 310.1 , 1554.1 ) | 157.3 ( 44.3 , 326.7 ) | 302.4 ( 181.0 , 440.2 ) | 92.2 ( 17.1 , 350.4 ) |
| Mali | 7629.2 ( 2760.9 , 13742.7 ) | 41136.3 ( 25194.9 , 58782.1 ) | 439.2 ( 268.3 , 935.1 ) | 166.7 ( 59.6 , 299.4 ) | 422.1 ( 255.4 , 603.8 ) | 153.2 ( 73.9 , 374.0 ) |
| Malta | 1244.2 ( 744.5 , 1815.9 ) | 2682.0 ( 1725.1 , 3950.3 ) | 115.6 ( 82.9 , 162.5 ) | 289.6 ( 173.6 , 423.4 ) | 335.8 ( 219.3 , 489.0 ) | 16.0 ( -2.5 , 43.8 ) |
| Marshall Islands | 230.1 ( 119.3 , 356.0 ) | 1080.8 ( 700.5 , 1523.9 ) | 369.7 ( 265.5 , 583.2 ) | 1160.7 ( 585.7 , 1821.5 ) | 2362.7 ( 1489.0 , 3408.7 ) | 103.6 ( 59.3 , 194.0 ) |
| Mauritania | 4072.1 ( 2671.8 , 5752.6 ) | 11252.7 ( 7909.4 , 15217.0 ) | 176.3 ( 105.3 , 278.8 ) | 382.3 ( 249.1 , 540.9 ) | 512.0 ( 357.1 , 689.3 ) | 33.9 ( -0.1 , 83.5 ) |
| Mauritius | 5600.4 ( 3633.0 , 7665.0 ) | 32507.1 ( 22569.0 , 43753.2 ) | 480.4 ( 369.7 , 664.2 ) | 684.5 ( 439.6 , 939.9 ) | 1792.4 ( 1247.6 , 2417.2 ) | 161.9 ( 112.5 , 244.3 ) |
| Mexico | 601381.9 ( 428338.9 , 777786.0 ) | 1830570.1 ( 1339472.9 , 2357567.4 ) | 204.4 ( 171.4 , 244.0 ) | 1269.7 ( 891.2 , 1657.2 ) | 1486.8 ( 1084.0 , 1914.9 ) | 17.1 ( 4.4 , 33.3 ) |
| Micronesia (Federated States of) | 880.7 ( 603.5 , 1211.3 ) | 2773.3 ( 1862.2 , 3999.1 ) | 214.9 ( 117.6 , 323.1 ) | 1623.8 ( 1094.5 , 2239.3 ) | 3156.0 ( 2101.5 , 4532.5 ) | 94.4 ( 35.2 , 161.1 ) |
| Monaco | 69.4 ( 42.6 , 103.2 ) | 167.8 ( 103.1 , 247.5 ) | 141.8 ( 115.4 , 174.5 ) | 119.9 ( 74.5 , 178.2 ) | 224.4 ( 140.3 , 325.7 ) | 87.2 ( 67.6 , 111.1 ) |
| Mongolia | 1272.2 ( 778.0 , 1863.2 ) | 5760.4 ( 3914.2 , 8172.1 ) | 352.8 ( 270.2 , 489.9 ) | 108.3 ( 66.0 , 158.2 ) | 190.4 ( 128.9 , 269.8 ) | 75.8 ( 45.3 , 124.1 ) |
| Montenegro | 2548.9 ( 1778.6 , 3464.6 ) | 5695.9 ( 3964.8 , 7803.8 ) | 123.5 ( 102.4 , 146.2 ) | 399.9 ( 277.1 , 544.2 ) | 595.6 ( 415.8 , 813.5 ) | 48.9 ( 35.1 , 63.9 ) |
| Morocco | 40606.5 ( 26499.8 , 57220.3 ) | 218097.3 ( 147877.0 , 298535.3 ) | 437.1 ( 347.1 , 591.9 ) | 267.6 ( 171.5 , 378.9 ) | 638.3 ( 432.8 , 872.4 ) | 138.5 ( 98.2 , 206.3 ) |
| Mozambique | 9858.3 ( 2781.7 , 20627.9 ) | 65513.5 ( 37174.3 , 94398.8 ) | 564.5 ( 295.5 , 1583.7 ) | 138.9 ( 37.6 , 298.7 ) | 482.4 ( 264.9 , 708.6 ) | 247.4 ( 109.4 , 781.4 ) |
| Myanmar | 57438.3 ( 14104.3 , 134058.9 ) | 301143.7 ( 174384.0 , 431289.6 ) | 424.3 ( 191.3 , 1367.3 ) | 212.1 ( 51.5 , 497.0 ) | 577.6 ( 327.1 , 837.7 ) | 172.3 ( 53.2 , 646.3 ) |
| Namibia | 4994.6 ( 3237.3 , 6978.8 ) | 13876.6 ( 9836.8 , 18569.9 ) | 177.8 ( 112.1 , 287.4 ) | 656.0 ( 426.6 , 921.8 ) | 947.9 ( 666.9 , 1288.4 ) | 44.5 ( 11.2 , 99.3 ) |
| Nauru | 101.2 ( 71.3 , 143.3 ) | 186.9 ( 131.6 , 275.7 ) | 84.8 ( 48.8 , 128.6 ) | 1959.6 ( 1334.9 , 2718.6 ) | 3042.6 ( 2064.7 , 4400.8 ) | 55.3 ( 25.2 , 90.2 ) |
| Nepal | 6137.0 ( 1671.6 , 13251.4 ) | 67847.6 ( 37391.1 , 103086.1 ) | 1005.6 ( 600.4 , 2500.8 ) | 53.6 ( 14.5 , 117.0 ) | 275.0 ( 150.3 , 418.0 ) | 413.4 ( 225.0 , 1104.9 ) |
| Netherlands | 42575.0 ( 26060.2 , 60538.3 ) | 63604.3 ( 39289.6 , 93048.7 ) | 49.4 ( 26.7 , 79.3 ) | 221.1 ( 136.1 , 312.5 ) | 213.4 ( 131.7 , 314.8 ) | -3.5 ( -18.5 , 17.0 ) |
| New Zealand | 7139.0 ( 4732.7 , 9638.8 ) | 16966.7 ( 11637.8 , 23417.5 ) | 137.7 ( 100.2 , 184.0 ) | 190.3 ( 127.0 , 255.5 ) | 243.6 ( 169.4 , 334.3 ) | 28.0 ( 8.1 , 54.2 ) |
| Nicaragua | 8088.3 ( 4670.4 , 11934.6 ) | 47531.5 ( 32533.8 , 64663.9 ) | 487.7 ( 376.0 , 718.6 ) | 467.4 ( 268.1 , 692.0 ) | 975.4 ( 669.6 , 1324.5 ) | 108.7 ( 68.5 , 189.9 ) |
| Niger | 4451.8 ( 1849.2 , 7734.6 ) | 25507.7 ( 13907.6 , 38498.3 ) | 473.0 ( 325.7 , 799.6 ) | 135.1 ( 55.6 , 237.6 ) | 281.8 ( 150.8 , 429.9 ) | 108.5 ( 56.9 , 224.5 ) |
| Nigeria | 94858.0 ( 44531.2 , 158612.9 ) | 364011.7 ( 236585.5 , 506338.8 ) | 283.7 ( 170.9 , 519.1 ) | 197.1 ( 90.9 , 330.1 ) | 370.5 ( 234.6 , 522.6 ) | 88.0 ( 35.6 , 199.5 ) |
| Niue | 33.8 ( 23.3 , 46.2 ) | 58.4 ( 41.7 , 76.9 ) | 72.7 ( 38.4 , 117.3 ) | 1641.7 ( 1146.7 , 2240.9 ) | 2735.3 ( 1965.7 , 3601.3 ) | 66.6 ( 33.0 , 108.3 ) |
| North Macedonia | 8821.0 ( 6071.6 , 12016.0 ) | 26871.4 ( 19230.0 , 36021.1 ) | 204.6 ( 165.1 , 255.6 ) | 449.6 ( 308.4 , 615.1 ) | 819.4 ( 584.6 , 1103.7 ) | 82.2 ( 58.4 , 112.2 ) |
| Northern Mariana Islands | 283.2 ( 216.9 , 357.5 ) | 897.7 ( 675.5 , 1139.3 ) | 217.0 ( 165.0 , 271.8 ) | 1122.0 ( 842.7 , 1434.4 ) | 1470.1 ( 1093.3 , 1887.9 ) | 31.0 ( 10.7 , 51.1 ) |
| Norway | 9787.2 ( 5842.5 , 14512.4 ) | 18860.4 ( 11681.7 , 27544.5 ) | 92.7 ( 71.3 , 133.7 ) | 168.8 ( 102.3 , 250.1 ) | 229.6 ( 144.4 , 336.5 ) | 36.0 ( 21.2 , 63.3 ) |
| Oman | 4293.0 ( 2728.9 , 6014.9 ) | 22726.5 ( 17126.7 , 29135.2 ) | 429.4 ( 291.8 , 664.0 ) | 534.4 ( 322.7 , 776.8 ) | 1079.7 ( 801.7 , 1374.3 ) | 102.1 ( 49.3 , 194.5 ) |
| Pakistan | 102237.3 ( 30279.3 , 200952.9 ) | 880741.0 ( 546495.0 , 1242165.7 ) | 761.5 ( 443.3 , 1829.6 ) | 163.2 ( 48.2 , 324.3 ) | 683.3 ( 419.6 , 973.5 ) | 318.8 ( 166.8 , 840.5 ) |
| Palau | 169.5 ( 120.6 , 225.7 ) | 654.9 ( 472.0 , 849.9 ) | 286.3 ( 197.1 , 397.2 ) | 1538.4 ( 1081.7 , 2077.8 ) | 2638.4 ( 1879.5 , 3439.5 ) | 71.5 ( 32.6 , 119.3 ) |
| Palestine | 6066.3 ( 3873.9 , 8805.9 ) | 28367.6 ( 21035.4 , 37265.9 ) | 367.6 ( 272.8 , 501.3 ) | 660.3 ( 416.9 , 966.4 ) | 1089.4 ( 780.5 , 1433.9 ) | 65.0 ( 31.5 , 110.8 ) |
| Panama | 4059.4 ( 1778.2 , 6826.9 ) | 31877.3 ( 22023.2 , 44260.4 ) | 685.3 ( 466.1 , 1306.7 ) | 257.0 ( 110.9 , 430.4 ) | 764.3 ( 527.8 , 1062.5 ) | 197.4 ( 114.3 , 432.8 ) |
| Papua New Guinea | 17443.2 ( 7731.5 , 28364.3 ) | 81570.3 ( 45374.8 , 124433.9 ) | 367.6 ( 259.9 , 583.5 ) | 726.5 ( 314.7 , 1205.9 ) | 1235.1 ( 658.7 , 1939.1 ) | 70.0 ( 31.4 , 145.6 ) |
| Paraguay | 8706.7 ( 5814.7 , 12153.5 ) | 47729.7 ( 33187.4 , 64247.0 ) | 448.2 ( 343.6 , 594.1 ) | 369.8 ( 245.0 , 517.0 ) | 822.6 ( 567.6 , 1112.8 ) | 122.5 ( 79.3 , 182.0 ) |
| Peru | 27920.8 ( 16613.6 , 40497.4 ) | 121160.9 ( 81765.9 , 169159.7 ) | 333.9 ( 240.5 , 492.5 ) | 218.0 ( 128.4 , 318.8 ) | 372.6 ( 250.6 , 521.8 ) | 70.9 ( 34.0 , 134.5 ) |
| Philippines | 101325.9 ( 51073.9 , 156575.5 ) | 473362.3 ( 302226.4 , 659504.2 ) | 367.2 ( 257.1 , 575.7 ) | 267.1 ( 131.4 , 420.2 ) | 531.5 ( 334.6 , 748.3 ) | 99.0 ( 51.8 , 188.9 ) |
| Poland | 175043.5 ( 122913.3 , 232557.6 ) | 314230.7 ( 218596.7 , 424981.3 ) | 79.5 ( 61.1 , 100.3 ) | 398.9 ( 279.3 , 529.5 ) | 495.6 ( 345.0 , 669.3 ) | 24.2 ( 11.5 , 39.9 ) |
| Portugal | 39290.4 ( 22924.3 , 57595.6 ) | 73456.2 ( 46314.9 , 106569.1 ) | 87.0 ( 59.7 , 128.1 ) | 283.7 ( 165.4 , 413.8 ) | 361.3 ( 230.7 , 521.4 ) | 27.4 ( 8.8 , 57.7 ) |
| Puerto Rico | 33348.1 ( 23422.5 , 43656.7 ) | 74294.2 ( 53732.8 , 97588.4 ) | 122.8 ( 93.0 , 160.6 ) | 927.1 ( 653.5 , 1208.8 ) | 1232.3 ( 906.6 , 1614.5 ) | 32.9 ( 14.1 , 56.4 ) |
| Qatar | 1932.1 ( 1432.0 , 2489.4 ) | 24591.3 ( 17711.9 , 32950.4 ) | 1172.7 ( 945.8 , 1436.3 ) | 1552.3 ( 1104.2 , 2028.2 ) | 2085.6 ( 1487.5 , 2788.4 ) | 34.4 ( 10.4 , 65.2 ) |
| Republic of Korea | 61658.2 ( 23720.6 , 107039.0 ) | 189775.8 ( 94652.1 , 301543.3 ) | 207.8 ( 149.8 , 348.9 ) | 177.5 ( 67.6 , 312.3 ) | 216.8 ( 108.1 , 342.9 ) | 22.1 ( -2.6 , 80.5 ) |
| Republic of Moldova | 12184.4 ( 8254.4 , 16791.1 ) | 18765.6 ( 12798.4 , 25861.8 ) | 54.0 ( 34.0 , 79.9 ) | 261.1 ( 177.8 , 360.5 ) | 337.4 ( 228.8 , 462.8 ) | 29.2 ( 12.1 , 51.5 ) |
| Romania | 70688.3 ( 50549.7 , 95029.7 ) | 113583.2 ( 80581.5 , 152944.8 ) | 60.7 ( 41.8 , 82.2 ) | 247.7 ( 176.7 , 334.6 ) | 345.8 ( 245.1 , 466.9 ) | 39.6 ( 23.0 , 60.5 ) |
| Russian Federation | 299114.0 ( 209891.3 , 401006.2 ) | 606734.8 ( 432638.3 , 806485.8 ) | 102.8 ( 80.9 , 127.0 ) | 164.4 ( 114.7 , 221.1 ) | 266.7 ( 191.2 , 353.7 ) | 62.2 ( 45.7 , 81.4 ) |
| Rwanda | 8858.5 ( 2983.0 , 16928.5 ) | 23402.4 ( 12635.7 , 35835.2 ) | 164.2 ( 80.9 , 393.4 ) | 264.4 ( 86.2 , 512.6 ) | 339.0 ( 179.2 , 525.3 ) | 28.2 ( -11.4 , 138.8 ) |
| Saint Kitts and Nevis | 387.4 ( 264.9 , 522.5 ) | 867.8 ( 629.5 , 1154.0 ) | 124.0 ( 83.0 , 178.5 ) | 1127.6 ( 789.3 , 1508.2 ) | 1184.8 ( 855.4 , 1574.5 ) | 5.1 ( -11.4 , 25.4 ) |
| Saint Lucia | 1098.0 ( 714.3 , 1507.9 ) | 3076.5 ( 2170.9 , 4101.6 ) | 180.2 ( 134.7 , 249.8 ) | 1232.3 ( 804.4 , 1695.6 ) | 1403.3 ( 989.7 , 1874.6 ) | 13.9 ( -4.1 , 40.6 ) |
| Saint Vincent and the Grenadines | 723.0 ( 434.1 , 1042.0 ) | 2169.4 ( 1536.9 , 2858.8 ) | 200.0 ( 142.2 , 312.2 ) | 1014.3 ( 614.1 , 1452.9 ) | 1581.3 ( 1123.8 , 2080.7 ) | 55.9 ( 27.2 , 109.5 ) |
| Samoa | 1361.1 ( 986.8 , 1833.9 ) | 3149.9 ( 2304.5 , 4154.5 ) | 131.4 ( 79.3 , 196.2 ) | 1417.0 ( 1020.9 , 1945.2 ) | 1945.0 ( 1403.0 , 2580.2 ) | 37.3 ( 6.3 , 74.6 ) |
| San Marino | 49.3 ( 31.4 , 71.1 ) | 137.0 ( 87.2 , 204.8 ) | 178.0 ( 140.5 , 219.5 ) | 155.2 ( 100.4 , 224.7 ) | 252.6 ( 160.4 , 377.4 ) | 62.7 ( 40.7 , 87.5 ) |
| Sao Tome and Principe | 113.6 ( 60.3 , 178.3 ) | 465.8 ( 330.1 , 637.1 ) | 310.0 ( 212.0 , 520.5 ) | 167.8 ( 88.1 , 266.0 ) | 389.5 ( 269.2 , 538.4 ) | 132.2 ( 79.9 , 246.7 ) |
| Saudi Arabia | 37322.6 ( 26126.5 , 50348.5 ) | 206877.8 ( 151832.0 , 271701.9 ) | 454.3 ( 331.1 , 619.0 ) | 512.0 ( 348.9 , 710.6 ) | 802.5 ( 575.1 , 1054.9 ) | 56.7 ( 22.0 , 105.7 ) |
| Senegal | 10523.7 ( 5928.7 , 15852.1 ) | 48075.9 ( 31649.1 , 67097.4 ) | 356.8 ( 251.7 , 533.5 ) | 293.2 ( 161.2 , 448.6 ) | 579.8 ( 377.4 , 811.2 ) | 97.7 ( 51.4 , 175.1 ) |
| Serbia | 55121.0 ( 37019.4 , 74922.4 ) | 98436.5 ( 68109.6 , 133642.0 ) | 78.6 ( 54.8 , 105.9 ) | 465.2 ( 313.1 , 636.9 ) | 652.3 ( 451.4 , 879.5 ) | 40.2 ( 21.1 , 63.5 ) |
| Seychelles | 154.9 ( 96.1 , 222.7 ) | 866.3 ( 597.6 , 1226.9 ) | 459.1 ( 370.9 , 596.3 ) | 275.5 ( 171.6 , 393.9 ) | 715.4 ( 486.8 , 1002.5 ) | 159.7 ( 121.4 , 216.8 ) |
| Sierra Leone | 2402.3 ( 834.1 , 4516.9 ) | 13369.4 ( 7773.5 , 19839.2 ) | 456.5 ( 268.4 , 1023.1 ) | 119.5 ( 41.4 , 226.2 ) | 327.3 ( 188.1 , 497.8 ) | 173.9 ( 83.4 , 445.2 ) |
| Singapore | 5455.3 ( 2048.2 , 9743.3 ) | 20155.8 ( 12462.4 , 30712.6 ) | 269.5 ( 158.4 , 607.0 ) | 214.9 ( 80.0 , 386.0 ) | 244.4 ( 151.1 , 373.1 ) | 13.7 ( -21.3 , 119.9 ) |
| Slovakia | 20182.0 ( 14189.7 , 26886.0 ) | 34244.5 ( 23559.8 , 47315.4 ) | 69.7 ( 46.1 , 95.9 ) | 337.8 ( 237.2 , 451.0 ) | 386.9 ( 262.9 , 533.3 ) | 14.5 ( -1.4 , 32.8 ) |
| Slovenia | 7344.1 ( 5007.1 , 10451.9 ) | 13560.7 ( 8979.8 , 19065.4 ) | 84.6 ( 57.6 , 110.5 ) | 303.1 ( 207.3 , 431.5 ) | 359.7 ( 239.2 , 508.5 ) | 18.7 ( 1.3 , 35.8 ) |
| Solomon Islands | 2052.3 ( 1094.5 , 3385.3 ) | 10912.2 ( 7391.8 , 14998.4 ) | 431.7 ( 252.9 , 709.0 ) | 1116.9 ( 580.4 , 1860.1 ) | 2468.4 ( 1617.0 , 3438.0 ) | 121.0 ( 48.3 , 228.8 ) |
| Somalia | 5232.6 ( 1377.7 , 11866.1 ) | 15722.2 ( 4595.9 , 33037.3 ) | 200.5 ( 129.0 , 349.3 ) | 158.0 ( 39.3 , 364.4 ) | 183.8 ( 50.9 , 401.5 ) | 16.3 ( -10.6 , 75.5 ) |
| South Africa | 171195.6 ( 133621.4 , 210323.2 ) | 589314.6 ( 469691.1 , 709866.2 ) | 244.2 ( 213.5 , 279.8 ) | 764.6 ( 589.6 , 947.9 ) | 1263.6 ( 997.7 , 1533.4 ) | 65.3 ( 50.2 , 82.7 ) |
| South Sudan | 7564.2 ( 3736.8 , 12290.4 ) | 19782.6 ( 12600.2 , 28433.8 ) | 161.5 ( 94.3 , 301.9 ) | 281.6 ( 137.1 , 461.6 ) | 437.1 ( 271.2 , 632.8 ) | 55.2 ( 16.0 , 136.6 ) |
| Spain | 165386.9 ( 106701.8 , 236631.2 ) | 273573.2 ( 172560.4 , 408803.0 ) | 65.4 ( 42.9 , 92.2 ) | 308.1 ( 199.4 , 438.8 ) | 334.5 ( 211.9 , 494.7 ) | 8.6 ( -8.0 , 27.1 ) |
| Sri Lanka | 28656.9 ( 14872.6 , 44658.5 ) | 220969.0 ( 131766.0 , 320602.4 ) | 671.1 ( 467.8 , 1059.2 ) | 235.2 ( 119.0 , 371.3 ) | 841.8 ( 497.5 , 1218.8 ) | 257.9 ( 160.1 , 447.8 ) |
| Sudan | 20130.7 ( 11275.1 , 30809.3 ) | 112356.4 ( 75528.1 , 158108.8 ) | 458.1 ( 325.9 , 709.8 ) | 193.5 ( 108.0 , 297.1 ) | 513.8 ( 345.9 , 730.7 ) | 165.6 ( 104.5 , 281.7 ) |
| Suriname | 1918.8 ( 1246.5 , 2695.1 ) | 7805.2 ( 5447.2 , 10573.8 ) | 306.8 ( 246.7 , 400.5 ) | 678.4 ( 436.8 , 955.8 ) | 1231.1 ( 855.4 , 1670.5 ) | 81.5 ( 55.2 , 123.6 ) |
| Sweden | 20330.2 ( 12412.3 , 29762.9 ) | 39809.6 ( 25556.0 , 57725.3 ) | 95.8 ( 73.2 , 129.7 ) | 156.1 ( 96.1 , 226.2 ) | 232.7 ( 150.6 , 332.0 ) | 49.1 ( 30.6 , 77.9 ) |
| Switzerland | 19690.4 ( 12182.5 , 28241.1 ) | 32467.8 ( 20351.7 , 48601.3 ) | 64.9 ( 42.3 , 90.9 ) | 200.6 ( 126.0 , 287.4 ) | 216.6 ( 138.2 , 320.3 ) | 8.0 ( -7.4 , 25.4 ) |
| Syrian Arab Republic | 24562.2 ( 16500.6 , 34086.2 ) | 75042.7 ( 50968.0 , 103877.2 ) | 205.5 ( 152.8 , 275.9 ) | 408.8 ( 273.3 , 570.3 ) | 552.7 ( 374.9 , 769.6 ) | 35.2 ( 12.7 , 66.7 ) |
| Taiwan (Province of China) | 56492.2 ( 28499.1 , 85213.1 ) | 152824.8 ( 88101.7 , 228882.1 ) | 170.5 ( 124.3 , 267.8 ) | 328.5 ( 165.2 , 497.9 ) | 395.1 ( 228.8 , 592.2 ) | 20.3 ( -0.6 , 64.6 ) |
| Tajikistan | 5516.7 ( 2724.3 , 8714.3 ) | 34337.4 ( 21168.8 , 49521.2 ) | 522.4 ( 401.5 , 779.7 ) | 182.1 ( 90.7 , 287.1 ) | 535.1 ( 323.7 , 780.5 ) | 193.8 ( 138.8 , 306.6 ) |
| Thailand | 85672.6 ( 35523.7 , 140803.1 ) | 421729.7 ( 274334.5 , 598088.7 ) | 392.3 ( 240.2 , 802.2 ) | 202.5 ( 81.7 , 341.9 ) | 409.5 ( 266.7 , 580.2 ) | 102.2 ( 38.7 , 277.2 ) |
| Timor-Leste | 333.0 ( 88.0 , 719.5 ) | 1671.3 ( 646.0 , 3027.4 ) | 401.9 ( 242.1 , 874.7 ) | 84.8 ( 21.8 , 189.7 ) | 187.4 ( 72.0 , 343.5 ) | 121.2 ( 52.2 , 332.2 ) |
| Togo | 2540.8 ( 1209.8 , 4126.5 ) | 17130.7 ( 10873.0 , 24219.0 ) | 574.2 ( 382.5 , 1002.0 ) | 176.5 ( 83.5 , 290.9 ) | 400.8 ( 251.9 , 572.0 ) | 127.1 ( 66.1 , 263.9 ) |
| Tokelau | 15.3 ( 9.2 , 22.9 ) | 26.8 ( 18.7 , 36.5 ) | 75.9 ( 30.1 , 147.8 ) | 1196.3 ( 730.1 , 1765.7 ) | 1954.9 ( 1373.1 , 2655.9 ) | 63.4 ( 21.5 , 125.8 ) |
| Tonga | 936.2 ( 686.6 , 1210.0 ) | 1705.4 ( 1260.7 , 2232.9 ) | 82.2 ( 47.8 , 126.6 ) | 1511.9 ( 1101.8 , 1977.4 ) | 2085.9 ( 1532.6 , 2722.0 ) | 38.0 ( 11.9 , 71.8 ) |
| Trinidad and Tobago | 18919.7 ( 14054.4 , 23688.3 ) | 39472.6 ( 28209.7 , 52478.7 ) | 108.6 ( 69.8 , 155.4 ) | 2183.2 ( 1608.0 , 2747.4 ) | 2086.2 ( 1489.6 , 2770.6 ) | -4.4 ( -22.6 , 17.2 ) |
| Tunisia | 16251.3 ( 10838.9 , 23166.6 ) | 80350.7 ( 53935.2 , 111931.4 ) | 394.4 ( 324.5 , 490.0 ) | 296.1 ( 194.8 , 425.4 ) | 609.9 ( 407.4 , 845.0 ) | 106.0 ( 76.1 , 146.3 ) |
| Turkey | 256936.9 ( 182314.9 , 334438.7 ) | 534462.9 ( 379725.4 , 708091.3 ) | 108.0 ( 68.2 , 154.5 ) | 674.3 ( 475.8 , 884.5 ) | 590.6 ( 420.4 , 783.2 ) | -12.4 ( -29.1 , 7.2 ) |
| Turkmenistan | 6005.5 ( 4190.9 , 8058.2 ) | 27771.0 ( 20256.2 , 36349.2 ) | 362.4 ( 290.8 , 473.6 ) | 279.0 ( 194.5 , 372.5 ) | 606.3 ( 437.1 , 797.4 ) | 117.3 ( 84.4 , 167.8 ) |
| Tuvalu | 89.9 ( 49.6 , 137.0 ) | 230.9 ( 148.6 , 333.6 ) | 156.7 ( 92.4 , 277.1 ) | 1178.9 ( 645.3 , 1798.5 ) | 2091.1 ( 1336.0 , 3035.3 ) | 77.4 ( 32.9 , 161.7 ) |
| Uganda | 12552.1 ( 3843.2 , 24952.5 ) | 79810.9 ( 48148.3 , 115138.2 ) | 535.8 ( 307.9 , 1272.8 ) | 169.9 ( 50.8 , 341.5 ) | 477.0 ( 285.6 , 692.6 ) | 180.8 ( 80.1 , 509.5 ) |
| Ukraine | 137244.3 ( 98992.6 , 184179.6 ) | 171799.1 ( 119787.3 , 233998.1 ) | 25.2 ( 13.7 , 39.3 ) | 198.7 ( 142.9 , 266.0 ) | 249.5 ( 174.8 , 339.7 ) | 25.6 ( 14.3 , 39.6 ) |
| United Arab Emirates | 5776.5 ( 4255.9 , 7468.0 ) | 77450.6 ( 58054.5 , 102677.5 ) | 1240.8 ( 937.6 , 1616.9 ) | 1157.5 ( 839.8 , 1513.7 ) | 1397.7 ( 1035.0 , 1852.6 ) | 20.7 ( -7.2 , 55.5 ) |
| United Kingdom | 162591.1 ( 104016.5 , 233632.0 ) | 388827.3 ( 245677.9 , 563510.5 ) | 139.1 ( 107.2 , 176.2 ) | 199.2 ( 127.6 , 283.1 ) | 389.1 ( 245.1 , 561.5 ) | 95.3 ( 68.5 , 126.5 ) |
| United Republic of Tanzania | 31301.9 ( 15777.6 , 48331.6 ) | 132190.1 ( 91885.7 , 180726.6 ) | 322.3 ( 212.2 , 554.8 ) | 256.8 ( 127.7 , 402.8 ) | 471.8 ( 322.6 , 651.8 ) | 83.7 ( 37.2 , 186.3 ) |
| United States of America | 1144501.1 ( 787587.6 , 1526998.0 ) | 2861821.3 ( 2027340.0 , 3776854.9 ) | 150.0 ( 128.6 , 180.7 ) | 389.3 ( 271.7 , 516.3 ) | 566.2 ( 406.0 , 742.6 ) | 45.4 ( 33.1 , 63.5 ) |
| United States Virgin Islands | 742.3 ( 520.9 , 966.4 ) | 2046.3 ( 1505.0 , 2633.3 ) | 175.7 ( 136.9 , 222.1 ) | 797.7 ( 549.4 , 1050.6 ) | 1172.5 ( 862.5 , 1522.1 ) | 47.0 ( 26.0 , 75.4 ) |
| Uruguay | 6740.3 ( 4135.6 , 9482.5 ) | 14802.0 ( 10057.8 , 20382.8 ) | 119.6 ( 83.9 , 180.2 ) | 174.9 ( 107.9 , 244.8 ) | 303.9 ( 206.0 , 416.1 ) | 73.7 ( 44.3 , 124.1 ) |
| Uzbekistan | 28195.6 ( 19112.8 , 38218.8 ) | 220830.8 ( 161048.5 , 289212.4 ) | 683.2 ( 560.3 , 864.8 ) | 226.8 ( 153.5 , 307.8 ) | 840.1 ( 607.3 , 1109.7 ) | 270.4 ( 216.2 , 352.0 ) |
| Vanuatu | 522.4 ( 292.2 , 815.5 ) | 3008.6 ( 2018.3 , 4198.7 ) | 475.9 ( 332.2 , 725.1 ) | 653.9 ( 355.1 , 1035.4 ) | 1492.9 ( 980.9 , 2114.9 ) | 128.3 ( 70.0 , 227.1 ) |
| Venezuela (Bolivarian Republic of) | 65131.1 ( 43390.5 , 88828.5 ) | 264026.1 ( 179807.0 , 366348.5 ) | 305.4 ( 241.6 , 395.0 ) | 609.9 ( 400.3 , 838.9 ) | 868.2 ( 591.5 , 1210.1 ) | 42.3 ( 19.4 , 73.9 ) |
| Viet Nam | 33308.1 ( 7812.6 , 77367.4 ) | 285271.7 ( 142380.9 , 464365.8 ) | 756.5 ( 423.5 , 2025.9 ) | 79.3 ( 18.9 , 184.5 ) | 287.3 ( 141.6 , 469.3 ) | 262.4 ( 124.5 , 795.9 ) |
| Yemen | 7107.2 ( 3142.7 , 12354.7 ) | 48416.1 ( 28600.1 , 72697.6 ) | 581.2 ( 392.1 , 962.0 ) | 123.4 ( 53.1 , 216.1 ) | 303.7 ( 178.0 , 455.5 ) | 146.0 ( 78.3 , 287.0 ) |
| Zambia | 10683.1 ( 4966.6 , 17172.4 ) | 45737.7 ( 30513.7 , 63464.2 ) | 328.1 ( 205.1 , 632.3 ) | 309.3 ( 137.8 , 502.4 ) | 557.7 ( 363.8 , 783.1 ) | 80.3 ( 29.0 , 209.3 ) |
| Zimbabwe | 19292.1 ( 12860.0 , 26465.0 ) | 67026.3 ( 46118.5 , 90133.6 ) | 247.4 ( 178.1 , 342.1 ) | 426.3 ( 280.3 , 593.0 ) | 863.1 ( 590.2 , 1175.3 ) | 102.5 ( 61.7 , 160.4 ) |

DALYs, disability-adjusted life-years; ASDR, age-standardized DALY rate.; Red represents the maximum value and green represents the minimum value.

Table S4. T2D deaths change attributable to high BMI in 21GBD and 5 SDI regions, female and male, between 1990 and 2019.

| Female | | | | | | |
| --- | --- | --- | --- | --- | --- | --- |
| Locations rank 1990 | ASMR 1990 |  | Locations rank 2019 | ASMR 2019 | Percentage change in number of Deaths, 1990–2019 | Percentage change in ASMR, 1990–2019 |
| 1 Oceania | 30.1(19,47.1) |  | 1 Oceania | 47.8(31.4,67.6) | 270.9(156.3,401.5) | 58.5(9.6,113.9) |
| 2 Southern Sub-Saharan Africa | 25.3(19.1,32.3) |  | 2 Southern Sub-Saharan Africa | 41.9(32,51.4) | 238.4(193.1,287.1) | 65.2(43,88.8) |
| 3 Central Latin America | 24.1(16.9,31.5) |  | 3 Central Latin America | 23.5(16.6,31) | 187.6(143.3,238) | -2.4(-16.9,15) |
| 4 Caribbean | 20.7(14.1,27.7) |  | 4 Caribbean | 18.6(13.1,25) | 86.6(56,128) | -10.2(-24.8,10.4) |
| 5 North Africa and Middle East | 14.7(10.4,19.3) |  | 5 North Africa and Middle East | 16.3(12.2,20.8) | 170.1(130.5,229.1) | 10.5(-5.4,34.8) |
| 6 Tropical Latin America | 14.5(9.5,20.2) |  | 6 Tropical Latin America | 14.8(10.9,18.8) | 183.9(143.5,250.6) | 2.1(-12,27.7) |
| 7 Andean Latin America | 10(6.6,13.5) |  | 7 Andean Latin America | 14.2(9.9,19) | 291.3(203.7,409.2) | 42.2(10,86.5) |
| 8 Southern Latin America | 7.1(4.4,10) |  | 8 Central Asia | 14(10.6,17.5) | 319.9(259.3,389.2) | 180.8(142.5,224) |
| 9 High-income North America | 7(5.1,8.9) |  | 9 South east Asia | 14(9.4,19.1) | 498.2(306.6,988.9) | 155.2(73.4,369.1) |
| 10 Central Sub-Saharan Africa | 7(3.5,11.4) |  | 10 Western Sub-Saharan Africa | 13.5(9.1,18.8) | 330.3(221.9,549.9) | 94.4(48.3,188) |
| 11 Western Sub-Saharan Africa | 7(3.7,11.2) |  | 11 Middle SDI | 10.5(7.8,13.6) | 302.9(236.4,402) | 62.3(35.6,102.7) |
| 12 Central Europe | 6.8(5,8.5) |  | 12 Low-middle SDI | 10(6.8,13.7) | 468(317.7,733.4) | 135.9(74.8,249.1) |
| 13 Middle SDI | 6.5(4.1,9.3) |  | 13 Low SDI | 9.6(6.1,13.8) | 305.2(208.2,504.7) | 81.7(38.9,174.4) |
| 14 Eastern Sub-Saharan Africa | 6.1(2.5,11.2) |  | 14 Eastern Sub-Saharan Africa | 9.4(5.8,13.4) | 231.9(134.2,454.9) | 53.5(9.8,155.8) |
| 15 Global | 5.6(3.6,7.8) |  | 15 South Asia | 9.1(5.8,12.9) | 720.6(415.7,1361.9) | 205.7(92.4,464.2) |
| 16 High-middle SDI | 5.5(3.7,7.5) |  | 16 Central Sub-Saharan Africa | 8.3(4.7,12.8) | 177.2(107.1,300.8) | 18.6(-10.3,68.9) |
| 17 Southeast Asia | 5.5(2.2,9.9) |  | 17 Global | 7.7(5.6,10) | 189.1(154,239.3) | 38.1(21.3,63.1) |
| 18 Low SDI | 5.3(2.4,9.1) |  | 18 Southern Latin America | 7.5(5.2,9.9) | 95.6(66.2,145.2) | 4.5(-10.4,30.9) |
| 19 Western Europe | 5.2(3.5,7.3) |  | 19 High-income North America | 6.1(4.6,7.5) | 50.7(36.5,70) | -13.5(-20.6, -4.1) |
| 20 Central Asia | 5(3.7,6.3) |  | 20 Central Europe | 5.9(4.2,7.8) | 34.4(11.4,59) | -13.3(-26.3,1.5) |
| 21 High SDI | 4.9(3.4,6.6) |  | 21 High-middle SDI | 5.5(3.9,7.2) | 90.8(69.2,117.1) | 0.1(-10.2,13.2) |
| 22 Australasia | 4.4(2.9,6) |  | 2 2Eastern Europe | 4(3,5.1) | 88.1(58.3,124.1) | 54.3(30.2,82.7) |
| 23 Low-middle SDI | 4.3(2.1,7.1) |  | 23 High SDI | 3.7(2.8,4.8) | 34.2(19.5,52.1) | -24.6(-31.1, -16) |
| 24 South Asia | 3(1.2,5.5) |  | 24 Australasia | 3.5(2.4,4.7) | 80.7(44.5,126.2) | -20(-33.1, -2) |
| 25 Eastern Europe | 2.6(2,3.2) |  | 25 Western Europe | 3.1(2,4.3) | 0.4(-18.5,18.1) | -41.4(-48.8, -33.8) |
| 26 East Asia | 1.7(0.5,3.3) |  | 26 East Asia | 2.4(1.2,4) | 255.2(147.1,541.7) | 47.3(3.2,165.9) |
| 27 High-income Asia Pacific | 1.5(0.6,2.6) |  | 27 High-income Asia Pacific | 0.8(0.4,1.3) | 35.5(1.3,97.6) | -44.9(-54.6, -23.1) |
| Male | | | | | | |
| Locations rank 1990 | ASMR 1990 |  | Locations rank 2019 | ASMR 2019 | Percentage change in number of Deaths, 1990–2019 | Percentage change in ASMR, 1990–2019 |
| 1 Oceania | 30.3(14.5,51.6) |  | 1 Oceania | 48.8(27.9,75.3) | 283.9(179.1,445.3） | 60.9(18.3,126.4) |
| 2 Central Latin America | 16.7(10.6,22.9) |  | 2 Southern Sub-Saharan Africa | 33.8(24.7,43.7) | 333.7(263.3,454.7） | 133.3(93.7,203.9) |
| 3 Central Sub-Saharan Africa | 14.6(7.1,23.9) |  | 3 Central Latin America | 24.3(16.2,33.1) | 283.2(216.5,379.8） | 45.4(20.0,83.5) |
| 4 Southern Sub-Saharan Africa | 14.5(9.2,20.5) |  | 4 Central Sub-Saharan Africa | 15.6(8.3,24.2) | 141.2(77.0,249.1） | 7.0(-19.1,54.6) |
| 5 Caribbean | 11.1(6.9,15.8) |  | 5 Caribbean | 15.2(9.9,21.8) | 165.7(104.8,251.2） | 36.9(6.0,81.4) |
| 6 Tropical Latin America | 9.4(5.3,13.7) |  | 6 Tropical Latin America | 14.2(10.1,18.9) | 271.6(211.0,401.6） | 51.4(25.4,106.1) |
| 7 North Africa and Middle East | 8.2(5.2,11.6) |  | 7 Central Asia | 12.8(8.9,16.9) | 447.8(366.0,586.1） | 230.2(180.9,311.9) |
| 8 Southern Latin America | 7.3(3.9,10.8) |  | 8 Western Sub-Saharan Africa | 12.6(7.6,18.6) | 337.8(210.4,659.4） | 127.3(61.6,290.9) |
| 9 Andean Latin America | 6.5(3.9,9.2) |  | 9 North Africa and Middle East | 12.2(8.8,16.2) | 270.6(187.9,383.6） | 49.0(16.3,95.3) |
| 10 High-income North America | 6.2(3.9,8.5) |  | 1 0South east Asia | 11.9(7,17.5) | 583.1(362.3,1132.8） | 194.9(96.4,439.3) |
| 11 Eastern Sub-Saharan Africa | 6(1.8,12.5) |  | 11 Andean Latin America | 11.6(7.4,16.3) | 369.5(260.0,541.7） | 78.5(37.0,147.0) |
| 12 Central Europe | 5.7(4,7.5) |  | 12 Eastern Sub-Saharan Africa | 11.4(6.2,18) | 291.2(157.2,659.9） | 91.9(29.0,271.9) |
| 13 Western Sub-Saharan Africa | 5.5(2.2,10.1) |  | 13 Southern Latin America | 9.4(5.9,13.1) | 131.2(92.9,211.1） | 29.7(8.4,76.5) |
| 14 Australasia | 5.1(3.2,7.2) |  | 14 Middle SDI | 9.2(6.1,12.4) | 410.2(317.6,558.7） | 115.4(76.1,181.8) |
| 15 Low SDI | 4.9(1.8,9.3) |  | 15 Low-middle SDI | 8.9(5.6,12.9) | 556.3(363.0,1019.9） | 199.7(111.6,417.8) |
| 16 High SDI | 4.5(2.7,6.5) |  | 16 Low SDI | 8.5(4.6,13) | 265.2(173.0,483.5） | 74.6(31.8,180.2) |
| 17 Western Europe | 4.5(2.7,6.5) |  | 17 High-income North America | 8.4(6,10.7) | 161.1(131.5,216.7） | 37.1(21.4,66.0) |
| 18 Middle SDI | 4.3(2.3,6.8) |  | 18 South Asia | 7.7(4.4,11.6) | 684.6(391.1,1403.8） | 227.3(105.6,563.1) |
| 19 Global | 4.2(2.3,6.6) |  | 19 Central Europe | 7.4(5.2,9.8) | 83.2(53.7,117.7） | 29.1(8.1,53.9) |
| 20 High-middle SDI | 4.2(2.4,6) |  | 20 Global | 7.4(4.9,10.1) | 278.9(218.1,378.1） | 76.5(48.4,123.4) |
| 21 Southeast Asia | 4(1.4,7.9) |  | 21 High-middle SDI | 5.5(3.7,7.5) | 163.8(128.9,217.2） | 32.3(15.4,59.1) |
| 22 Central Asia | 3.9(2.5,5.3) |  | 22 High SDI | 5.3(3.7,7) | 123.5(98.5,168.0） | 16.2(3.4,41.2) |
| 23 Low-middle SDI | 3(1.1,5.8) |  | 23 Australasia | 5.1(3.4,6.9) | 125.0(90.8,173.6） | -1.2(-14.3,20.5) |
| 24 South Asia | 2.3(0.8,4.9) |  | 24 Western Europe | 3.9(2.4,5.6) | 52.6(34.2,76.0） | -13.3(-21.5,0.0) |
| 25 High-income Asia Pacific | 1.8(0.6,3.3) |  | 25 Eastern Europe | 2.9(2,3.9) | 133.0(82.6,191.1） | 79.0(41.2,127.5) |
| 26 Eastern Europe | 1.6(1.1,2.2) |  | 26 East Asia | 2.6(1.2,4.4) | 427.6(252.8,997.2） | 131.8(56.8,383.1) |
| 27 East Asia | 1.1(0.3,2.4) |  | 27 High-income Asia Pacific | 1.4(0.7,2.3) | 61.1(34.8,122.1） | -20.8(-34.3,12.7) |

ASMR, age-standardized mortality rate

Table S5. T2D DALYs change attributable to high BMI in 21 GBD and 5 SDI regions, female and male, between 1990 and 2019.

| Female | | | | | | |
| --- | --- | --- | --- | --- | --- | --- |
| Locations rank 1990 | ASDR 1990 |  | Locations rank 2019 | ASDR 2019 | Percentage change in number of DALYs, 1990–2019 | Percentage change in ASDR, 1990–2019 |
| 1 Oceania | 1101.9(741.8,1600.7) |  | 1 Oceania | 1783.5(1242.9,2403.6) | 289.8(189.8,397.3) | 61.9(19.5,105.1) |
| 2 Central Latin America | 967.1(695.6,1261.7) |  | 2 Southern Sub-Saharan Africa | 1331.8(1064.9,1616.7) | 222.2(190.4,256.2) | 56.7(40.8,73.0) |
| 3 Southern Sub-Saharan Africa | 849.7(670.6,1038.8) |  | 3 Central Latin America | 1079.1(792.8,1396.5) | 208.4(175.8,246.2) | 11.6(-0.5,26.2) |
| 4 Caribbean | 805.6(566.6,1058.4) |  | 4 Caribbean | 910.5(659.1,1209.3) | 120.6(92.7,155.9) | 13.0(-1.5,31.1) |
| 5 Tropical Latin America | 570.3(386.6,783.2) |  | 5 North Africa and Middle East | 766.2(582.7,995.2) | 253.0(211.3,303.5) | 41.3(24.9,61.5) |
| 6 North Africa and Middle East | 542.1(400.4,701.4) |  | 6 Central Asia | 690.1(514.1,894.0) | 275.8(239.5,322.4) | 132.6(111.5,159.7) |
| 7 Andean Latin America | 387.5(270.8,513.0) |  | 7 Tropical Latin America | 633.9(485.2,817.3) | 183.4(148.1,243.4) | 11.2(-2.9,35.0) |
| 8 High-income North America | 369.3(269.9,473.7) |  | 8 Andean Latin America | 594.9(444.0,762.0) | 305.3(240.8,389.7) | 53.5(28.7,86.7) |
| 9 Central Europe | 361.8(261.1,481.2) |  | 9 South east Asia | 591.4(417.2,791.2) | 505.2(328.3,960.6) | 169.1(90.2,370.2) |
| 10 Central Asia | 296.6(213.8,392.6) |  | 10 Middle SDI | 489.5(359.4,631.1) | 324.9(259.1,418.7) | 80.2(52.2,120.4) |
| 11 Central Sub-Saharan Africa | 278.5(153.4,426.6) |  | 11 Western Sub-Saharan Africa | 488.5(344.2,653.1) | 384.1(270.2,602.8) | 101.1(57.1,187.4) |
| 12 Southern Latin America | 275.6(176.9,389.4) |  | 12 High-income North America | 469.1(342.7,609.5) | 111.2(92.4,132.9) | 27.0(15.8,39.4) |
| 13 Middle SDI | 271.6(172.8,389.6) |  | 13 Low-middle SDI | 457.1(314.9,617.8) | 500.0(353.5,768.5) | 163.7(99.6,283.1) |
| 14 High-middle SDI | 256.8(173.2,351.5) |  | 14 Central Europe | 443.5(311.1,592.6) | 61.5(46.5,76.4) | 22.6(10.7,34.5) |
| 15 Global | 245.0(160.1,345.2) |  | 15 South Asia | 440.0(293.2,604.3) | 694.3(462.1,1183.7) | 229.1(130.2,445.5) |
| 16 Western Sub-Saharan Africa | 242.9(135.7,371.9) |  | 16 Low SDI | 408.7(268.7,574.0) | 359.7(256.7,575.2) | 103.8(58.6,199.0) |
| 17 High SDI | 242.3(169.2,325.9) |  | 17 Southern Latin America | 408.0(283.5,564.0) | 154.1(118.0,217.1) | 48.1(26.5,85.9) |
| 18 Western Europe | 224.2(152.6,309.1) |  | 18 Global | 407.6(295.6,540.4) | 231.2(191.8,285.1) | 66.4(46.6,93.8) |
| 19 Southeast Asia | 219.8(93.3,378.4) |  | 19 Central Sub-Saharan Africa | 383.3(238.4,552.7) | 228.9(161.7,343.6) | 37.6(10.3,83.6) |
| 20 Eastern Sub-Saharan Africa | 215.2(95.6,376.6) |  | 20 Eastern Sub-Saharan Africa | 362.5(242.8,495.2) | 275.7(172.1,519.5) | 68.4(23.0,174.1) |
| 21 Low SDI | 200.5(94.4,336.2) |  | 21 High-middle SDI | 328.4(231.5,442.3) | 126.6(107.1,150.2) | 27.9(16.7,41.7) |
| 22 Eastern Europe | 193.5(137.2,256.9) |  | 22 High SDI | 319.7(226.1,433.6) | 101.4(81.4,124.4) | 32.0(18.4,48.4) |
| 23 Australasia | 186.0(128.8,250.5) |  | 23 Western Europe | 274.9(186.3,385.6) | 58.5(39.3,79.5) | 22.6(6.8,39.5) |
| 24 Low-middle SDI | 173.3(88.0,289.7) |  | 24 Eastern Europe | 271.8(195.3,356.9) | 62.7(50.4,77.2) | 40.4(30.4,52.4) |
| 25 South Asia | 133.7(58.6,235.6) |  | 25 Australasia | 243.3(168.8,330.7) | 150.1(110.7,201.6) | 30.8(10.5,57.3) |
| 26 East Asia | 93.7(28.9,185.3) |  | 26 East Asia | 170.1(88.4,271.3) | 307.7(197.4,642.1) | 81.5(32.4,229.8) |
| 27 High-income Asia Pacific | 88.2(36.1,153.6) |  | 27 High-income Asia Pacific | 105.2(49.6,175.0) | 92.1(65.1,148.0) | 19.3(1.4,54.5) |
| Male | | | | | | |
| Locationsrank1990 | ASDR1990 |  | Locationsrank2019 | ASDR2019 | Percentage change in number of DALYs, 1990–2019 | Percentage change in ASDR,1990–2019 |
| 1 Oceania | 1087.3(564.5,1753.8) | | 1 Oceania | 1818.3(1107.9,2684.3) | 304.5(212.3,448.3) | 67.2(29.4,126.0) |
| 2 Central Latin America | 747.3(478.1,1033.3) |  | 2 Central Latin America | 1144.2(791.9,1549.3) | 285.9(238.2,362.8) | 53.1(33.3,84.7) |
| 3 Caribbean | 530.3(336.1,750.5) |  | 3 Southern Sub-Saharan Africa | 1090.0(828.3,1368.1) | 309.1(250.3,406.0) | 113.5(82.7,166.1) |
| 4 Southern Sub-Saharan Africa | 510.6(344.2,694.0) |  | 4 Caribbean | 814.9(553.5,1114.1) | 189.8(140.1,254.4) | 53.7(27.9,88.5) |
| 5 Central Sub-Saharan Africa | 495.3(249.7,785.6) |  | 5 Tropical Latin America | 660.6(476.8,867.6) | 253.9(198.1,379.3) | 51.2(26.5,104.7) |
| 6 Tropical Latin America | 436.8(255.4,648.7) |  | 6 North Africa and Middle East | 655.2(467.6,874.2) | 379.3(301.1,493.9) | 88.4(56.9,133.2) |
| 7 High-income North America | 361.3(228.2,500.1) |  | 7 Central Asia | 612.6(426.4,813.8) | 378.8(320.3,482.8) | 178.9(145.9,235.6) |
| 8 Central Europe | 355.7(242.8,485.8) |  | 8 Central Sub-Saharan Africa | 608.7(337.5,904.0) | 200.0(131.4,310.3) | 22.9(-3.7,67.4) |
| 9 North Africa and Middle East | 347.8(224.8,480.8) |  | 9 High-income North America | 602.8(424.1,804.3) | 203.8(171.1,264.0) | 66.9(48.9,99.8) |
| 10 Andean Latin America | 275.4(168.8,394.1) |  | 10 Central Europe | 567.1(392.8,774.7) | 109.2(87.8,133.7) | 59.4(42.9,79.8) |
| 11 Southern Latin America | 264.8(146.4,390.5) |  | 11 Andean Latin America | 526.1(360.4,714.8) | 385.9(295.3,529.5) | 91.0(55.0,150.3) |
| 12 High SDI | 249.3(153.3,358.5) |  | 12 Southeast Asia | 513.1(311.8,728.5) | 581.8(373.3,1094.9) | 205.3(111.8,439.5) |
| 13 Western Europe | 220.8(133.9,319.0) |  | 13 Middle SDI | 462.8(318.1,628.8) | 422.2(325.0,584.7) | 135.0(90.9,211.0) |
| 14 Central Asia | 219.7(141.0,310.2) |  | 14 Southern Latin America | 456.4(292.3,640.6) | 196.8(148.7,293.8) | 72.3(43.5,129.3) |
| 15 High-middle SDI | 210.8(126.1,309.7) |  | 15 Western Sub-Saharan Africa | 435.0(281.0,619.9) | 359.7(237.9,657.5) | 129.7(69.8,277.1) |
| 16 Australasia | 209.2(137.4,286.0) |  | 16 Low-middle SDI | 428.8(271.0,606.3) | 604.2(401.8,1080.7) | 235.7(139.7,466.1) |
| 17 Global | 201.0(112.2,311.5) |  | 17 South Asia | 416.5(249.8,595.5) | 745.3(466.5,1496.8) | 283.6(155.7,631.8) |
| 18 Eastern Sub-Saharan Africa | 198.1(64.4,392.1) |  | 18 Global | 413.4(277.5,564.4) | 312.9(249.3,417.9) | 105.7(73.6,159.2) |
| 19 Middle SDI | 197.0(106.5,312.8) |  | 19 High SDI | 411.9(277.9,568.1) | 181.4(150.9,235.3) | 65.3(46.1,98.5) |
| 20 Western Sub-Saharan Africa | 189.3(80.5,324.5) |  | 20 Eastern Sub-Saharan Africa | 406.0(235.1,607.3) | 342.6(200.8,747.1) | 104.9(41.3,287.9) |
| 21 Low SDI | 179.0(68.2,333.1) |  | 21 Low SDI | 364.5(206.0,550.0) | 339.6(231.1,588.5) | 103.6(54.4,220.7) |
| 22 Southeast Asia | 168.0(62.7,314.9) |  | 22 High-middle SDI | 346.4(231.2,479.1) | 206.0(168.9,261.3) | 64.3(43.8,95.2) |
| 23 Eastern Europe | 144.1(94.3,203.4) |  | 23 Western Europe | 327.5(205.5,480.7) | 117.6(93.3,152.4) | 48.3(30.0,75.2) |
| 24 Low-middle SDI | 127.7(50.4,241.4) |  | 24 Australasia | 306.0(209.1,422.1) | 191.4(149.0,253.3) | 46.3(24.6,79.0) |
| 25 High-income Asia Pacific | 121.8(44.2,221.0) |  | 25 Eastern Europe | 240.0(166.8,326.9) | 99.7(65.7,138.3) | 66.5(39.2,99.9) |
| 26 South Asia | 108.6(39.1,216.0) |  | 26 East Asia | 198.3(101.3,313.9) | 433.4(269.5,1027.0) | 162.2(81.3,457.6) |
| 27 East Asia | 75.6(20.3,161.7) |  | 27 High-income Asia Pacific | 161.2(74.8,270.0) | 110.9(83.5,178.2) | 32.3(12.2,80.9) |

DALYs, disability-adjusted life-years; ASDR, age-standardized DALY rate.

Table S6. Decomposition analysis of the contribution of population growth and population aging on the global T2D burden due to high BMI.

| Global death | | | | | | | | |
| --- | --- | --- | --- | --- | --- | --- | --- | --- |
|  |  | Contribution factors | | | |  | |  |
|  | Total death | Aging | Population | Epidemiological change | Overll difference | |  |  |
| Global | 619494.8 | 86982.31(20.3%) | 186758.856(43.59%) | 154751.22(36.12%) | 428492.386 | |  |  |
| Global DALYs | | | | | | | | |
|  | Total DALYs | Aging | Population | Epidemiological change | Overll difference | |  |  |
| Global | 34422224.8 | 3419747.516(13.66%) | 9973207.492(39.84%) | 11638380.411(46.5%) | 25031335.42 | |  |  |

FigureS1.The attributable proportions of type 2 diabetes deaths and disability-adjusted life-years (DALYs) attributed to high BMI, both sexes, globally and by 21 GBD regions, in 2019.


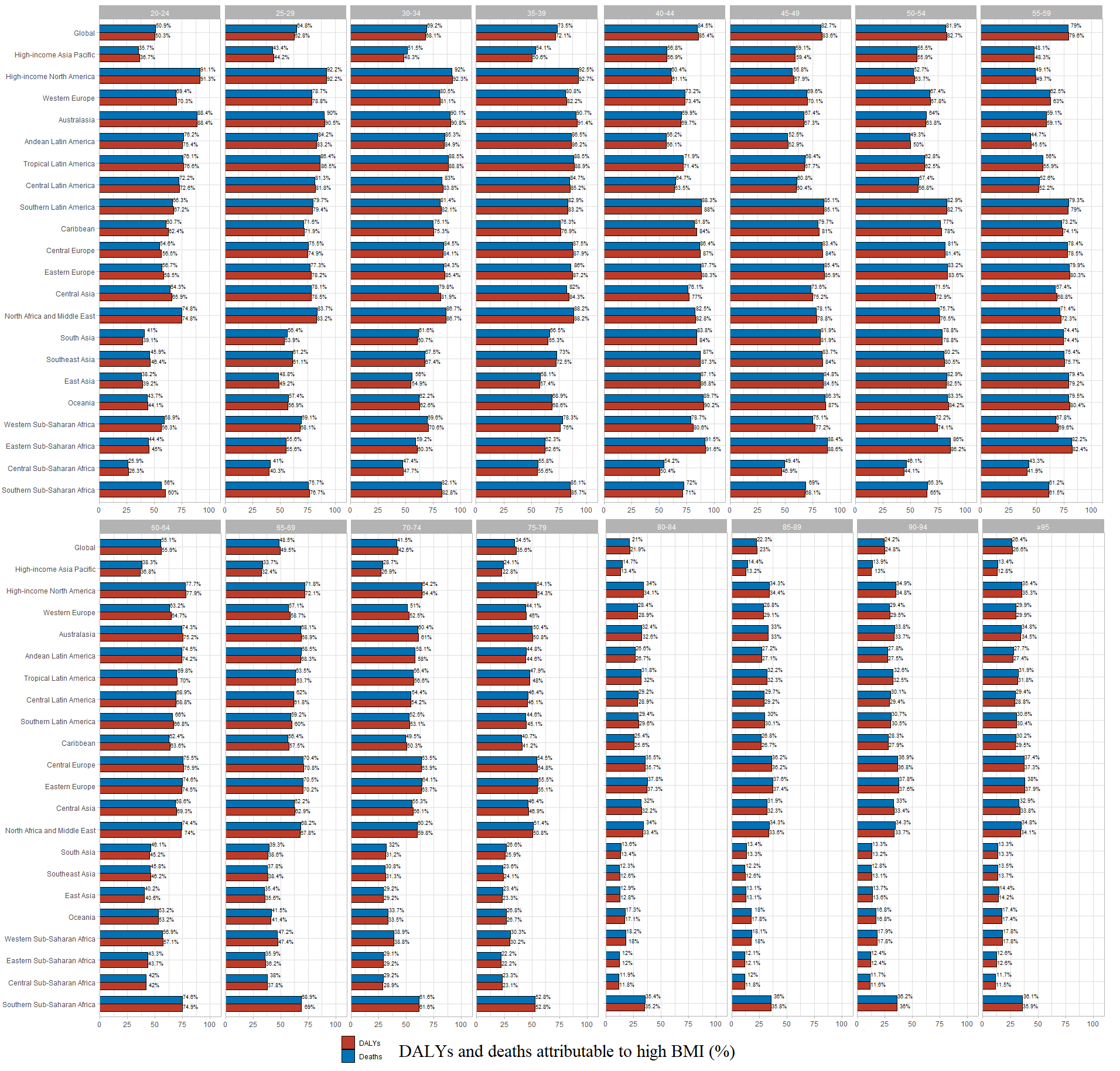

Supplement: Supplementary file 1 [file Data_Sheet_1.docx]
